# Differentiation-Associated MicroRNA Alterations in Mouse Heart-Derived Sca-1^+^CD31^−^ and Sca-1^+^CD31^+^ Cells

**DOI:** 10.1155/2016/9586751

**Published:** 2016-05-19

**Authors:** Qiong Wu, Jinxi Zhan, Yun Li, Xiaoxia Wang, Lu Xu, Juan Yu, Shiming Pu, Zuping Zhou

**Affiliations:** ^1^School of Life Sciences, Guangxi Normal University, Guilin 541004, China; ^2^Guangxi Universities Key Laboratory of Stem Cell and Biopharmaceutical Technology, Guangxi Normal University, Guilin 541004, China; ^3^Research Center for Biomedical Sciences, Guangxi Normal University, Guilin 541004, China; ^4^Jilin Medical College, Jilin 132013, China

## Abstract

Cardiac resident stem/progenitor cells (CSC/CPCs) are critical to the cellular and functional integrity of the heart because they maintain myocardial cell homeostasis. Several populations of CSC/CPCs have been identified based on expression of different stem cell-associated antigens. Sca-1^+^ cells in the cardiac tissue may be the most common CSC/CPCs. However, they are a heterogeneous cell population and, in transplants, clinicians might transplant more endothelial cells, cardiomyocytes, or other cells than stem cells. The purposes of this study were to (1) isolate CSC/CPCs with Lin^−^CD45^−^Sca-1^+^CD31^−^ and Lin^−^CD45^−^Sca-1^+^CD31^+^ surface antigens using flow-activated cell sorting; (2) investigate their differentiation potential; and (3) determine the molecular basis for differences in stemness characteristics between cell subtypes. The results indicated that mouse heart-derived Sca-1^+^CD31^−^ cells were multipotent and retained the ability to differentiate into different cardiac cell lineages, but Sca-1^+^CD31^+^ cells did not. Integrated analysis of microRNA and mRNA expression indicated that 20 microRNAs and 49 mRNAs were inversely associated with Sca-1^+^CD31^−^ and Sca-1^+^CD31^+^ subtype stemness characteristics. In particular, mmu-miR-322-5p had more targeted and inversely associated genes and transcription factors and might have higher potential for CSC/CPCs differentiation.

## 1. Introduction

Cardiac resident stem/progenitor cells (CSC/CPCs) are critical to the cellular and functional integrity of the heart. The discovery of CSC/CPCs in the postnatal heart has marked a new era of cardiac regenerative medicine. In recent years, different populations of cardiac stem or progenitor cells have been reported to reside within the adult heart. To date, at least seven distinct populations of CSC/CPCs have been identified, including stem cell antigen-1-positive (Sca-1^+^) cells [1]; side population cells [2]; and c-kit-positive (c-kit^+^) cells [3], also known as CD117 or SCFR cells, which are commonly used as stem cell surface markers and are suggested to be endothelial markers [4]; Wilms' tumor1-positive (WT1^+^) epicardial progenitor cells [5]; islet-1-positive (Isl-1^+^) cells [6]; cardiosphere-derived cells (CDCs) [7]; and mesenchymal stem cell antigen-1 (W8B2^+^) cells [8]. CSC/CPCs were identified based on expression of stem cell-associated antigens. However, no single surface marker can conclusively identify cardiac stem/progenitor cells. Although the origin and the function of these cells remain unclear, individual CSC/CPCs populations most likely represent different developmental or physiological stages of a unique CSC/CPCs population in the adult mammalian heart [3].

Sca-1^+^ cells in cardiac tissue may be the most common CPCs or predominate over the long term and thus may be relatively easy to isolate from cardiac tissue [9]. Sca-1 positive CSCs are 70% of cells in the mouse heart after depletion of cardiomyocytes. Sca-1^+^ cells are 100- to 700-fold more frequent than c-kit^+^ cells [10, 11]. However, despite the presence of abundant numbers of Sca-1^+^ cells in the heart, only a small subset of Sca-1^+^ cells differentiate into cardiomyocytes [12]. Previous studies suggested that Sca-1^+^ cardiac stem cells could be divided into Sca-1^+^CD31^−^ and Sca-1^+^CD31^+^ cells [13]. Data on the number and functional differentiation of the two populations of cells are conflicting. For instance, Pfister [13] reported that Sca-1^+^CD31^−^ cells show cardiomyogenic differentiation and Sca-1^+^CD31^+^ cells do not. Immunofluorescence (IF) staining shows that few cells express CD31 in Sca-1^+^-enriched populations. This result indicates that isolated mouse heart-derived Sca-1^+^ cells represent a Sca-1^+^CD31^−^ subpopulation. However, Liang et al. showed that Sca-1^+^CD31^+^ cells are 66.3% of a cardiac side population (CSP) but Sca-1^+^CD31^−^ is only 11.2%. CSP cells are approximately 1.0% of total heart cells [14]. Sca-1^+^CD31^+^ cells express stem cell-specific and endothelial-specific genes. These cells proliferate, differentiate, migrate, and vascularize* in vitro* and* in vivo* [14]. Other reports show that Lin^−^Sca-1^+^CD31^−^ cardiac-derived progenitors have the potential to differentiate into cardiomyogenic and mesenchymal cell lineages [15]. Lin^−^Sca1^+^CD31^+^ bone marrow endothelial progenitor cells show efficient differentiation into cardiomyocytes [16]. Clearly, many aspects about these cells remain to be understood, especially the molecular basis for differences between subtypes in stemness characteristics.

MicroRNAs (miRNA) are small, noncoding RNA molecules that regulate gene expression at the posttranscriptional level. Recent studies demonstrate the importance of miRNAs in regulating cardiac stem cell proliferation and differentiation and other physiological and pathological processes related to stem cell function [17].

This study systematically characterized mouse heart-derived Sca-1^+^CD31^−^ and Sca-1^+^CD31^+^ cells. We examined their* in vitro* differentiation properties and potential contamination by other cell types such as cardiac fibroblasts and mast cells. We compared miRNA and mRNA expression profiling for Sca-1^+^CD31^−^ versus Sca-1^+^CD31^+^ cells, integrating analysis of miRNA and mRNA data for a reliable set of miRNA target relationships for differentiation states. The overall goal of this work was to provide information required for optimizing the use of CSC/CPCs for treating cardiovascular diseases and provide a deeper understanding of the mechanisms by which miRNAs regulate cardiac stem cell differentiation in Sca-1^+^CD31^−^ and Sca-1^+^CD31^+^ cells.

## 2. Materials and Methods

### 2.1. Experimental Animals and Ethics Statement

Two-month-old C57BL/6J mice (Experimental Animal Center of Guilin Medical College, China) were used for cell isolation. All procedures involving animals were approved by the Institutional Animal Care and Use Committee.

### 2.2. Cell Isolation and Fluorescence-Activated Cell Sorting

Whole hearts were extracted from either male or female C57BL/6J mice, split, and washed several times with ice-cold PBS to remove residual blood. Hearts were minced and digested with 0.1% collagenase type II (Life) at 37°C for 30 minutes and then filtered through a 40 *μ*m cell strainer (BD Falcon). Filtered cardiac cell suspensions were centrifuged at 300 ×g for 5 min at 4°C to remove blood cells and debris. Cultures were resuspended in cold DMEM/F12 medium (Gibco) supplemented with 10% FBS (Gibco) and penicillin and streptomycin (Gibco). A single cell suspension was obtained and incubated for 30 min at 4°C with indicated combinations of monoclonal antibodies against mouse antigens Sca-1-PE-Cy7, CD31-APC, CD45-FITC, and Lin-PE (eBioscience) or with isotype-matched control antibodies before analysis by fluorescence-activated cell sorting (FACS).

FACS and analyses used a FACS Aria II flow cytometer (BD). For all FACS-sorted cell populations, purity was ensured by double sorting and subsequent FCM analyses.

### 2.3. Cell Culture and Differentiation

Sca-1^+^CD31^−^ and Sca-1^+^CD31^+^ cells were initially seeded at 1 × 10^4^ cells/cm^2^ in normal medium containing DMEM/F12 supplemented with 2% FBS, 2% B27 (Gibco), 20 ng/mL endothelial growth factor (Peprotech), 40 ng/mL fibroblast growth factor-basic (Peprotech), and 20 ng/mL leukemia inhibitory factor (Peprotech) on gelatin-coated dishes. For cardiomyocyte induction, cells were cultured with DMEM/F12 supplemented with 10% FBS, 100 ng/mL bone morphogenetic protein-2 (Peprotech), and 100 ng/mL fibroblast growth factor-4 (Peprotech) on gelatin-coated dishes for 14 days. For smooth muscle cell induction, cells were cultured in DMEM/F12 supplemented with 10% FBS with 50 ng/mL platelet-derived growth factor-BB (Peprotech) for 14 days. For endothelial cell induction, cells were cultured in DMEM/F12 supplemented with 10% FBS plus 20 ng/mL vascular endothelial growth factor-165 (VEGF_165_, Peprotech) and grown on gelatin-coated dishes for 14 days. In control groups, cells were cultured in the same medium for differentiation without growth factors. Control and treatment media were replenished for two days to ensure cytokine and growth factor activity. When induction was complete, lineage-specific markers were analyzed by IF staining.

### 2.4. RNA Preparation and Quality Control

Total cellular RNA was extracted from 3 × 10^6^ cells of Sca-1^+^CD31^−^ or Sca-1^+^CD31^+^ cells per sample, isolated using FACS, from 40 mice as a pool. RNA was extracted using TRIzol reagent (Invitrogen) according to the manufacturer's instructions. RNA concentrations were determined photometrically using a NanoDrop 1000 (Peqlab, Erlangen, Germany). Overall RNA quality was assessed by 1% agarose gels with 1 kb molecular weight marker separated in parallel. Total RNA was simultaneously used for miRNA and mRNA microarrays.

### 2.5. Droplet Digital PCR Assays

Droplet digital PCR (dd-PCR) is a direct method for quantitatively measuring nucleic acids that is more sensitive than real time RT-PCR for resolving copy number changes of DNA and RNA targets [18]. For miRNA, reverse transcription of RNA used miScript Reverse Transcription Kits. The cDNA was the template for the droplet digital PCR analysis. For mRNA, total RNA was reversed transcribed using OneStep RT-PCR Kits and used as the template for dd-PCR analysis. PCR was performed in 20 *μ*L containing 10 *μ*L 2x EvaGreen supermix (Bio-Rad), 8 *μ*L diluted cDNA, and primer sets.

Droplet digital PCR was performed as follows. Assay mixtures (20 *μ*L) were loaded into disposable droplet generator cartridges (Bio-Rad) and 70 *μ*L droplet generation oil for probes (Bio-Rad) was loaded into the eight oil wells. Cartridges were placed inside the QX200 droplet generator (Bio-Rad). When droplet generation was complete, droplets were transferred to 96-well PCR plates using a Rainin multichannel pipet. Plates were heat-sealed with foil and placed in a conventional thermal cycler. Thermal cycling conditions were 95°C for 10 min, 40 cycles of 95°C for 30 s, and 58°C for 1 min (ramping rate reduced to 2%), and 4°C for 5 min, 90°C for 5 min, and 4°C indefinite hold. A no-template control and a negative control for each reverse transcription reaction were included in every assay. All reactions were in triplicate. Relative expression of miRNAs was calculated based on U6 RNA levels and relative expression of mRNAs was calculated based on GAPDH levels.

### 2.6. MiRNA Profiling by Microarray Technology

MiRNA expression profiling by microarray technology was performed using Affymetrix GeneChip miRNA Arrays v. 4.0 (Affymetrix, USA) following the manufacturer's instructions.

Raw data were normalized using quantile normalization and analyzed in GeneSpring GX (zcom Silicon Genetics, USA). Statistical analysis using ANOVA was conducted to compare differentially expressed miRNAs.

### 2.7. Whole Genome Gene Expression Microarrays

Gene expression profiling was performed for triplicate samples with GeneChip Mouse Gene 1.0 ST Arrays (Affymetrix, USA) following the manufacturer's instructions. Biotinylated cDNA were prepared according to the standard Affymetrix protocol. Probes were labeled, hybridized, and scanned according to the manufacturer's instructions. Data were extracted through Expression Console (Affymetrix, California). Data normalization and analysis were performed in GeneSpring GX software.

### 2.8. Bioinformatical Analysis of Data

To determine the significance of differentially expressed genes, we used a conservative level with Fisher's exact test (false discovery rate < 0.05) and fold-change ≥ 2. To determine the functional significance of differentially expressed genes, BLAST2GO was used to examine associations between GO and biological function. Intersection network analysis for gene sets derived from KEGG pathways (http://www.genome.jp/kegg/pathway.html). For joint analysis of miRNA and mRNA profiling data, we used miRNA target predictions based on TargetScan (Release 6.2, June 2012) (http://www.targetscan.org/) and miRanda (release August 2010) (http://www.microrna.org/). Integrated analysis of the inverse relations of expressed miRNAs and mRNAs in conjunction with target predictions was as described [19].

### 2.9. Immunofluorescence

Sca-1^+^CD31^−^ and Sca-1^+^CD31^+^ cells were analyzed with IF staining for surface markers, cardiomyocyte fate using Cardiac Troponin I, smooth muscle cell fate using *α*-Smooth Muscle Actin, and endothelial fate using Von Willebrand Factor. Induced and control group cells were labeled with IF for cardiac cell lineage-specific markers. Cells were washed with PBS and fixed with 4% paraformaldehyde for 15 min at room temperature (RT), permeabilized if noted with 0.1% Triton X-100 (Sigma) for 20 min at RT, washed with PBS, blocked with 10% goat serum (Multisciences Biotech) in PBS for 1 h at 37°C, and incubated for 2 hours at RT with primary antibodies against Cardiac Troponin I (Abcam), *α*-Smooth Muscle Actin (Abcam), or Von Willebrand Factor (Abcam). After rinsing with PBS, cells were incubated with DyLight® 488-conjugated goat anti-rabbit secondary antibodies (Abcam) for 1 h at RT. Nuclei were counterstained with 20 *μ*g/mL DAPI (Sigma) in PBS for 10 min at RT. Immunostaining was imaged with an inverted fluorescence microscope (Leica DMI3000B) and analyzed by Image-Pro Plus 7.0 software (Media Cybernetics). For controls, cells were stained without primary but with secondary antibody and analyzed in the relevant fluorescence channel to exclude autofluorescence and nonspecific secondary antibody binding.

### 2.10. Statistical Analysis

For miRNA and mRNA profiling by microarray, we used 40 mice as a pool. Statistical analyses for dd-PCR were performed with GraphPad Prism 6 software. Each experiment was repeated at least three times. Continuous variables were expressed as mean ± standard deviations. *P* < 0.05 was considered statistically significant.

## 3. Results

### 3.1. Isolation and* In Vitro* Culture of Mouse Heart-Derived Sca-1^+^ Cells

FACS is efficient for cell isolation and provides cell populations with 95% or higher purity [20]. We used FACS to isolate resident stem cells from mice hearts based on surface marker expression. After filtration through a 40 *μ*m cell strainer, whole heart cells were identified based on Lin and CD45 staining cells representing more than 98.0% of total heart cells. Lin^−^CD45^−^ cells were sorted for three subtypes: Sca-1^−^CD31^+^ (0.046 ± 0.027%), Sca-1^+^CD31^+^ (3.28 ± 0.30%), and Sca-1^+^CD31^−^ (0.735 ± 0.03%) (Table 1). Cells were largely negative for CD45 but widely expressed the stem cell antigen Sca-1, consistent with previous studies [13, 21]. To verify FACS efficacy, we performed FCM to analyze enrichment of Sca-1^+^, CD31^+^, and CD31^−^ cells in samples. Enriched population purity was more than 99% (data not shown).

**Figure 1:**
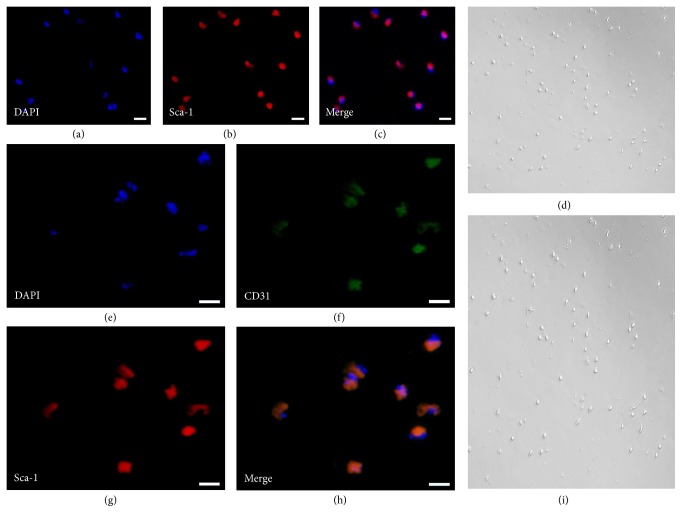
Analysis of CD31 and Sca-1 expression by IF staining. (a–c) Sca-1 expression in Sca-1^+^CD31^−^ cells with Sca-1 only in Sca-1^+^CD31^−^ cells. (d) Sca-1^+^CD31^−^ cell morphology using inverted microscopy and ordinary light. (e–h) Coexpression of CD31 and Sca-1 in Sca-1^+^CD31^+^ cells. (i) Sca-1^+^CD31^+^ cell morphology as above. Merging fluorescent signals showed no heterogeneous populations after FACS-based sorting. All cells were triple-stained for Sca-1 (red), CD31 (green), and DAPI (blue). Scale bars, 10 *μ*m.

When viewed with an inverted microscopy in ordinary light, cells in Sca-1^+^CD31^−^ and Sca-1^+^CD31^+^ populations were sparse (Figures 1(d) and 1(i)). Analysis of CD31 and Sca-1 expression in these cells by IF staining revealed coexpression of CD31 and Sca-1 in Sca-1^+^CD31^+^ cells and only Sca-1 in Sca-1^+^CD31^−^ cells (Figures 1(a)–1(c) and 1(e)–1(h)). We considered the efficiency and specificity of FACS to be reasonable.

### 3.2. *In Vitro* Differentiation of Sca-1^+^CD31^−^ and Sca-1^+^CD31^+^ Cells

**Figure 2:**
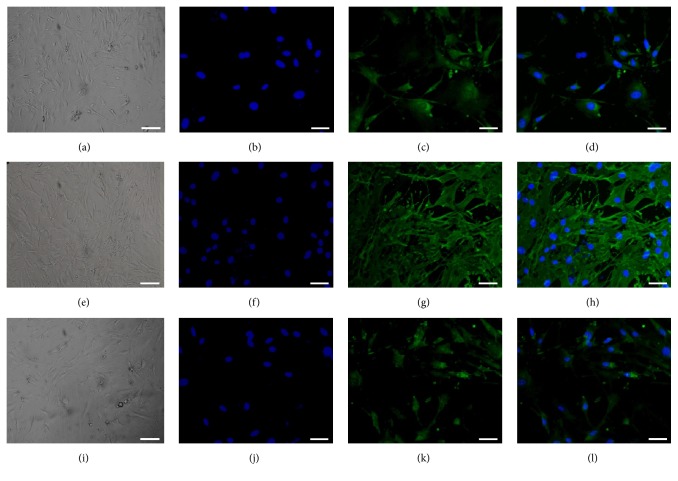
Differentiation potential of Sca-1^+^CD31^−^ cells into cardiomyocyte, smooth muscle, and endothelial cells* in vitro* using FACS. Cells were stained for specific markers after induction. Immunofluorescence staining (green) of cardiac-specific marker-Cardiac Troponin I (c), smooth muscle marker *α*-Smooth Muscle Actin (g), endothelial cell marker-Von Willebrand Factor (k). (a, e, i) Controls with bright light to show cell morphology after induction. (b, f, j) Nuclei (blue) counterstained with DAPI. (d, h, l) Merged images (b-c), (f-g), and (j-k). Scale bars, 50 *μ*m.

Differentiation is an important feature of stem and progenitor cells. To estimate the differentiation potential of cells into cardiomyocyte, smooth muscle, and endothelial lineages, Sca-1^+^CD31^−^ and Sca-1^+^CD31^+^ cells were cultured in specific differentiation medium and simultaneously in differentiation medium without growth factors as controls. After induction, lineage-specific markers were analyzed by IF. After 2 weeks of induction to a cardiomyocyte fate, cell morphology changed for Sca-1^+^CD31^−^ but not Sca-1^+^CD31^+^ cells (Figures 2(a) and S1-A; see Supplementary Material available online at http://dx.doi.org/10.1155/2016/9586751). Cardiac Troponin I-positive cells were detected in Sca-1^+^CD31^−^ but not Sca-1^+^CD31^+^ cells (Figures 2(c) and 2(d) and S1-CD). After 2 weeks of induction to smooth muscle cells, cell morphology changed for Sca-1^+^CD31^−^ but not Sca-1^+^CD31^+^ cells (Figures 2(e) and S1-E). We detected *α*-smooth muscle actin-positive cells in Sca-1^+^CD31^−^ but not Sca-1^+^CD31^+^ cells (Figures 2(g) and 2(h) and S1-GH). After 2 weeks of induction to an endothelial fate, cell morphology changed for Sca-1^+^CD31^−^ but not Sca-1^+^CD31^+^ cells (Figures 2(i) and S1-I). Von Willebrand Factor-positive cells were detected in Sca-1^+^CD31^−^ but not in Sca-1^+^CD31^+^ cells (Figures 2(k) and 2(l) and S1-KL). No Cardiac Troponin I-positive, *α*-smooth muscle actin-positive, and Von Willebrand Factor-positive cells were observed in control groups (data not shown).

Overall, these data indicated that mouse heart-derived Sca-1^+^CD31^−^ cells were multipotent and retained the ability to differentiate into cardiac cell lineages, but Sca-1^+^CD31^+^ cells did not.

### 3.3. miRNA Array Analysis

**Figure 3:**
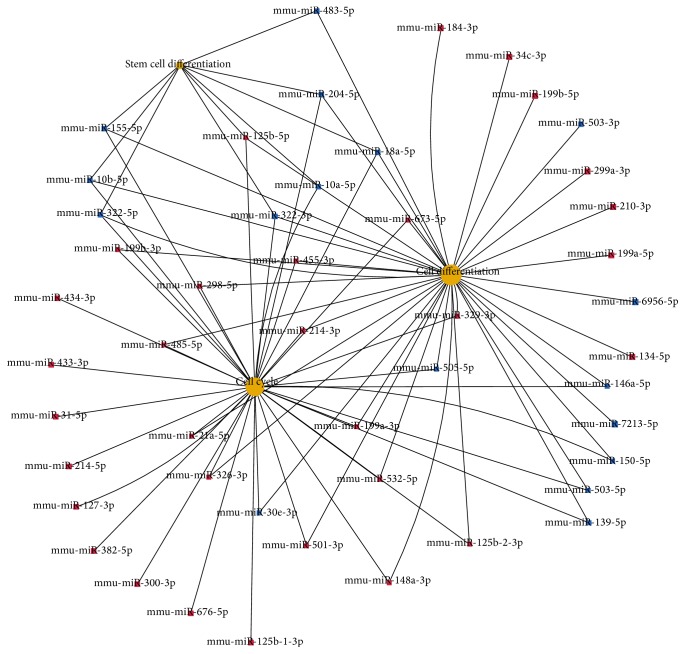
Differentially expressed miRNA and cell cycle and stem cell differentiation. Red: increased expression in Sca-1^+^CD31^−^ compared to Sca-1^+^CD31^+^ cell; blue: decreased expression.

Two samples from 40 Sca-1^+^CD31^−^ and Sca-1^+^CD31^+^ mouse heart cell populations were analyzed by FACS, 3 × 10^6^ cells per sample. RNA from the cells was analyzed with miRNA and mRNA arrays. MiRNAs with expression that increased or decreased with a false discovery rate < 0.05 and fold-change ≥ 2 are presented in Table S1. A total of 170 miRNAs were differentially expressed between Sca-1^+^CD31^−^ and Sca-1^+^CD31^+^ cells. The top 20 upregulated and downregulated miRNAs in Sca1^+^CD31^−^ cells compared to Sca1^+^CD31^+^ cells are in Table 2, (A)/(B). In these differentially expressed miRNAs, gene ontology and KEGG pathway analyses and signal transduction pathway association analysis showed targets associated with 179 pathways as significantly enriched (*P* < 0.05) (Table S2). Functions of the top 20 pathways were cancer, PI3K-Akt signaling, focal adhesion, regulation of the actin cytoskeleton, axon guidance, and MAPK signaling (Table S3). To determine the functional importance of differentially expressed miRNA targets, BLAST2GO was used to examine associations between GO and biological function. The top 20 GO terms included regulation of transcription DNA-dependence, positive regulation of transcription from RNA polymerase II promoters, positive regulation of transcription, DNA-dependence, cell differentiation, cell adhesion, positive regulation of cell proliferation, and negative regulation of apoptotic processes, with 101 targets in cell differentiation, 82 in cell adhesion, 65 in positive regulation of cell proliferation, and 72 in negative regulation of apoptotic process (Table S4). Mmu-miR-322-3p/5p, mmu-miR-155-5p, mmu-miR-204-5p, mmu-miR-10a-5p, and mmu-miR-125b-5p participated in cell differentiation, cell cycle, and stem cell differentiation (Figure 3).

**Figure 4:**
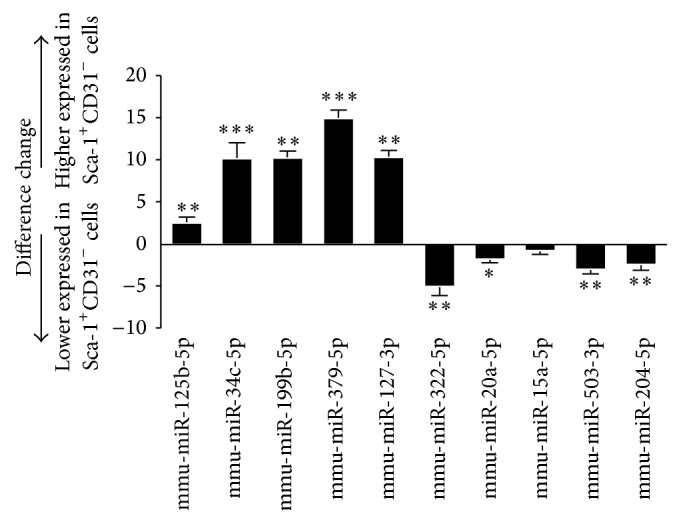
Expression profiles of miRNAs detected by dd-PCR. Bars: changes in miRNAs expression in Sca-1^+^CD31^−^ cells versus Sca-1^+^CD31^+^ cells. Expression was normalized to miRNA U6 and expression change was calculated based on the mean of three biological replicates. Bars above and below the *x*-axis: upregulated and downregulated miRNAs in Sca-1^+^CD31^−^ cells. Differences were analyzed with Student's *t*-test. ^*∗*^ *P* < 0.05, ^*∗∗*^ *P* < 0.01, and ^*∗∗∗*^ *P* < 0.001.

To validate the miRNA microarray results, 10 differentially expressed miRNAs were randomly chosen for analysis by dd-PCR. The miRNAs were mmu-miR-125b-5p, mmu-miR-34c-5p, mmu-miR-199b-5p, mmu-miR-379-5p, mmu-miR-127-3p, mmu-miR-322-5p, mmu-miR-20a-5p, mmu-miR-15a-5p, mmu-miR-503-3p, and mmu-miR-204-5p. Dd-PCR results confirmed the miRNA microarray results (Figure 4). Mmu-miR-125b-5p, mmu-miR-34c-5p, mmu-miR-199b-5p, mmu-miR-379-5p, and mmu-miR-127-3p were increased in expression in Sca1^+^CD31^−^ cells compared to Sca1^+^CD31^+^ cells. Mmu-miR-322-5p, mmu-miR-20a-5p, mmu-miR-15a-5p, mmu-miR-503-3p, and mmu-miR-204-5p were decreased in expression in Sca1^+^CD31^−^ cells.

### 3.4. mRNA Array Analysis

Expression profiling of undifferentiated cells confirmed that Sca1^+^CD31^−^ cells had a signature distinct from Sca1^+^CD31^+^ cells. A total of 1818 genes were differentially expressed between Sca-1^+^CD31^−^ and Sca-1^+^CD31^+^ cells (false discovery rate < 0.05 and fold-change ≥ 2, Table S5). Expressed genes did not contain any markers of mast cells (CD33), hematopoietic cells (CD45), or lineage (Lin). Some cardiac-specific transcription factors such as GATA4, GATA6, and cytokine transforming growth factor- (TGF-) *β*1 were increased in expression in Sca-1^+^CD31^−^ cells. Others such as MEF2c, and KDR, a progenitor endothelial cell marker had reduced expression in Sca-1^+^CD31^−^ cells. The *α*-sarcomeric actin protein, *α*-smooth muscle actin (a-SMA) was not expressed before differentiation. The top 20 mRNAs with increased or decreased expression in Sca-1^+^CD31^−^ compared to Sca-1^+^CD31^+^ cells are in Table 2, (C)/(D). We analyzed that enrichment of pathways associated the genes for differentially expressed mRNAs. We identified 73 pathways as significantly enriched (*P* < 0.05). Among these, the top 20 pathways involved PI3K-Akt signaling, ECM-receptor interaction, focal adhesion, protein digestion and absorption, complement and coagulation cascade, cytokine-cytokine receptor interaction, and cancer pathways (Table S6). To determine the functional significance of differentially expressed genes, BLAST2GO was used to examine associations between GO and biological function. The top 20 GO terms included cell adhesion, cell differentiation, cell migration, positive/negative regulation of cell proliferation, and negative regulation of apoptotic process (Table S7).

**Figure 5:**
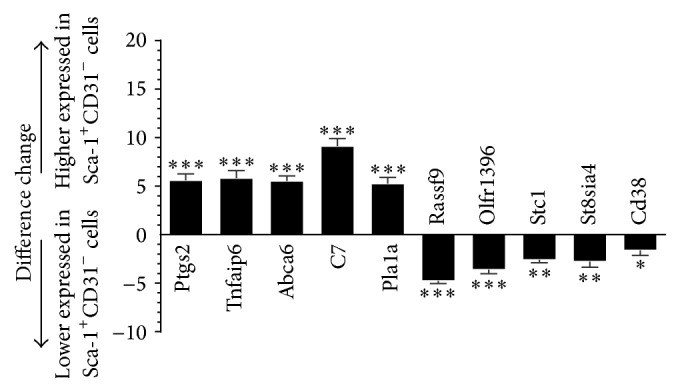
Expression profiles of genes detected by dd-PCR. Bars: difference changes in expression in Sca-1^+^CD31^−^ cells versus Sca-1^+^CD31^+^ cells. Expression was normalized to GAPDH and the expression change was calculated based on a mean of three biological replicates. Bars above and below the *x*-axis show upregulated and downregulated genes in Sca-1^+^CD31^−^ cells. Differences were analyzed with Student's *t*-test. ^*∗*^ *P* < 0.05, ^*∗∗*^ *P* < 0.01, and ^*∗∗∗*^ *P* < 0.001.

To validate the microarray data, 10 differentially expressed genes were randomly chosen for analysis by dd-PCR: Ptgs2, Tnfaip6, Abca6, C7, Pla1a, Rassf9, Olfr1396, Stc1, St8sia4, and Cd38. These genes were differentially expressed between Sca-1^+^CD31^−^ and Sca-1^+^CD31^+^ cells and dd-PCR results confirmed the mRNA microarray results (Figure 5).

### 3.5. Integrative Analysis of MicroRNA and mRNA Data

MiRNAs modulate gene expression by inducing mRNA degradation or translational repression, or both. Therefore, we performed an integrated analysis of miRNA and mRNA expression patterns. We analyzed enrichment of pathways based on the inversely associated miRNA targets for significantly differentially expressed miRNAs and mRNAs (false discovery rate < 0.05 and fold-change ≥ 2). We identified 29 pathways as significantly enriched (*P* < 0.05). Among these, miRNAs decreased with target genes increased in Sca-1^+^CD31^−^ cells compared to Sca-1^+^CD31^+^ cells and were involved in cancer, PI3K-Akt signaling, Rap1 signaling, mTOR signaling, protein digestion and absorption, complement and coagulation cascade, cytokine-cytokine receptor interaction, and cancer pathways (Figure 7(a)). The miRNAs that decreased with targets that were increased in Sca-1^+^CD31^−^ cells compared to Sca-1^+^CD31^+^ cells were involved in melanogenesis, Wnt signaling, and metabolic pathways (Figure 7(b)). We found 20 miRNAs and 49 mRNAs that were inversely associated, forming 50 relationship pairs (Table 3, Figure 8). We found that 13 miRNAs had decreased expression and 7 had increased expression in Sca-1^+^CD31^−^ cells compared to Sca-1^+^CD31^+^ cells. The interrelationships of pathways showed that, based on cocitation, some targets were highly networked. For example, stcl and Podxl were predicted to be targeted by several inversely regulated miRNAs including mmu-miR-125b-5p, mmu-miR-300-3p, mmu-miR-199a-5p, mmu-miR-199a-5p, mmu-miR-199b-5p, and mmu-miR-214b-5p (Figure 8). Integrated analysis indicated that five genes Dclk1, Slit2, Ctgf, Notch2, and Mgp had increased expression in Sca-1^+^CD31^−^ cells and participated in differentiation. Dclk1 was the target of mmu-miR-15a-5p, Slit2 was the target of mmu-miR-322-5p, Ctgf and Notch2 were targets of mmu-miR-18a-5p, and Mgp was the target of mmu-miR-155-5p. Mmu-miR-322-5p and mmu-miR-505-5p had more targeted and inversely associated genes and transcription factors (Table 3). Mmu-miR-322-5p had decreased regulation in Sca-1^+^CD31^−^ compared to Sca-1^+^CD31^+^ cells. Mmu-miR-322-5p was overrepresented for targets involved in cell division-associated pathways such as cyclins A2, B1, and D1, cyclin-dependent kinase, and cell division cycle 2. Analysis of the inversely associated interaction relationship pairs showed that mmu-miR-322-5p was decreased with targets that increased Adamts5, Adamtsl3, Arhgap20, Cacna2d1, Cdon, Dclk1, Fgfr1, Has2, Islr, Slit2, and Sobp; of these, Cacna2d1 was involved in the MAPK signaling and hypertrophic cardiomyopathy pathways and Fgfr1 was involved in p53, PI3K-Akt, and Ras signaling. Mmu-miR-505-5p was downregulated in Sca-1^+^CD31^−^ compared to Sca-1^+^CD31^+^ cells, with targets Cyp1b1, Dcbld2, Igf1, and Ppp1r14b,Txndc5 increased in expression; Igf1 was involved in mTOR and PI3K-Akt signaling and Cyp1b1by with microRNAs in cancer.

These results indicated that Sca-1^+^CD31^−^ cells were multipotent and retained the ability to differentiate into different cardiac cell lineages. However, Sca-1^+^CD31^+^ cells did not, partially due to differential expression of some microRNAs for regulation of genes, get involved in cell division, proliferation, antiapoptosis, or migration.

## 4. Discussion

Sca-1 is commonly used to identify stem cells in murine models and Sca-1-positive cells exhibit multipotential differentiation [22]. We tested the potential of Sca-1^+^CD31^−^ and Sca-1^+^CD31^+^CSC cells for cardiomyocyte, smooth muscle, and endothelial cell differentiation. We successfully isolated Sca-1^+^CD31^−^ and Sca-1^+^CD31^+^CSC cells from adult mice by FACS. Sca-1^+^CD31^−^ subgroups showed multipotential differentiation when cultured in the presence of BMP-2, FGF-4, PDGF-BB, and VEGF_165_ and induced to differentiate into cardiomyocyte, smooth muscle, or endothelial cells. Not all Sca-1-positive cells had multipotential differentiation ability, consistent with other studies [12]. Of more than 98% of Lin^−^CD45^−^ cells observed in cardiac tissue single cell suspension, all Sca-1^+^ cells lacked Lin and CD45 and most possessed CD31 (3.28%). Therefore, Lin^−^CD45^−^ seemed unnecessary for sorting of Sca-1^+^ cells. No contamination from fibroblasts or hematopoietic cells was found. Upon induction of differentiation, Sca-1^+^CD31^−^ CSCs became cardiomyocytes and smooth muscle and endothelial cells. We concluded that a simplified enzymatic dissociation method yielded 0.735% of Sca-1^+^CD31^−^ CSCs that could be obtained by FACS. Although several laboratories have published studies describing the isolation and characterization of mouse Sca-1^+^CD31^−^ and Sca-1^+^CD31^+^ CSCs [13], the stability of stem cells in the population, contamination from hematopoietic stem cells, and differentiation into cells of cardiovascular lineage has not been clearly established and therefore remains controversial.

Adult heart regeneration results from distinct CSC subtypes with self-renewal and multipotentiality-repopulating ability [23], but the molecular basis for differences between subtypes in stemness characteristics is largely unknown. A study using cardiac progenitor cell types including Isl-1/Nkx2.5 found that only Isl-1^+^/Nkx2.5^−^ cardiac progenitor cells showed significant cardiomyogenic differentiation, although the underlying reason is unclear [6]. Adult heart-derived Sca-1^+^/Isl-1^+^/Nkx2.5^−^ stem and progenitor cells are suggested to represent a true population of cardiac progenitor cells [24]. Sca-1-positive cells might have more subtypes with multipotential differentiation.

For determining the molecular basis for differences between subtypes in differentiation, analysis of mRNA and miRNA profiling data has increased the process specificity and quality of predicted relationships by statistically selecting miRNA target interactions [19]. We hypothesized that miRNA expression profiling would expand current knowledge about molecular regulation and enable identification of molecular mechanisms for stemness characteristics. Islet-1 (ISL-1), a marker of the secondary heart field [25], was not detectable in Sca-1^+^CD31^−^ and Sca-1^+^CD31^+^ CSC cells, and TBX5, a marker of the primary heart field [24], was detected, with expression of TBX5 higher in Sca-1^+^CD31^−^ than Sca-1^+^CD31^+^. The cardiac-specific transcription factors GATA-4, GATA-6, and MEF2C were all expressed in Sca-1^+^CD31^−^ and Sca-1^+^CD31^+^ CSC cells with higher expression of GATA-4 and lower expression of MEF2C in Sca-1^+^CD31^−^ compared to Sca-1^+^CD31^+^ cells. Sca-1^+^CD31^+^ cells are a source of cardiac endothelial cell progenitors in the adult mouse heart [14]. Microarray data suggested that some endothelial cell-associated genes including ABCG2, VEGFRa, VWF, Cxcr4, Ecscr, Esam, and Esm1 had increased expression in Sca-1^+^CD31^+^ cells compared to Sca-1^+^CD31^−^ cells. Expression of ABCG2 was detected only in freshly isolated Sca-1^+^CD31^+^ cells.

**Figure 6:**
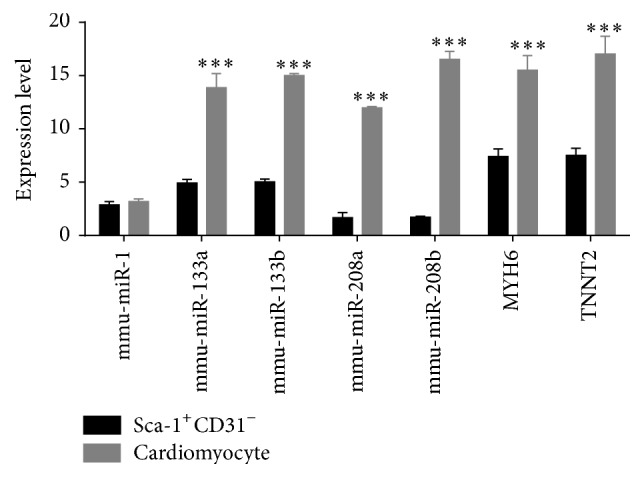
Expression of cardiomyocyte-specific miRNAs and mRNAs by dd-PCR. Bars: expression of genes in freshly isolated Sca-1^+^CD31^−^ cells (black) or differentiated cardiomyocytes (gray). Expression was normalized to miRNA U6 and GAPDH and expression was calculated based on a mean of three biological replicates. Differences were analyzed with Student's *t*-test. ^*∗∗∗*^ *P* < 0.001.

**Figure 7:**
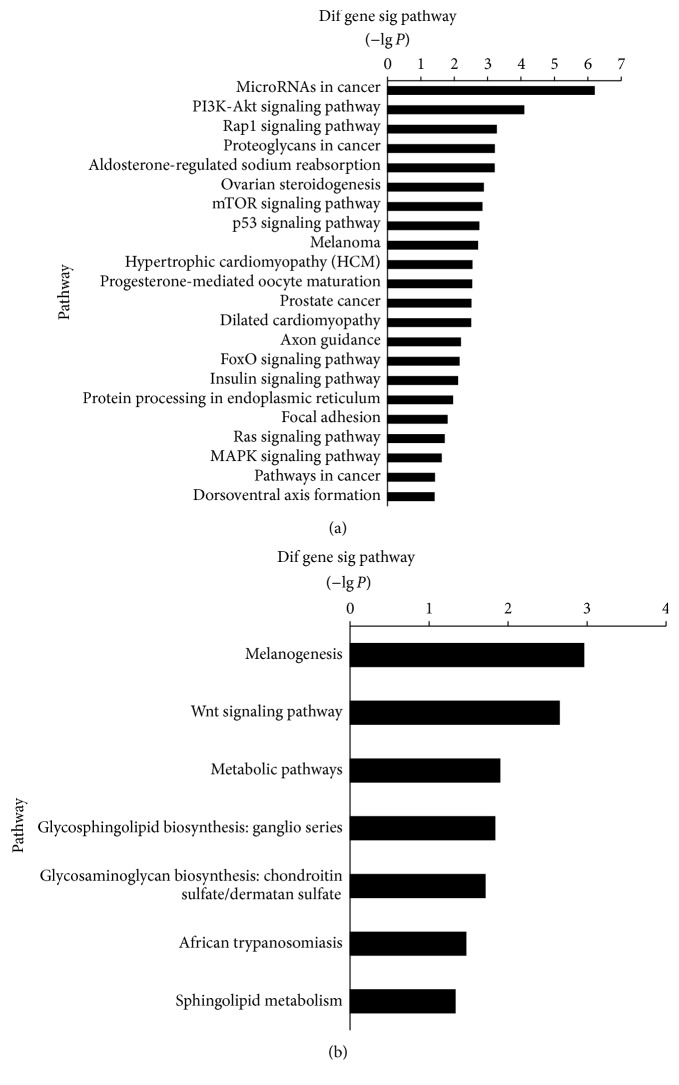
Pathway enrichment from integrated analysis of differentially expressed miRNAs and mRNAs in conjunction with target predictions. (a) Pathway enrichment for miRNAs downregulated with targets in upregulated genes in Sca-1^+^CD31^−^ cells compared to Sca-1^+^CD31^+^ cells. (b) Pathway enrichment for miRNAs upregulated with targets in downregulated genes in Sca-1^+^CD31^−^ cells compared to Sca-1^+^CD31^+^ cells.

**Figure 8:**
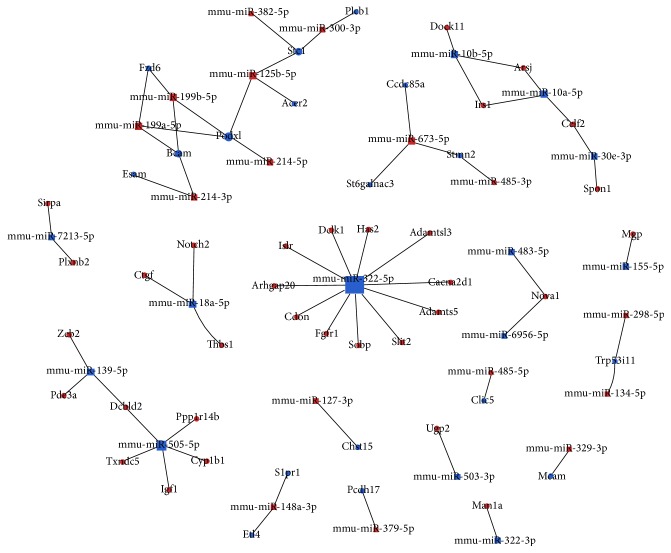
Integrated analysis of the inverse relationship of differentially expressed miRNAs and mRNAs in conjunction with target predictions. Blue box: miRNAs with reduced expression in Sca-1^+^CD31^−^ compared to Sca-1^+^CD31^+^ cells. Blue points: mRNAs with reduced expression in Sca-1^+^CD31^−^ compared to Sca-1^+^CD31^+^ cells. Red box: miRNAs with increased expression in Sca-1^+^CD31^−^ compared to Sca-1^+^CD31^+^ cells. Red points: mRNAs with increased expression in Sca-1^+^CD31^−^ compared to Sca-1^+^CD31^+^ cells. Block area indicates number of targeted and inversely associated genes.

Among miRNAs expressed in the heart, miR-1, miR-133, miR-208, and miR-499 are the most commonly investigated subtypes. Both are involved in regulating the proliferation and differentiation of human CPCs [17]. Recent studies provide evidence for the influence of other, less common miRNAs subtypes such as miR-204, miR-669a, miR-669q, miR-23a, and miR-23b in promoting CPC differentiation. Results from these studies show that the function of miRNAs in CSCs and CPCs largely depends on the nature of their molecular targets [17]. In integrated analysis of the inverse relationship of expressed miRNAs and mRNAs, mmu-miR-204 expression was reduced in Sca-1^+^CD31^−^ compared to Sca-1^+^CD31^+^ cells, using target musculus collagen, type VIII, alpha 1, and musculus enhancer trap locus 4. Reduced levels of miR-1 and miR-133 are observed in mouse ESCs following artificial induction of myocardial differentiation [26]. In our study, mmu-miR-1 was not differently expressed and mmu-miR-133 and mmu-miR-208a expression was reduced in Sca-1^+^CD31^−^ compared to Sca-1^+^CD31^+^ cells. Analysis of the expression of cardiomyocyte-specific miRNAs miR-1, miR-133a/b, and miR-208a/b and mRNAs* MYH6* and* TNNT2* showed upregulation of miR-1, miR-133 a/b, miR-208a/b, and* MYH6* and* TNNT2* in differentiated cardiomyocyte cells compared to freshly isolated Sca-1^+^CD31^−^ cells (Figure 6). This analysis revealed a miRNA pattern indicative of stem cell characteristics. In addition, we corroborated the expression in Sca-1^+^CD31^−^ cells of differentiation promoting-miRNAs mmu-miR-322-5p, mmu-miR-505-5p, mmu-miR-18a-5p, and mmu-miR-139-5p. These miRNAs were among the miRNAs with the highest number of inversely associated targets and transcription factors and the highest degree of cooperative target regulation. Mmu-miR-322-5p was downregulated in Sca-1^+^CD31^−^ cells compared to Sca-1^+^CD31^+^ cells with overrepresentation of targets involved in cell division-associated pathways such as cyclins A2, B1, and D1, cyclin-dependent kinase, and cell division cycle 2. MiR-322-5p is reported to be induced during myogenesis and to promote cdk2 inhibition by downregulating Cdc25A, the phosphatase responsible for removing inhibitory phosphorylation of cdk2 [27]. We corroborated inverse association of Adamts5, Adamtsl3, Cdon, Dclk1, Fgfr1, Has2, Islr, Slit2, Sobp, and Arhgap20 with miR-322-5p. We showed that Cdon was associated with cyclin signaling, the cell division cycle, and negative regulation of the mitotic cell cycle, consistent with studies in other cell types that found that Cdon promotes myogenic differentiation via activation of p38MAPK [28]. Dclk1 was associated with cell differentiation and multicellular organism development. Dclk1 is marker of quiescent and cycling stem cells [29]. Fgfr1 participated in PI3K-Akt and MAPK signaling. In other studies, Fgfr1 is associated with mesenchymal cell differentiation. Genetic studies placed the Fgfr1 gene at the top of major ontogenic pathways that enable gastrulation, tissue development, and organogenesis [30]. Fgfr1, alone and with its partner nuclear receptors RXR and Nur77, targets thousands of active genes and controls expression of homeoboxes and neuronal and mesodermal genes and in pluripotency, ensuring differentiation of pluripotent embryonic stem cells [30]. Slit2 was associated with negative regulation of the cellular response to growth factor stimulus and smooth muscle cell chemotaxis. Some reports found that Slit2 inhibits human fibrocyte differentiation [31]. This finding indicates that miR-322-5p participates in the regulation of the cell cycle, cell growth, and differentiation. Studies have confirmed targets of miR-322-5p in myoblast differentiation including Chk1, Wee1, cyclin D1, and cyclin E1 in other tissues and cell types [32].

### 4.1. Concluding Remarks

A number of miRNA subtypes have been investigated for their role in regulating the differentiation and proliferation of CSCs. However, many aspects about CSCs remain to be fully understood. In particular, the molecular basis for differences between subtypes in stemness characteristics is largely unknown. Our results indicated that mouse heart-derived Sca-1^+^CD31^−^ cells were multipotent and retained the ability to differentiate into cardiac cell lineages, but Sca-1^+^CD31^+^ cells did not. Analysis of the expression of cardiomyocyte-specific miRNAs miR-1, miR-133a/b, and miR-208a/b and mRNAs* MYH6* and* TNNT2* revealed miRNA patterns indicative of stem cell characteristics. Integrated analysis of miRNA and mRNA expression patterns indicated that 20 miRNAs and 49 mRNAs were inversely associated with Sca-1^+^CD31^−^ and Sca-1^+^CD31^+^ subtypes in stemness characteristics. These results revealed a differentiation miRNA network and identified putative mRNAs targeted by multiple miRNAs. Mmu-miR-322-5p had more targeted and inversely associated genes and transcription factors and might have higher potential for involvement in CSC/CPCs differentiation. These data suggested a miRNA network in mouse heart CSC Sca-1^+^ subtypes and supported the hypothesis that miRNA networks reenforce transcriptional control during CSC/CPC differentiation. Therefore, future studies focusing on determining these miRNA functions in Sca-1^+^CD31^−^ and Sca-1^+^CD31^+^ cells will provide novel insights into the regulation mechanism of miRNAs in CSCs. The findings should eventually result in the development of new therapeutic modalities.

## Supplementary Material

Figure S1. Differentiation potential of Sca-1+CD31+cells by FACS into cardiomyocyte, smooth muscle, and endothelial cells in vitro.
Table S1. The differently expressed miRNAs between Sca1+CD31−and Sca1+CD31+ cells.
Table S2. KEGG pathway analyses of predicted miRNA targets.
Table S3. Pathway of the differently expressed miRNAs (the top 20 pathway) and their corresponding target genes.
Table S4. Cluster of the differently expressed miRNAs (the top 20 GO terms) and their corresponding target genes.
Table S5. The differently expressed mRNAs between Sca1+CD31−and Sca1+CD31+ cells.
Table S6. Pathway of the differently expressed mRNAs (the top 20 pathway) and their corresponding genes.
Table S7. Cluster of the differently expressed mRNAs (the top 20 GO terms) and their corresponding genes.

## Figures and Tables

**Table 1:** Surface markers expressed on the whole adult mouse heart cells.

Surface markers	Cells (%)
Sca1^+^CD31^+^	3.28 ± 0.30%
Sca1^+^CD31^−^	0.735 ± 0.03%
Sca1^−^CD31^+^	0.046 ± 0.027%
CD45^+^	≤1

*N* = 20.

**Table 2:** Differentially expressed miRNAs and mRNAs.

Transcript ID	Fold change	Gene symbol	Fold change
Sca1^+^CD31^−^ versus Sca1^+^CD31^+^		Sca1^+^CD31^−^ versus Sca1^+^CD31^+^	
(A) Top 20 upregulated miRNAs		(C) Top 20 up-expressed genes	

mmu-miR-379-5p	90.24449	Ptgs2	43.16952
mmu-miR-199b-5p	87.55872	Tnfaip6	38.89182
mmu-miR-127-3p	54.19857	C7	37.97997
mmu-miR-541-5p	45.80033	Abca6	30.55509
mmu-miR-411-5p	33.4714	Gfpt2	28.29249
mmu-miR-214-5p	28.61253	Abca9	27.74439
mmu-miR-214-3p	25.16356	C3	25.98105
mmu-miR-337-5p	24.94587	Kcnt2	25.8089
mmu-miR-199a-3p	23.92178	Efemp1	25.56114
mmu-miR-199b-3p	23.92178	Mfap5	24.76703
mmu-miR-382-5p	23.10211	Col14a1	24.13785
mmu-miR-125b-1-3p	22.55209	Lama2	23.00453
mmu-miR-199a-5p	22.46078	Olfml1	22.19862
mmu-miR-300-3p	21.63719	Pi15	22.06034
mmu-miR-455-3p	18.46332	Gpr133	21.53499
mmu-miR-134-5p	16.20999	Pla1a	21.27796
mmu-miR-433-3p	15.29315	Sema3c	21.14873
mmu-miR-210-3p	15.09217	Omd	20.96994
mmu-miR-154-5p	14.1989	Scn7a	20.86027
mmu-miR-329-3p	13.94587	Serpina3n	20.8287

(B) Top 20 downregulated miRNAs		(D) Top 20 down-expressed genes	

mmu-miR-503-3p	−13.639	Gm11037	−10.2032
mmu-miR-204-5p	−11.7122	Rassf9	−8.11733
mmu-miR-322-5p	−8.52674	Olfr1396	−7.99576
mmu-miR-30e-3p	−7.46444	Pcdh17	−7.72504
mmu-miR-139-3p	−6.94	Klra9	−7.66729
mmu-miR-503-5p	−6.09334	Stc1	−6.4747
mmu-miR-10a-5p	−5.99097	Rtp3	−6.31498
mmu-miR-150-5p	−5.38081	St8sia4	−6.24289
mmu-miR-155-5p	−5.29758	Fabp9	−6.20996
mmu-miR-10b-5p	−4.92881	Il2rg	−6.20395
mmu-miR-139-5p	−4.83405	Ifi44	−6.17352
mmu-miR-542-5p	−4.73842	Klhl4	−6.10709
mmu-miR-7213-5p	−4.59976	St8sia6	−6.10408
mmu-miR-146a-5p	−4.55355	Mmrn2	−6.03966
mmu-miR-322-3p	−4.54915	Cd38	−5.97605
mmu-miR-6956-5p	−4.33521	Pydc3	−5.96584
mmu-miR-505-5p	−4.3352	Ctla2b	−5.87224
mmu-miR-18a-5p	−4.21474	Rbp7	−5.84414
mmu-miR-483-5p	−4.03019	Cxcr4	−5.84201
mmu-miR-193a-3p	−3.8903	Rasgrf2	−5.80171

(A) Top 20 upregulated miRNAs and (B) top 20 downregulated miRNAs in Sca-1^+^CD31^−^ versus Sca-1^+^CD31^+^ cells.

(C) Top 20 upregulated mRNAs and (D) top 20 downregulated mRNAs in Sca-1^+^CD31^−^ versus Sca-1^+^CD31^+^ cells.

All genes had FDR < 0.05.

**Table 3:** The miRNA-mRNA regulation pairs.

Regulation in resistant cells	miRNA	Target genes
miRNA down-target up	mmu-miR-10b-5p	Arsj, Dock11, Irs1
	mmu-miR-155-5p	Mgp
	mmu-miR-18a-5p	Ctgf, Notch2, Thbs1
	mmu-miR-30e-3p	Celf2, Spon1
	mmu-miR-10a-5p	Arsj, Celf2, Irs1
	mmu-miR-139-5p	Dcbld2, Zeb2, Pde3a
	mmu-miR-322-3p	Man1a
	mmu-miR-322-5p	Adamts5, Adamtsl3, Arhgap20, Cacna2d1, Cdon, Dclk1, Fgfr1, Has2, Islr, Slit2, Sobp
	mmu-miR-483-5p	Nova1
	mmu-miR-503-3p	Ugp2
	mmu-miR-505-5p	Cyp1b1, Dcbld2, Igf1, Ppp1r14b, Txndc5
	mmu-miR-6956-5p	Nova1
	mmu-miR-7213-5p	Plxnb2, Sirpa

miRNA up-target down	mmu-miR-125b-5p	Acer2, Podxl, Stc1
	mmu-miR-127-3p	Chst15
	mmu-miR-134-5p	Trp53i11
	mmu-miR-148a-3p	Etl4, S1pr1
	mmu-miR-199a-5p	Bcam, Fzd6, Podxl
	mmu-miR-298-5p	Trp53i11
	mmu-miR-300-3p	Plcb1, Stc1
