# Supplementary material for: Differentiation-Associated MicroRNA Alterations in Mouse Heart-Derived Sca-1+CD31− and Sca-1+CD31+ Cells
Source: Stem Cells Int. 2016 May 19;2016:9586751. doi: 10.1155/2016/9586751 (PMC4889861; doi:10.1155/2016/9586751)
Supplement: Supplementary file 1 — Figure S1. Differentiation potential of Sca-1+CD31+cells by FACS into cardiomyocyte, smooth muscle, and endothelial cells in vitro. Table S1. The differently expressed miRNAs between Sca1+CD31−and Sca1+CD31+ cells. Table S2. KEGG pathway analyses of predicted miRNA targets. Table S3. Pathway of the differently expressed miRNAs (the top 20 pathway) and their corresponding target genes. Table S4. Cluster of the differently expressed miRNAs (the top 20 GO terms) and their corresponding target genes. Table S5. The differently expressed mRNAs between Sca1+CD31−and Sca1+CD31+ cells. Table S6. Pathway of the differently expressed mRNAs (the top 20 pathway) and their corresponding genes. Table S7. Cluster of the differently expressed mRNAs (the top 20 GO terms) and their corresponding genes. [file 9586751.f1.zip › Figure S1.pdf]

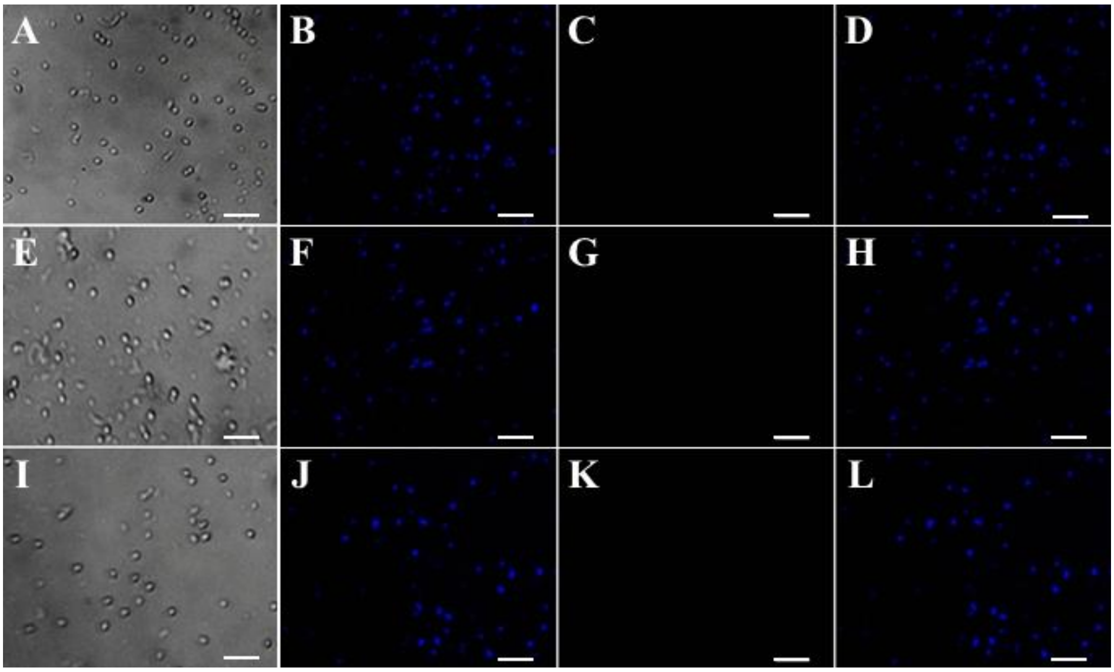

Figure S1.

Differentiation potential of Sca-1^{+}CD31^{+}cells by FACS into cardiomyocyte, smooth muscle, and endothelial cells in vitro. No cells were stained for the specific marker after induction.

Immuno-fluorescence staining of cardiac-specific marker-Cardiac Troponin I (C), smooth muscle marker α-Smooth Muscle Actin (G), enodthelial cell marker-Von Willebrand Factor (K). They were green and no cells were stained for green.

(A), (E) and (I) were controls with bright light to show cell morphology after induction, the cells morphology has not changed after induction.

(B), (F) and (J) Nuclei (blue) were counter-stained by DAPI.

(D), (H) and (L) were merged images of B, C, and F, G, and J, K, respectively. (Scale bars, 50 μm).
