# Supplementary material for: Differentiation-Associated MicroRNA Alterations in Mouse Heart-Derived Sca-1+CD31− and Sca-1+CD31+ Cells
Source: Stem Cells Int. 2016 May 19;2016:9586751. doi: 10.1155/2016/9586751 (PMC4889861; doi:10.1155/2016/9586751)
Supplement: Supplementary file 1 — Figure S1. Differentiation potential of Sca-1+CD31+cells by FACS into cardiomyocyte, smooth muscle, and endothelial cells in vitro. Table S1. The differently expressed miRNAs between Sca1+CD31−and Sca1+CD31+ cells. Table S2. KEGG pathway analyses of predicted miRNA targets. Table S3. Pathway of the differently expressed miRNAs (the top 20 pathway) and their corresponding target genes. Table S4. Cluster of the differently expressed miRNAs (the top 20 GO terms) and their corresponding target genes. Table S5. The differently expressed mRNAs between Sca1+CD31−and Sca1+CD31+ cells. Table S6. Pathway of the differently expressed mRNAs (the top 20 pathway) and their corresponding genes. Table S7. Cluster of the differently expressed mRNAs (the top 20 GO terms) and their corresponding genes. [file 9586751.f1.zip › Table S1.pdf]

Pathway ID	Pathway Name	Diff Gene Counts in Pathway	ene Amount in Pathw
4151	PI3K-Akt signaling pathway	73	356
5200	Pathways in cancer	69	326
5205	Proteoglycans in cancer	57	230
4360	Axon guidance	43	133
5166	HTLV-I infection	57	290
5161	Hepatitis B	42	149
4010	MAPK signaling pathway	53	259
4510	Focal adhesion	46	205
4810	Regulation of actin cytoskeleton	46	217
4722	Neurotrophin signaling pathway	34	123
4310	Wnt signaling pathway	35	148
4910	Insulin signaling pathway	34	141
4012	ErbB signaling pathway	27	87
5220	Chronic myeloid leukemia	25	74
5223	Non-small cell lung cancer	22	55
4916	Melanogenesis	28	100
5218	Melanoma	24	73
5215	Prostate cancer	26	89
4141	Protein processing in endoplasmic reticulum	34	169
5202	Transcriptional misregulation in cancer	35	181

richment Sco	p-value	FDR
5.9557588	9.89E-35	2.30E-32
6.1474602	8.55E-34	9.96E-32
7.1979903	5.86E-32	4.55E-30
9.3903343	1.28E-29	7.48E-28
5.7087509	2.33E-26	1.08E-24
8.1870465	2.87E-26	1.12E-24
5.9434737	1.82E-25	6.05E-24
6.5173074	4.92E-24	1.43E-22
6.1569033	6.254E-23	1.62E-21
8.0285671	3.49E-21	8.13E-20
6.868637	2.355E-19	4.99E-18
7.0036437	4.024E-19	7.81E-18
9.0138173	1.673E-18	3.00E-17
9.8123386	3.155E-18	5.25E-17
11.617809	5.068E-18	7.87E-17
8.1324662	8.181E-18	1.19E-16
9.548884	3.138E-17	4.30E-16
8.4849166	3.962E-17	5.13E-16
5.8432767	1.62E-16	1.99E-15
5.6163441	2.113E-16	2.45E-15

<table>
<tr><th>Gene Symbols</th></tr>
<tr><td>|Ifnar1|Ywhaq|Creb3l4|Itgb8|Itga11|Ywhaz|Ikbkb|Gnb4|Ppp2r1b|Igf1|Pik3cb|Ppp2r5a|Fgf20|Fgf16|Kdr|Rheb|Ppp2r5c|Ppp2r5d|Fgf7|Cl</td></tr>
<tr><td>|Fasl|E2f2|Fgf7|Smo|Crkl|Rela|Mapk8|Mapk1|Akt3|Fgf14|Rarb|Traf1|Mapk3|Cebpa|Pten|Zbtb16|Sos1|Stat5b|Fgf1|Map2k1|Lamc2|Kit|</td></tr>
<tr><td>|Prkacb|Wnt9a|Arhgef1|Thbs1|Hbegf|Fgf16|Prkcb|Fgf7|Kdr|Gab1|Met|Kras|Pik3r1|Fzd3|Pik3cd|Hoxa10|Rock1|Pik3ca|Igf1|Tgfb2|Sdc</td></tr>
<tr><td>|Plxnc1|Cfl2|Slit2|Nrp1|Mapk1|Srgap3|Cdk5|Rgs3|Pak2|Mapk3|Nfatc3|Arhgef12|Ntn1|Pak4|Plxna2|Plxna4|Sema3f|Sema3b|Sema4d|</td></tr>
<tr><td>|Wnt5a|Cdc27|Tgfb2|Akt2|Wnt8a|Atf2|Nfatc3|Wnt3|Pik3ca|Wnt9a|Wnt7b|E2f2|Vac14|Pik3cd|Bax|Nfatc1|Stat5b|Bcl2l1|Fzd5|Map2k4|</td></tr>
<tr><td>|Bax|Ccna2|Cdkn1a|Nfat5|E2f2|Atf2|Ccnd1|Stat5b|Elk1|Ifnar1|Tgfb2|Cdkn1b|Bcl2|Rela|Ywhaz|Fos|Egr2|Ywhaq|Akt2|Fasl|Pik3cd|Cre</td></tr>
<tr><td>|Mef2c|Cacna2d1|Prkcb|Fasl|Kras|Prkacb|Mknk2|Tgfb2|Mapk8ip2|Nras|Ppp3r1|Sos1|Dusp2|Cacna2d2|Mapk8|Stk4|Mapk3|Taok2|Pp</td></tr>
<tr><td>|Mapk1|Mapk8|Itgb1|Rap1b|Itga8|Elk1|Crkl|Map2k1|Mapk3|Rock1|Zyx|Tnc|Itga7|Akt3|Col5a1|Ccnd1|Mylk4|Kdr|Itgb8|Parva|Mylk|Pik3</td></tr>
<tr><td>|Itga7|Wasf2|Pak2|Fgf7|Fgfr3|Itga8|Mylk4|Itga11|Sos1|Diap2|Fgf16|Itga10|Fgf1|Fgf14|Pik3ca|Itgb1|Pik3cd|Itgb8|Itga1|Fgd3|Gna13|Fg</td></tr>
<tr><td>|Fasl|Akt2|Kras|Map2k1|Bcl2|Mapk1|Braf|Camk2g|Bax|Rela|Gab1|Irak3|Ngfr|Irs1|Map3k3|Plcg2|Mapk3|Sort1|Pik3r1|Mapk8|Rap1a|B</td></tr>
<tr><td>|Wnt9a|Wnt5a|Cxxc4|Prkacb|Wnt3|Wnt7b|Senp2|Mapk8|Wnt2b|Ppp3r1|Prkcb|Nfatc3|Ccnd1|Frat2|Daam1|Sfrp1|Nfat5|Nfatc4|Plcb1|</td></tr>
<tr><td>|Nras|Ptpn1|Irs1|Ppp1r3b|Slc2a4|Rheb|Ptprf|Mapk3|Mapk1|Map2k1|Pde3a|Ikbkb|Pik3cb|Tsc1|Pygm|Pklr|Sos1|Foxo1|Mapk8|Mknk2|</td></tr>
<tr><td>|Mapk3|Mapk8|Hbegf|Prkcb|Pik3cb|Mapk1|Gab1|Crkl|Pik3cd|Camk2g|Map2k4|Pik3r1|Map2k1|Pik3ca|Akt2|Akt3|Kras|Pak2|Cdkn1b|E</td></tr>
<tr><td>|Pik3ca|Nras|Pik3r1|Pik3cd|Gab2|Sos1|Map2k1|Mapk1|Crkl|Bcl2l1|Braf|Ikbkb|Kras|E2f3|Mapk3|Akt2|Akt3|Cdkn1b|E2f2|Rela|Pik3cb|</td></tr>
<tr><td>|Pik3ca|Rassf5|Akt2|Rassf1|Nras|Kras|Ccnd1|Pik3cb|Pik3cd|Stk4|Mapk3|Braf|Rarb|Akt3|Pik3r1|E2f3|Map2k1|Sos1|Prkcb|Mapk1|Plc</td></tr>
<tr><td>|Fzd3|Camk2g|Wnt3|Wnt5a|Creb1|Nras|Fzd4|Prkcb|Plcb1|Mapk3|Wnt2b|Wnt8a|Kras|Prkacb|Gnai2|Wnt9a|Fzd9|Gnai3|Kit|Gnao1|Gn</td></tr>
<tr><td>|E2f2|Mapk1|Pik3ca|Nras|Fgf14|Akt3|Map2k1|Braf|Pik3r1|Fgf1|Pten|Ccnd1|Igf1|Cdkn1a|Akt2|Fgf20|Kras|Met|Fgf16|Pik3cb|Fgf7|Map</td></tr>
<tr><td>|Ccnd1|Bcl2|Sos1|Pten|Pik3cd|Cdkn1a|Rela|Kras|Igf1|Pik3r1|Ikbkb|Foxo1|E2f3|Pik3cb|Nras|E2f2|Creb1|Creb3l4|Pik3ca|Mapk3|Map2</td></tr>
<tr><td>|Sec24d|Bax|Sec62|Pdia3|Man1a|Sec63|Ubqln4|Bcl2|Ganab|Edem3|Ube2e2|Sil1|Plaa|Bak1|Eif2s1|Dnajc5|Atxn3|Ube2g1|Ube4b|Der</td></tr>
<tr><td>|Whsc1|Mef2c|Met|Etv6|Foxo1|Ccnt2|Hoxa10|Eya1|Cebpb|Kdm6a|Aff1|Atm|Cdkn1a|Cdkn1b|Hhex|Flt1|Bmp2k|Sp1|Traf1|Zbtb16|Igf1|</td></tr>
</table>

had|Chrm1|Col5a1|Irs1|Ppp2r3a|Itga7|Rela|Itgb1|Itga8|Map2k1|Mapk1|Fgf1|Flt1|Lpar2|Mlst8|Il2ra|Tsc1|Ppp2r2a|Pi

|Wnt9a|Tgfb2|Bcl2l1|Cdkn1b|Wnt2b|Wnt5a|Prkcb|E2f3|Plcg2|Braf|Msh6|Pik3cb|Akt2|Nras|Bcl2|Pax8|Runx1t1|Ras

1|Fgf14|Smo|Arhgef12|Fasl|Pdcd4|Fgf20|Fzd6|Fzd9|Hoxd10|Sos1|Ank1|Wnt8a|Itgb1|Elk1|Fgf1|Frs2|Mapk1|Nras|

Kras|Unc5a|Limk2|Nfatc1|Met|Nras|Ntng1|Nfat5|Epha8|Nfatc4|Plxnb2|Sema3c|Sema3a|Itgb1|Ppp3r1|Gnai2|Efnb3

Fzd3|Wnt2b|Nrp1|Nras|Zfp36|Prkacb|Ets1|Atm|Srf|Kras|Fzd6|Nfatc4|Pold3|Trp53inp1|Nfyb|Akt3|Map3k3|Pik3r1|E

b1|Pik3r1|E2f3|Map2k1|Pten|Nfatc3|Nras|Cycs|Creb3l4|Map2k4|Mapk1|Nfatc1|Mapk8|Pik3cb|Nfatc4|Pik3ca|Mapk

m1a|Fgf14|Bdnf|Dusp16|Fgf16|Ikbkb|Map3k11|Rap1a|Srf|Map3k2|Fgf1|Fgf7|Rap1b|Cdc25b|Fos|Atf2|Ptprr|Fgf20|

cb|Pik3ca|Pak2|Sos1|Itga10|Igf1|Chad|Itga11|Pgf|Pak4|Akt2|Pik3cd|Bcl2|Thbs1|Flt1|Ccnd2|Parvb|Rap1a|Met|Lam

d1|Chrm1|Fgf20|Pik3cb|Arhgef1|Mapk1|Nras|Mapk3|Tmsb4x|Pik3r1|Crkl|Abi2|Limk2|Wasl|Map2k1|Arpc5l|Rock1|

dnf|Ikbkb|Crkl|Pik3ca|Akt3|Pik3cb|Camk4|Rps6ka5|Sos1|Pik3cd|Frs2|Nras|Rap1b|

Vnt8a|Nkd1|Btrc|Siah1b|Camk2g|Rock1|Siah1a|Csnk2a1|Nfatc1|Lrp6|Fzd6|Ccnd2|Fzd4|Fzd9|Fzd3|Fzd5|

Elk1|Prkar2a|Ppargc1a|Pik3cd|Kras|Akt2|Irs4|Crkl|Akt3|Trip10|Prkacb|Pik3ca|Braf|Pik3r1|

lk1|Stat5b|Braf|Sos1|Nras|Pak4|Cdkn1a|Plcg2|

Ccnd1|Tgfb2|Cdkn1a|Stat5b|

g2|E2f2|

aq|Creb3l4|Fzd5|Adcy1|Map2k1|Mapk1|Fzd6|Wnt7b|

k3|E2f3|Pik3cd|

k1|Akt3|Braf|Mapk1|Cdkn1b|Akt2|

l3|Hspa4l|Sar1a|Derl2|Ssr1|Sec24c|Rpn1|Mapk8|Ube2e3|Dnajc3|Rpn2|Txndc5|Man1b1|Dnajb1|Rnf185|

|Bcl6|Pax8|Ngfr|Runx1t1|Etv1|Ccnd2|Bcl2l1|Dot1l|Cebpa|Pbx1|Runx2|Slc45a3|Rela|Mycn|

ik3ca|Nras|Sos1|Pik3r1|Csf1|Itga10|Mapk3|Bcl2l11|Fgfr3|Ywhag|Cdkn1a|Tnc|Ngfr|Pgf|Kit|Pten|Creb1|Pik3cd|Fasl|Itga

sf5|Wnt3|Cycs|Pik3cd|Foxo1|Fzd6|Stk4|Fzd9|Fgf16|Wnt8a|Met|Kras|Wnt7b|Pgf|Rala|Pik3r1|Fzd4|Igf1|Itgb1|Fzd5|Ccn

zd5|Timp3|Mapk3|Map2k1|Wnt3|Akt2|Pik3cb|Braf|Akt3|Plcg2|Wnt2b|Ccnd1|Fzd4|Camk2g|Wnt7b|Wnt5a|Cdkn1a|Ank

|Ephb2|Cxcl12|Rock1|Robo1|Epha7|Epha5|Gnai3|

2f3|Fos|Ikbkb|Fzd9|Il2ra|Adcy1|Nfat5|Fzd4|Elk1|Mapk8|Creb1|Ppp3r1|Egr2|Rela|Pik3cb|Ccnd1|Cdkn1a|Crtc1|Ccnd2|

3|Akt3|Ikbkb|Prkcb|Kras|

Map3k12|Dusp3|Akt2|Map3k3|Map2k1|Rela|Rps6ka5|Mapk8ip1|Mapk1|Map2k4|Fgfr3|Elk1|Nfatc4|Akt3|Pak2|Braf|Crk

c2|Pik3r1|Braf|Pten|Prkcb|Itga1|

Pip4k2b|Mylk|Iqgap2|Cfl2|Braf|Kras|Arhgef12|Pak4|

1|Tcl1|Bcl2l1|Fgf14|Cdkn1b|Akt3|Ccnd2|Met|Bcl2|Atf2|Prlr|Akt2|Ccnd1|Pkn2|Thbs1|Lamc2|Kras|
d1|Pik3ca|Fzd3|Bax|Ikbkb|Rassf1|Fgfr3|Cdkn1a|Fgf20|Fos|
:2|

l|
