# Supplementary material for: Differentiation-Associated MicroRNA Alterations in Mouse Heart-Derived Sca-1+CD31− and Sca-1+CD31+ Cells
Source: Stem Cells Int. 2016 May 19;2016:9586751. doi: 10.1155/2016/9586751 (PMC4889861; doi:10.1155/2016/9586751)
Supplement: Supplementary file 1 — Figure S1. Differentiation potential of Sca-1+CD31+cells by FACS into cardiomyocyte, smooth muscle, and endothelial cells in vitro. Table S1. The differently expressed miRNAs between Sca1+CD31−and Sca1+CD31+ cells. Table S2. KEGG pathway analyses of predicted miRNA targets. Table S3. Pathway of the differently expressed miRNAs (the top 20 pathway) and their corresponding target genes. Table S4. Cluster of the differently expressed miRNAs (the top 20 GO terms) and their corresponding target genes. Table S5. The differently expressed mRNAs between Sca1+CD31−and Sca1+CD31+ cells. Table S6. Pathway of the differently expressed mRNAs (the top 20 pathway) and their corresponding genes. Table S7. Cluster of the differently expressed mRNAs (the top 20 GO terms) and their corresponding genes. [file 9586751.f1.zip › Table S2.pdf]

Probe Set I	Accession	Transcript I	Mean Signa	Mean Signa	Fold Chang	Rank	miRNA Fea	Biotype
MIMAT000	MIMAT000	mmu-miR-3	8.484721	1.988954	90.24449	1	up	miRNA
MIMAT000	MIMAT000	mmu-miR-1	8.037142	1.584963	87.55872	2	up	miRNA
MIMAT000	MIMAT000	mmu-miR-1	9.536876	3.776693	54.19857	3	up	miRNA
MIMAT000	MIMAT000	mmu-miR-5	8.038028	2.520742	45.80033	4	up	miRNA
MIMAT000	MIMAT000	mmu-miR-4	6.64982	1.584963	33.4714	5	up	miRNA
MIMAT000	MIMAT000	mmu-miR-2	6.423538	1.584963	28.61253	6	up	miRNA
MIMAT000	MIMAT000	mmu-miR-2	11.29332	6.640055	25.16356	7	up	miRNA
MIMAT000	MIMAT000	mmu-miR-3	6.225692	1.584963	24.94587	8	up	miRNA
MIMAT000	MIMAT000	mmu-miR-1	11.59644	7.016186	23.92178	9	up	miRNA
MIMAT000	MIMAT000	mmu-miR-1	11.59644	7.016186	23.92178	9	up	miRNA
MIMAT000	MIMAT000	mmu-miR-3	6.114916	1.584963	23.10211	11	up	miRNA
MIMAT000	MIMAT000	mmu-miR-1	6.080152	1.584963	22.55209	12	up	miRNA
MIMAT000	MIMAT000	mmu-miR-1	10.60338	6.114045	22.46078	13	up	miRNA
MIMAT000	MIMAT000	mmu-miR-3	6.020404	1.584963	21.63719	14	up	miRNA
MIMAT000	MIMAT000	mmu-miR-4	10.09154	5.884948	18.46332	15	up	miRNA
MIMAT000	MIMAT000	mmu-miR-1	5.603774	1.584963	16.20999	16	up	miRNA
MIMAT000	MIMAT000	mmu-miR-4	5.519777	1.584963	15.29315	17	up	miRNA
MIMAT000	MIMAT000	mmu-miR-2	7.086901	3.171173	15.09217	18	up	miRNA
MIMAT000	MIMAT000	mmu-miR-1	5.41267	1.584963	14.1989	19	up	miRNA
MIMAT000	MIMAT000	mmu-miR-3	5.386729	1.584963	13.94587	20	up	miRNA
MIMAT000	MIMAT000	mmu-miR-5	1.667878	5.437548	-13.639	21	down	miRNA
MIMAT000	MIMAT000	mmu-miR-4	5.331895	1.584963	13.42576	22	up	miRNA
MIMAT000	MIMAT000	mmu-miR-4	5.199028	1.584963	12.24453	23	up	miRNA
MIMAT000	MIMAT000	mmu-miR-2	1.668029	5.217974	-11.7122	24	down	miRNA
MIMAT000	MIMAT000	mmu-miR-4	5.122574	1.584963	11.61254	25	up	miRNA
MIMAT000	MIMAT000	mmu-miR-1	5.066834	1.584963	11.17243	26	up	miRNA
MIMAT000	MIMAT000	mmu-miR-2	4.857437	1.584963	9.663019	27	up	miRNA
MIMAT000	MIMAT000	mmu-miR-1	4.81391	1.584963	9.375834	28	up	miRNA
MIMAT000	MIMAT000	mmu-miR-9	11.54973	8.382254	8.984742	29	up	miRNA
MIMAT000	MIMAT000	mmu-miR-1	4.710837	1.584963	8.729349	30	up	miRNA
MIMAT000	MIMAT000	mmu-miR-3	5.807811	2.714152	8.536585	31	up	miRNA
MIMAT000	MIMAT000	mmu-miR-3	4.658102	7.750097	-8.52674	32	down	miRNA
MIMAT000	MIMAT000	mmu-miR-6	9.912927	6.933577	7.886308	33	up	miRNA
MIMAT000	MIMAT000	mmu-miR-2	4.575666	1.646301	7.61775	34	up	miRNA
MIMAT000	MIMAT000	mmu-miR-3	1.584963	4.484997	-7.46444	35	down	miRNA
MIMAT000	MIMAT000	mmu-miR-3	4.481357	1.584963	7.44563	36	up	miRNA
MIMAT000	MIMAT000	mmu-miR-3	4.457592	1.584963	7.323986	37	up	miRNA
MIMAT000	MIMAT000	mmu-miR-1	10.69413	7.88496	7.008822	38	up	miRNA
MIMAT000	MIMAT000	mmu-miR-1	3.638831	6.433767	-6.94	39	down	miRNA
MIMAT000	MIMAT000	mmu-miR-6	4.323979	1.584963	6.676148	40	up	miRNA
MIMAT000	MIMAT000	mmu-miR-1	13.28055	10.54751	6.648537	41	up	miRNA
MIMAT000	MIMAT000	mmu-miR-4	4.238282	1.584963	6.291129	42	up	miRNA
MIMAT000	MIMAT000	mmu-miR-5	5.3687	7.975934	-6.09334	43	down	miRNA
MIMAT000	MIMAT000	mmu-miR-5	5.334557	2.732725	6.07057	44	up	miRNA
MIMAT000	MIMAT000	mmu-miR-2	4.173548	1.584963	6.015084	45	up	miRNA
MIMAT000	MIMAT000	mmu-miR-1	4.483769	7.066558	-5.99097	46	down	miRNA

MIMAT000	MIMAT000	mmu-miR-4	4.163549	1.584963	5.973539		47	up	miRNA
MIMAT000	MIMAT000	mmu-miR-3	4.817308	2.375287	5.434024		48	up	miRNA
MIMAT000	MIMAT000	mmu-miR-1	8.994647	11.42247	-5.38081		49	down	miRNA
MIMAT000	MIMAT000	mmu-miR-1	2.359226	4.764559	-5.29758		50	down	miRNA
MIMAT000	MIMAT000	mmu-miR-5	8.083945	5.695928	5.234374		51	up	miRNA
MIMAT000	MIMAT000	mmu-miR-1	2.80074	5.101978	-4.92881		52	down	miRNA
MIMAT000	MIMAT000	mmu-miR-1	8.688893	10.96212	-4.83405		53	down	miRNA
MIMAT000	MIMAT000	mmu-miR-5	3.43575	5.680157	-4.73842		54	down	miRNA
MIMAT000	MIMAT000	mmu-miR-4	3.825072	1.584963	4.724328		55	up	miRNA
MIMAT002	MIMAT002	mmu-miR-7	1.584963	3.786521	-4.59976		56	down	miRNA
MIMAT002	MIMAT002	mmu-miR-2	3.785021	1.584963	4.594978		57	up	miRNA
MIMAT000	MIMAT000	mmu-miR-1	6.78322	8.970211	-4.55355		58	down	miRNA
MIMAT000	MIMAT000	mmu-miR-3	5.602654	7.788252	-4.54915		59	down	miRNA
MIMAT000	MIMAT000	mmu-miR-2	4.72177	2.570816	4.441214		60	up	miRNA
MIMAT000	MIMAT000	mmu-miR-6	3.705516	1.584963	4.348606		61	up	miRNA
MIMAT002	MIMAT002	mmu-miR-6	2.549096	4.665197	-4.33521		62	down	miRNA
MIMAT001	MIMAT001	mmu-miR-5	1.869599	3.985699	-4.3352		63	down	miRNA
MIMAT000	MIMAT000	mmu-miR-1	3.226917	5.30236	-4.21474		64	down	miRNA
MIMAT000	MIMAT000	mmu-miR-2	3.825577	1.752872	4.206747		65	up	miRNA
MIMAT001	MIMAT001	mmu-miR-3	5.794992	3.76451	4.085413		66	up	miRNA
MIMAT000	MIMAT000	mmu-miR-4	2.772193	4.783041	-4.03019		67	down	miRNA
MIMAT000	MIMAT000	mmu-miR-1	1.706716	3.666596	-3.8903		68	down	miRNA
MIMAT000	MIMAT000	mmu-miR-4	3.506606	1.584963	3.788543		69	up	miRNA
MIMAT000	MIMAT000	mmu-miR-1	5.884851	3.982054	3.739375		70	up	miRNA
MIMAT000	MIMAT000	mmu-miR-3	5.167017	3.27468	3.712361		71	up	miRNA
MIMAT000	MIMAT000	mmu-miR-2	3.857911	5.722863	-3.64256		72	down	miRNA
MIMAT000	MIMAT000	mmu-miR-5	9.84741	8.061993	3.447181		73	up	miRNA
MIMAT000	MIMAT000	mmu-miR-3	3.3465	1.584963	3.390592		74	up	miRNA
MIMAT000	MIMAT000	mmu-miR-3	6.812777	5.052448	3.387754		75	up	miRNA
MIMAT000	MIMAT000	mmu-miR-6	3.333126	1.584963	3.359305		76	up	miRNA
MIMAT000	MIMAT000	mmu-miR-4	3.330934	1.584963	3.354205		77	up	miRNA
MIMAT000	MIMAT000	mmu-miR-3	7.117255	8.857476	-3.34086		78	down	miRNA
MIMAT000	MIMAT000	mmu-miR-1	6.971595	5.232104	3.339173		79	up	miRNA
MIMAT000	MIMAT000	mmu-miR-5	3.314614	1.584963	3.316476		80	up	miRNA
MIMAT000	MIMAT000	mmu-miR-3	6.826199	5.108148	3.289917		81	up	miRNA
MIMAT000	MIMAT000	mmu-miR-1	2.849456	4.528302	-3.20172		82	down	miRNA
MIMAT000	MIMAT000	mmu-miR-2	5.126674	6.796404	-3.18155		83	down	miRNA
MIMAT001	MIMAT001	mmu-miR-3	1.584963	3.23246	-3.1329		84	down	miRNA
MIMAT000	MIMAT000	mmu-miR-5	7.020565	5.386729	3.103371		85	up	miRNA
MIMAT001	MIMAT001	mmu-miR-2	3.8634	5.48558	-3.0784		86	down	miRNA
MIMAT000	MIMAT000	mmu-miR-2	4.385696	2.790757	3.020817		87	up	miRNA
MIMAT000	MIMAT000	mmu-miR-1	6.756397	8.349437	-3.01684		88	down	miRNA
MIMAT000	MIMAT000	mmu-miR-6	4.156427	2.563633	3.016329		89	up	miRNA
MIMAT000	MIMAT000	mmu-miR-2	3.399281	1.827314	2.973098		90	up	miRNA
MIMAT000	MIMAT000	mmu-miR-3	4.815563	6.387121	-2.97226		91	down	miRNA
MIMAT002	MIMAT002	mmu-miR-6	1.584963	3.143177	-2.94489		92	down	miRNA
MIMAT001	MIMAT001	mmu-miR-1	3.134626	1.584963	2.927487		93	up	miRNA

MIMAT000	MIMAT000	mmu-miR-	3.133464	1.584963	2.925131	94	up	miRNA
MIMAT002	MIMAT002	mmu-miR-	2.189386	3.722907	-2.89492	95	down	miRNA
MIMAT000	MIMAT000	mmu-miR-	3.201399	1.667878	2.894915	96	up	miRNA
MIMAT000	MIMAT000	mmu-miR-	2.730057	4.260673	-2.88909	97	down	miRNA
MIMAT000	MIMAT000	mmu-miR-	3.686972	2.158902	2.883998	98	up	miRNA
MIMAT001	MIMAT001	mmu-miR-	3.26391	1.736851	2.881977	99	up	miRNA
MIMAT000	MIMAT000	mmu-miR-	5.913881	7.414658	-2.82995	100	down	miRNA
MIMAT001	MIMAT001	mmu-miR-	3.082409	1.584963	2.823424	101	up	miRNA
MIMAT000	MIMAT000	mmu-miR-	4.044055	2.55059	2.815644	102	up	miRNA
MIMAT001	MIMAT001	mmu-miR-	2.631866	4.093677	-2.75454	103	down	miRNA
MIMAT003	MIMAT003	mmu-miR-	1.82787	3.240223	-2.66171	104	down	miRNA
MIMAT000	MIMAT000	mmu-miR-	9.112499	7.712298	2.639384	105	up	miRNA
MIMAT002	MIMAT002	mmu-miR-	1.584963	2.979682	-2.62937	106	down	miRNA
MIMAT000	MIMAT000	mmu-miR-	5.109202	6.495463	-2.614	107	down	miRNA
MIMAT000	MIMAT000	mmu-miR-	4.19293	5.57492	-2.60628	108	down	miRNA
MIMAT002	MIMAT002	mmu-miR-	4.788796	6.165166	-2.59614	109	down	miRNA
MIMAT001	MIMAT001	mmu-miR-	1.593277	2.956876	-2.57326	110	down	miRNA
MIMAT000	MIMAT000	mmu-miR-	6.968883	5.611291	2.562571	111	up	miRNA
MIMAT001	MIMAT001	mmu-miR-	3.964472	2.608453	2.559779	112	up	miRNA
MIMAT002	MIMAT002	mmu-miR-	2.897532	4.244485	-2.54374	113	down	miRNA
MIMAT000	MIMAT000	mmu-miR-	11.53997	10.19406	2.54191	114	up	miRNA
MIMAT000	MIMAT000	mmu-miR-	9.321246	10.65528	-2.52107	115	down	miRNA
MIMAT001	MIMAT001	mmu-miR-	2.918216	1.584963	2.519702	116	up	miRNA
MIMAT000	MIMAT000	mmu-miR-	7.611825	6.285853	2.507017	117	up	miRNA
MIMAT000	MIMAT000	mmu-miR-	6.789549	5.467718	2.499832	118	up	miRNA
MIMAT000	MIMAT000	mmu-miR-	2.125762	3.434487	-2.47723	119	down	miRNA
MIMAT001	MIMAT001	mmu-miR-	1.780133	3.085279	-2.47109	120	down	miRNA
MIMAT002	MIMAT002	mmu-miR-	2.745689	4.050826	-2.47107	121	down	miRNA
MIMAT000	MIMAT000	mmu-miR-	3.149227	4.454363	-2.47107	122	down	miRNA
MIMAT000	MIMAT000	mmu-miR-	1.584963	2.883628	-2.46001	123	down	miRNA
MIMAT002	MIMAT002	mmu-miR-	2.356712	3.650378	-2.4515	124	down	miRNA
MIMAT000	MIMAT000	mmu-miR-	2.154894	3.440136	-2.43723	125	down	miRNA
MIMAT002	MIMAT002	mmu-miR-	1.61063	2.895871	-2.43723	126	down	miRNA
MIMAT000	MIMAT000	mmu-miR-	8.654423	9.933396	-2.42666	127	down	miRNA
MIMAT002	MIMAT002	mmu-miR-	4.252475	5.516099	-2.40098	128	down	miRNA
MIMAT000	MIMAT000	mmu-miR-	6.398104	7.660621	-2.39914	129	down	miRNA
MIMAT000	MIMAT000	mmu-miR-	2.631124	3.878469	-2.37404	130	down	miRNA
MIMAT001	MIMAT001	mmu-miR-	2.08379	3.331135	-2.37404	130	down	miRNA
MIMAT000	MIMAT000	mmu-miR-	2.577708	3.805231	-2.34165	132	down	miRNA
MIMAT002	MIMAT002	mmu-miR-	1.584963	2.80881	-2.33569	133	down	miRNA
MIMAT002	MIMAT002	mmu-miR-	2.979682	4.196771	-2.32477	134	down	miRNA
MIMAT002	MIMAT002	mmu-miR-	3.484053	4.698417	-2.32039	135	down	miRNA
MIMAT000	MIMAT000	mmu-miR-	8.273575	9.477687	-2.30395	136	down	miRNA
MIMAT002	MIMAT002	mmu-miR-	1.934711	3.133382	-2.29528	137	down	miRNA
MIMAT002	MIMAT002	mmu-miR-	1.736659	2.923349	-2.2763	138	down	miRNA
MIMAT001	MIMAT001	mmu-miR-	2.758825	1.584963	2.256148	139	up	miRNA
MIMAT000	MIMAT000	mmu-miR-	8.338335	9.503254	-2.24221	140	down	miRNA

MIMAT000	MIMAT000	mmu-miR-	9.794918	10.95819	-2.23965	141	down	miRNA
MIMAT002	MIMAT002	mmu-miR-	3.095116	1.950569	2.210767	142	up	miRNA
MIMAT002	MIMAT002	mmu-miR-	2.23873	3.37501	-2.19814	143	down	miRNA
MIMAT002	MIMAT002	mmu-miR-	1.606882	2.743162	-2.19814	144	down	miRNA
MIMAT002	MIMAT002	mmu-miR-	1.584963	2.718498	-2.19396	145	down	miRNA
MIMAT003	MIMAT003	mmu-miR-	1.805559	2.930589	-2.18106	146	down	miRNA
MIMAT000	MIMAT000	mmu-miR-	4.80116	3.687325	2.164202	147	up	miRNA
MIMAT002	MIMAT002	mmu-miR-	2.680554	3.793564	-2.16297	148	down	miRNA
MIMAT002	MIMAT002	mmu-miR-	3.366937	4.47702	-2.15858	149	down	miRNA
MIMAT002	MIMAT002	mmu-miR-	2.69413	1.584963	2.157211	150	up	miRNA
MIMAT002	MIMAT002	mmu-miR-	1.770894	2.876666	-2.15214	151	down	miRNA
MIMAT001	MIMAT001	mmu-miR-	1.981479	3.086615	-2.15119	152	down	miRNA
MIMAT000	MIMAT000	mmu-miR-	7.571301	8.671329	-2.14359	153	down	miRNA
MIMAT000	MIMAT000	mmu-miR-	12.53249	13.62874	-2.13799	154	down	miRNA
MIMAT000	MIMAT000	mmu-miR-	1.584963	2.680554	-2.13701	155	down	miRNA
MIMAT002	MIMAT002	mmu-miR-	1.584963	2.680554	-2.13701	155	down	miRNA
MIMAT001	MIMAT001	mmu-miR-	3.084125	1.990059	2.134748	157	up	miRNA
MIMAT000	MIMAT000	mmu-miR-	9.672583	8.580685	2.131543	158	up	miRNA
MIMAT000	MIMAT000	mmu-miR-	3.781378	4.859857	-2.11181	159	down	miRNA
MIMAT000	MIMAT000	mmu-miR-	7.47449	8.550306	-2.10791	160	down	miRNA
MIMAT000	MIMAT000	mmu-miR-	1.998072	3.070021	-2.10227	161	down	miRNA
MIMAT002	MIMAT002	mmu-miR-	2.896343	1.837924	2.082648	162	up	miRNA
MIMAT003	MIMAT003	mmu-miR-	3.985699	5.037278	-2.0728	163	down	miRNA
MIMAT000	MIMAT000	mmu-miR-	9.689053	10.73872	-2.07005	164	down	miRNA
MIMAT000	MIMAT000	mmu-miR-	6.67311	5.63136	2.058723	165	up	miRNA
MIMAT000	MIMAT000	mmu-miR-	8.735561	9.775533	-2.05619	166	down	miRNA
MIMAT000	MIMAT000	mmu-miR-	10.87946	11.90288	-2.03273	167	down	miRNA
MIMAT000	MIMAT000	mmu-miR-	3.278211	2.265535	2.01765	168	up	miRNA
MIMAT000	MIMAT000	mmu-miR-	6.882004	7.89329	-2.01571	169	down	miRNA
MIMAT000	MIMAT000	mmu-miR-	2.870142	1.864125	2.008359	170	up	miRNA
