# Supplementary material for: Differentiation-Associated MicroRNA Alterations in Mouse Heart-Derived Sca-1+CD31− and Sca-1+CD31+ Cells
Source: Stem Cells Int. 2016 May 19;2016:9586751. doi: 10.1155/2016/9586751 (PMC4889861; doi:10.1155/2016/9586751)
Supplement: Supplementary file 1 — Figure S1. Differentiation potential of Sca-1+CD31+cells by FACS into cardiomyocyte, smooth muscle, and endothelial cells in vitro. Table S1. The differently expressed miRNAs between Sca1+CD31−and Sca1+CD31+ cells. Table S2. KEGG pathway analyses of predicted miRNA targets. Table S3. Pathway of the differently expressed miRNAs (the top 20 pathway) and their corresponding target genes. Table S4. Cluster of the differently expressed miRNAs (the top 20 GO terms) and their corresponding target genes. Table S5. The differently expressed mRNAs between Sca1+CD31−and Sca1+CD31+ cells. Table S6. Pathway of the differently expressed mRNAs (the top 20 pathway) and their corresponding genes. Table S7. Cluster of the differently expressed mRNAs (the top 20 GO terms) and their corresponding genes. [file 9586751.f1.zip › Table S3.pdf]

Pathway ID	Pathway N	Diff Gene C	Gene Amou	Enrichment	p-value	FDR	Gene Symb	Rank
4151	PI3K-Akt si	112	356	5.425027	7.82E-51	1.99E-48	|Tnc|Fgf4|	1
5200	Pathways i	99	326	5.236625	1.64E-43	2.09E-41	|Fzd5|Fgf7	2
4010	MAPK sign	88	259	5.85891	3.44E-43	2.92E-41	|Bdnf|Atf2	3
5205	Proteoglyc	81	230	6.07283	3.57E-41	2.27E-39	|Hspg2|Hs	4
1100	Metabolic	179	1242	2.485223	6.59E-29	3.36E-27	|Dgkd|Dak	5
4810	Regulation	63	217	5.006275	6.99E-27	2.97E-25	|Fgf16|Fgd	6
4510	Focal adhe	61	205	5.131093	1.04E-26	3.79E-25	|Itga3|Pter	7
4722	Neurotropl	45	123	6.308721	3.41E-24	1.09E-22	|Pik3ca|M	8
4360	Axon guida	46	133	5.964034	1.66E-23	4.70E-22	|Unc5d|Se	9
5166	HTLV-I infe	68	290	4.043383	5.05E-23	1.29E-21	|Il15ra|Tgf	10
4390	Hippo sign	49	156	5.416333	7.65E-23	1.77E-21	|Wnt3|Ccr	11
4144	Endocytosi	59	225	4.521717	9.41E-23	2.00E-21	|Src|Dnm1	12
4150	mTOR sign	31	62	8.621919	6.39E-22	1.25E-20	|Eif4b|Ddi	13
4910	Insulin sign	45	141	5.503352	2.16E-21	3.93E-20	|Pik3ca|Gy	14
4310	Wnt signali	46	148	5.359571	2.64E-21	4.48E-20	|Siah1a|Ca	15
5161	Hepatitis B	46	149	5.323601	3.60E-21	5.74E-20	|Fasl|Nras	16
4062	Chemokine	53	199	4.59258	6.74E-21	1.01E-19	|Gnai3|Sh	17
4141	Protein pro	48	169	4.897658	2.54E-20	3.59E-19	|Rpn2|Ube	18
4912	GnRH signa	34	89	6.587533	2.89E-19	3.88E-18	|Pla2g4d|	19
4728	Dopaminer	41	135	5.237017	1.05E-18	1.33E-17	|Ppp2r3a|	20
4012	ErbB signal	33	87	6.540766	1.27E-18	1.55E-17	|Crkl|Egfr|	21
5215	Prostate ca	33	89	6.393782	2.89E-18	3.35E-17	|Chuk|Ccn	22
5220	Chronic my	30	74	6.990745	5.34E-18	5.92E-17	|Hdac1|Ctl	23
4916	Melanogen	34	100	5.862905	2.03E-17	2.16E-16	|Creb3l4|	24
5100	Bacterial in	30	78	6.632245	3.11E-17	3.18E-16	|Elmo1|Cd	25
5203	Viral carcin	52	236	3.79949	1.18E-16	1.16E-15	|Gtf2a1|Cc	26
4120	Ubiquitin m	39	143	4.702865	4.46E-16	4.21E-15	|Nedd4|Ub	27
5202	Transcripti	44	181	4.191872	6.09E-16	5.54E-15	|Cdkn1b|D	28
4740	Olfactory t	11	1080	0.175632	9.56E-16	8.31E-15	|Adrbk2|A	29
4915	Estrogen si	32	99	5.573766	9.77E-16	8.31E-15	|Shc2|Pik3	30
4725	Cholinergic	34	114	5.142899	1.89E-15	1.55E-14	|Map2k1|	31
5160	Hepatitis C	37	136	4.691338	2.82E-15	2.25E-14	|Mapk12|	32
4660	T cell recep	33	110	5.173151	4.09E-15	3.16E-14	|Cblb|Tnf|	33
5221	Acute myel	24	57	7.260563	4.47E-15	3.35E-14	|Pik3ca|Cc	34
5222	Small cell l	29	86	5.814782	6.39E-15	4.65E-14	|Itgav|Pik3	35
4622	RIG-I-like r	26	69	6.497678	7.80E-15	5.52E-14	|Mavs|Cyl	36
4512	ECM-recep	29	87	5.747946	9.05E-15	6.23E-14	|Itgav|Lam	37
4380	Osteoclast	35	127	4.752239	1.06E-14	7.10E-14	|Cyld|Rela	38
5169	Epstein-Ba	47	220	3.683911	1.25E-14	8.10E-14	|Tbk1|Ywh	39
5164	Influenza A	41	172	4.11045	1.27E-14	8.10E-14	|Trim25|M	40
5214	Glioma	25	66	6.531757	2.27E-14	1.41E-13	|Braf|Pik3	41
5212	Pancreatic	25	67	6.434268	3.43E-14	2.08E-13	|Chuk|Pik3	42
4020	Calcium sig	42	185	3.914817	3.69E-14	2.19E-13	|Cacna1c|	43
4666	Fc gamma	29	92	5.435557	4.76E-14	2.76E-13	|Arpc4|Pp	44
4911	Insulin secr	28	88	5.486675	1.02E-13	5.75E-13	|Trpm4|Sn	45
4370	VEGF signa	24	66	6.270486	2.23E-13	1.23E-12	|Ptk2|Pla2	46

4630	Jak-STAT si	37	156	4.089884	3.06E-13	1.66E-12	|Socs7|Cbl	47
5218	Melanoma	25	73	5.905424	3.38E-13	1.80E-12	|Fgf16|Fgf	48
4270	Vascular sn	33	127	4.480682	3.92E-13	2.04E-12	|Ednra|Prk	49
5162	Measles	34	140	4.187789	1.41E-12	7.19E-12	|Pik3ca|Ifr	50
4723	Retrograde	29	104	4.808378	1.59E-12	7.80E-12	|Mgll|Map	51
5142	Chagas dise	29	104	4.808378	1.59E-12	7.80E-12	|Akt1|Ppp	52
4340	Hedgehog s	20	49	7.038301	1.93E-12	9.31E-12	|Wnt3|Lrp	53
4080	Neuroactiv	50	282	3.057418	3.44E-12	1.62E-11	|Adcyap1r	54
4114	Oocyte me	30	115	4.498392	4.29E-12	1.99E-11	|Ppp2r5a|	55
4530	Tight juncti	33	138	4.123526	4.83E-12	2.20E-11	|Mpp5|Prk	56
4514	Cell adhesi	36	163	3.808455	6.23E-12	2.79E-11	|Cdh3|Nrx	57
5213	Endometria	20	52	6.632245	7.28E-12	3.18E-11	|Braf|Egfr|	58
4914	Progestero	26	89	5.037525	7.36E-12	3.18E-11	|Adcy2|Cd	59
5210	Colorectal	22	64	5.927569	8.71E-12	3.70E-11	|Pik3cd|Cc	60
4066	HIF-1 signa	29	113	4.42541	1.54E-11	6.35E-11	|Prkcb|Ldh	61
5145	Toxoplasm	29	113	4.42541	1.54E-11	6.35E-11	|Il10ra|Ccr	62
4724	Glutamater	29	117	4.274114	3.91E-11	1.58E-10	|Slc38a1|G	63
4115	p53 signali	22	69	5.498035	4.81E-11	1.89E-10	|Bai1|Bax|	64
5211	Renal cell c	22	69	5.498035	4.81E-11	1.89E-10	|Pak4|Nras	65
3015	mRNA surv	26	97	4.622059	6.35E-11	2.45E-10	|Wdr33|Pp	66
5414	Dilated car	25	90	4.789955	6.45E-11	2.46E-10	|Tnf|Lmna	67
4664	Fc epsilon	22	71	5.343161	9.08E-11	3.40E-10	|Mapk3|N	68
4713	Circadian e	26	99	4.528685	1.05E-10	3.87E-10	|Gnb5|Gng	69
4142	Lysosome	29	124	4.032833	1.79E-10	6.52E-10	|Slc11a2|M	70
4060	Cytokine-cy	45	266	2.917191	2.17E-10	7.77E-10	|Ghr|Tgfb2	71
4971	Gastric acid	22	74	5.126546	2.25E-10	7.77E-10	|Prkacb|M	72
5412	Arrhythmo	22	74	5.126546	2.25E-10	7.77E-10	|Itgb1|Tcf7	73
5223	Non-small	19	55	5.956962	2.26E-10	7.77E-10	|Raf1|Pik3	74
5168	Herpes sim	39	211	3.18725	2.53E-10	8.61E-10	|Socs3|Eif2	75
4720	Long-term	21	70	5.173151	4.91E-10	1.65E-09	|Raf1|Rps6	76
4960	Aldosteron	16	40	6.897535	5.22E-10	1.73E-09	|Atp1b4|M	77
5410	Hypertroph	23	84	4.721527	5.37E-10	1.75E-09	|Itgav|Itga	78
4920	Adipocytok	21	71	5.10029	6.59E-10	2.13E-09	|Camkk1|F	79
4540	Gap junctio	23	88	4.506912	1.47E-09	4.70E-09	|Adcy2|Cd	80
3040	Spliceosom	29	137	3.650155	2.22E-09	6.98E-09	|Snrnp40|S	81
4961	Endocrine a	18	56	5.542662	2.66E-09	8.27E-09	|Ap2a1|At	82
4962	Vasopressi	16	44	6.270486	2.77E-09	8.51E-09	|Aqp2|Prka	83
4726	Serotonerg	28	130	3.714057	2.81E-09	8.53E-09	|Slc18a2|G	84
4930	Type II diab	17	50	5.862905	2.84E-09	8.53E-09	|Ikbkb|Hk1	85
4970	Salivary sec	21	77	4.702865	3.43E-09	1.02E-08	|Adcy3|Ad	86
5032	Morphine a	23	93	4.264605	4.78E-09	1.40E-08	|Arrb1|Gn	87
4620	Toll-like rec	24	101	4.097545	5.15E-09	1.49E-08	|Chuk|Map	88
5146	Amoebiasis	26	120	3.736165	9.38E-09	2.69E-08	|Pik3ca|Il1	89
4727	GABAergic	22	90	4.21516	1.33E-08	3.78E-08	|Gad2|Prka	90
5217	Basal cell ca	17	55	5.329913	1.49E-08	4.18E-08	|Wnt3a|W	91
4721	Synaptic ve	18	63	4.926811	2.19E-08	6.04E-08	|Atp6v1a|	92
5031	Amphetam	19	70	4.68047	2.20E-08	6.04E-08	|Grin2b|Pr	93

4662	B cell recep	20	78	4.421497	2.66E-08	7.21E-08	|Mapk3|Ik	94
230	Purine met	31	173	3.089936	4.07E-08	1.09E-07	|Gmps|Pol	95
5216	Thyroid car	12	29	7.135381	5.82E-08	1.55E-07	|Tcf7|Braf	96
4520	Adherens ju	19	75	4.368439	7.53E-08	1.98E-07	|Smad3|Pv	97
4350	TGF-beta si	20	83	4.155142	8.30E-08	2.16E-07	|Inhbe|Tnf	98
564	Glycerophc	21	91	3.979347	8.65E-08	2.23E-07	|Pla2g4d|D	99
532	Glycosamir	10	20	8.621919	9.16E-08	2.34E-07	|B3gat3|Cl	100
4210	Apoptosis	20	84	4.105676	1.03E-07	2.60E-07	|Rela|Ikbkl	101
4330	Notch signa	15	49	5.278726	1.31E-07	3.28E-07	|Dll4|Hdac	102
5034	Alcoholism	33	202	2.817063	1.48E-07	3.66E-07	|H2afj|Nra	103
5030	Cocaine ad	15	50	5.173151	1.77E-07	4.34E-07	|Nfkb1|Gri	104
4110	Cell cycle	25	129	3.341829	1.91E-07	4.63E-07	|Cdk2|Cdk	105
4070	Phosphatid	19	81	4.044851	2.84E-07	6.84E-07	|Prkcb|Pip	106
4621	NOD-like re	16	59	4.676295	3.17E-07	7.52E-07	|Tnf|Mapk	107
5152	Tuberculos	30	179	2.890029	3.19E-07	7.52E-07	|Rela|Map	108
4972	Pancreatic	21	103	3.515734	8.15E-07	1.91E-06	|Plcb1|Atp	109
4670	Leukocyte t	23	121	3.277754	8.78E-07	2.04E-06	|Gnai3|Thy	110
561	Glycerolipi	15	56	4.618885	9.19E-07	2.11E-06	|Aldh3a2|L	111
4973	Carbohydra	13	45	4.981553	2.07E-06	4.71E-06	|Prkcb|Akt	112
562	Inositol pho	15	61	4.240288	3.03E-06	6.83E-06	|Inpp5b|Pl	113
4320	Dorso-vent	9	22	7.054297	3.65E-06	8.17E-06	|Grb2|Map	114
4974	Protein dig	18	88	3.527149	5.10E-06	1.13E-05	|Atp1b3|A	115
5020	Prion disea	11	35	5.419492	5.58E-06	1.23E-05	|Prnp|Stip	116
4650	Natural kill	22	125	3.034915	5.94E-06	1.29E-05	|Sh2d1a|M	117
5133	Pertussis	16	74	3.728397	8.50E-06	1.84E-05	|Gnai3|Ma	118
5140	Leishmania	15	66	3.919054	8.75E-06	1.88E-05	|Nfkb1|Jak	119
4064	NF-kappa B	19	103	3.180902	1.35E-05	2.88E-05	|Traf2|Chu	120
4730	Long-term	14	61	3.957602	1.60E-05	3.38E-05	|Gna11|Plc	121
5132	Salmonella	16	79	3.492423	2.07E-05	4.32E-05	|Arpc5|Arp	122
5016	Huntington	27	189	2.463405	2.87E-05	5.96E-05	|Pparg|Ap2	123
512	Mucin type	9	28	5.542662	3.69E-05	7.59E-05	|Galnt3|Gc	124
4640	Hematopoi	16	88	3.135243	8.47E-05	0.000173	|Itga3|Itga	125
410	beta-Alanir	9	31	5.006275	9.20E-05	0.000186	|Aldh1b1|(	126
5219	Bladder car	10	39	4.421497	0.000114	0.000229	|Raf1|Map	127
4950	Maturity or	8	26	5.305796	0.000154	0.000307	|Nr5a2|On	128
330	Arginine ar	12	57	3.630282	0.000174	0.000344	|Cndp1|Ac	129
4913	Ovarian ste	12	58	3.56769	0.000208	0.000407	|Cyp1b1|A	130
510	N-Glycan b	11	50	3.793644	0.000223	0.000435	|Mgat3|M	131
5134	Legionellos	12	59	3.507221	0.000247	0.000477	|Nfkb1|Tnf	132
3013	RNA transp	23	172	2.305862	0.000399	0.000765	|Clns1a|Th	133
514	Other types	8	30	4.598357	0.000469	0.000886	|B3gat1|Pc	134
3060	Protein exp	8	30	4.598357	0.000469	0.000886	|Immp1l|S	135
240	Pyrimidine	16	102	2.704916	0.000515	0.000966	|Uck1|Nm	136
100	Steroid bio	6	17	6.08606	0.000537	0.001	|Dhcr24|Cy	137
62	Fatty acid e	7	24	5.029453	0.000631	0.001167	|Hadha|Elc	138
4975	Fat digestic	9	40	3.879863	0.000777	0.001415	|Ppap2b|D	139
5033	Nicotine ac	9	40	3.879863	0.000777	0.001415	|Gabra3|Sl	140

4964	Proximal tu	6	20	5.173151	0.001448	0.002614	|Atp1b4|A	141
4130	SNARE inte	8	35	3.941449	0.001456	0.002614	|Snap25|V	142
4623	Cytosolic D	11	62	3.05939	0.001602	0.002857	|Rela|Polr3	143
4744	Phototrans	7	28	4.310959	0.001761	0.003118	|Rho|Gnat	144
4978	Mineral ab:	9	46	3.373794	0.002287	0.004023	|Slc30a1|S	145
603	Glycosphin	5	15	5.747946	0.002404	0.004198	|St8sia1|N	146
650	Butanoate	7	30	4.023562	0.002734	0.004743	|Acsm5|Ab	147
640	Propanoate	7	31	3.89377	0.003357	0.005745	|Aldh1b1|/	148
4710	Circadian rl	7	31	3.89377	0.003357	0.005745	|Bhlhe40|I	149
5144	Malaria	9	49	3.167235	0.003648	0.006202	|Sdc2|Tnf|	150
4976	Bile secreti	11	72	2.634475	0.005607	0.009469	|Adcy2|At	151
4672	Intestinal ir	8	43	3.208156	0.005937	0.00996	|Pigr|Cd86	152
4140	Regulation	7	35	3.448767	0.007008	0.011605	|Atg12|Prk	153
5143	African tryp	7	35	3.448767	0.007008	0.011605	|Il12a|Gna	154
5010	Alzheimer's	21	183	1.978801	0.007195	0.011836	|Atp5g2|A	155
4966	Collecting c	6	27	3.831964	0.007797	0.012746	|Atp6v1g1	156
5014	Amyotroph	9	55	2.821719	0.008266	0.013426	|Bax|Cycs|	157
10	Glycolysis /	10	66	2.612703	0.009106	0.014691	|Aldh3a2|I	158
3022	Basal trans	8	46	2.998928	0.00916	0.014691	|Taf11|Taf	159
600	Sphingolipi	8	47	2.935121	0.010488	0.016657	|Kdsr|Neu	160
3018	RNA degrac	11	78	2.431823	0.010517	0.016657	|Parn|Pan3	161
4260	Cardiac mu	11	79	2.401041	0.011592	0.018247	|Atp1a2|Sl	162
3410	Base excisi	7	39	3.095048	0.0131	0.020494	|Pold3|Xrc	163
310	Lysine degr	8	49	2.81532	0.01358	0.021115	|Aldh1b1|I	164
5323	Rheumatoi	11	84	2.258122	0.018324	0.028083	|Atp6v1f|A	165
533	Glycosamir	4	15	4.598357	0.018392	0.028083	|B3gnt1|Fu	166
604	Glycosphin	4	15	4.598357	0.018392	0.028083	|St3gal5|St	167
4145	Phagosome	19	180	1.820183	0.020468	0.031067	|Itgb3|Rab	168
534	Glycosamir	5	24	3.592466	0.022012	0.033213	|Hs3st3a1|	169
770	Pantothena	4	16	4.310959	0.023413	0.035119	|Aasdhppt	170
500	Starch and	8	54	2.554643	0.024286	0.036217	|Pygm|Kl|	171
601	Glycosphin	5	25	3.448767	0.026226	0.038882	|B3gnt5|Fu	172
1230	Biosynthes	10	77	2.239459	0.026704	0.039362	|Prps1|Pga	173
250	Alanine, as	6	35	2.956086	0.028809	0.04222	|Gad2|Gfp	174
450	Selenocom	4	17	4.057373	0.029234	0.042598	|Papss2|Cc	175
5340	Primary im	6	36	2.873973	0.032917	0.047692	|Cd4|Icos|	176
920	Sulfur meta	3	11	4.702865	0.045246	0.065185	|Chst11|Pa	177
51	Fructose ar	6	39	2.652898	0.047615	0.067832	|Sord|Mtm	178
270	Cysteine ar	6	39	2.652898	0.047615	0.067832	|Dnmt3b|(	179
