# Supplementary material for: Differentiation-Associated MicroRNA Alterations in Mouse Heart-Derived Sca-1+CD31− and Sca-1+CD31+ Cells
Source: Stem Cells Int. 2016 May 19;2016:9586751. doi: 10.1155/2016/9586751 (PMC4889861; doi:10.1155/2016/9586751)
Supplement: Supplementary file 1 — Figure S1. Differentiation potential of Sca-1+CD31+cells by FACS into cardiomyocyte, smooth muscle, and endothelial cells in vitro. Table S1. The differently expressed miRNAs between Sca1+CD31−and Sca1+CD31+ cells. Table S2. KEGG pathway analyses of predicted miRNA targets. Table S3. Pathway of the differently expressed miRNAs (the top 20 pathway) and their corresponding target genes. Table S4. Cluster of the differently expressed miRNAs (the top 20 GO terms) and their corresponding target genes. Table S5. The differently expressed mRNAs between Sca1+CD31−and Sca1+CD31+ cells. Table S6. Pathway of the differently expressed mRNAs (the top 20 pathway) and their corresponding genes. Table S7. Cluster of the differently expressed mRNAs (the top 20 GO terms) and their corresponding genes. [file 9586751.f1.zip › Table S4.pdf]

GO ID	GO Name	ene Counts	ie Amount in	richment Sco	p-value
GO:0006355	regulation of transcription, DNA-dependent	311	1852	4.8773469	8.82E-124
GO:0006351	transcription, DNA-dependent	290	1756	4.7966466	3.97E-113
GO:0045944	positive regulation of transcription from RNA polymera	157	745	6.1207919	7.55E-76
GO:0006810	transport	213	1712	3.6136	8.40E-60
GO:0007275	multicellular organismal development	150	954	4.5667488	8.08E-55
GO:0000122	negative regulation of transcription from RNA polyme	108	561	5.5914588	3.50E-48
GO:0045893	positive regulation of transcription, DNA-dependent	104	523	5.7755838	8.46E-48
GO:0006468	protein phosphorylation	106	611	5.0388206	6.97E-43
GO:0007399	nervous system development	75	307	7.0955673	2.99E-41
GO:0016310	phosphorylation	109	684	4.62844	1.74E-40
GO:0030154	cell differentiation	101	636	4.6124163	1.97E-37
GO:0006915	apoptotic process	91	540	4.8945399	6.90E-36
GO:0008285	negative regulation of cell proliferation	66	315	6.0855189	4.32E-32
GO:0007155	cell adhesion	82	500	4.7633017	1.45E-31
GO:0006357	regulation of transcription from RNA polymerase II pro	55	241	6.6284179	6.55E-29
GO:0015031	protein transport	81	538	4.3728742	1.46E-28
GO:0045892	negative regulation of transcription, DNA-dependent	70	429	4.7391994	5.90E-27
GO:0008284	positive regulation of cell proliferation	65	382	4.9421308	3.67E-26
GO:0043066	negative regulation of apoptotic process	72	480	4.3566783	2.12E-25
GO:0016568	chromatin modification	52	251	6.0171919	2.84E-25

FDR	Gene Symbol	Rank
3.49E-120	|Ikzf4|Zzz3|L	1
7.86E-110	|Esr2|Thrap3	2
9.96E-73	|Cask|Nr2f1|	3
8.31E-57	|Rab11a|Bic	4
6.40E-52	|Nr6a1|Diap2	5
2.31E-45	|Ciita|Sox6|F	6
4.78E-45	|Ikzf1|Nr4a2|	7
3.45E-40	|Glyctk|Ttbk1	8
1.31E-38	|Hoxa1|Amig	9
6.89E-38	|Tesk1|Etnk1	10
7.10E-35	|Myt1|Fzd5|S	11
2.28E-33	|Ntn1|Traf1|E	12
1.32E-29	|Rbpj|Tob1|F	13
4.11E-29	|Arvcf|Lamc2	14
1.73E-26	|Sp4|Trak2|F	15
3.62E-26	|Sec63|Rab2	16
1.37E-24	|Foxp1|Kdm5	17
8.07E-24	|Ntn1|Id4|Ac	18
4.42E-23	|Ivns1abp|Br	19
5.61E-23	|Prkcb|Suv39	20
