# Supplementary material for: Differentiation-Associated MicroRNA Alterations in Mouse Heart-Derived Sca-1+CD31− and Sca-1+CD31+ Cells
Source: Stem Cells Int. 2016 May 19;2016:9586751. doi: 10.1155/2016/9586751 (PMC4889861; doi:10.1155/2016/9586751)
Supplement: Supplementary file 1 — Figure S1. Differentiation potential of Sca-1+CD31+cells by FACS into cardiomyocyte, smooth muscle, and endothelial cells in vitro. Table S1. The differently expressed miRNAs between Sca1+CD31−and Sca1+CD31+ cells. Table S2. KEGG pathway analyses of predicted miRNA targets. Table S3. Pathway of the differently expressed miRNAs (the top 20 pathway) and their corresponding target genes. Table S4. Cluster of the differently expressed miRNAs (the top 20 GO terms) and their corresponding target genes. Table S5. The differently expressed mRNAs between Sca1+CD31−and Sca1+CD31+ cells. Table S6. Pathway of the differently expressed mRNAs (the top 20 pathway) and their corresponding genes. Table S7. Cluster of the differently expressed mRNAs (the top 20 GO terms) and their corresponding genes. [file 9586751.f1.zip › Table S5.pdf]

Probe Set I	Gene Symb	Accession N	Gene Desc	Database S	Mean Signa	Mean Signa	Fold Chang	Rank
TC0100001	Ptgs2	NM_01119	"Mus musc	RefSeq	10.42688	4.994938	43.16952	1
TC020000C	Tnfaip6	NM_00939	"Mus musc	RefSeq	9.135637	3.854242	38.89182	2
TC1500001	C7	NM_00124	"Mus musc	RefSeq	9.826669	4.579502	37.97997	3
TC1100004	Abca6	NM_14721	"Mus musc	RefSeq	9.349654	4.416313	30.55509	4
TC110000C	Gfpt2	NM_01352	"Mus musc	RefSeq	10.72557	5.903226	28.29249	5
TC1100004	Abca9	NM_14722	"Mus musc	RefSeq	9.317104	4.52298	27.74439	6
TC1700002	C3	NM_00977	"Mus musc	RefSeq	11.07218	6.372789	25.98105	7
TC0100001	Kcnt2	NM_00108	"Mus musc	RefSeq	8.345459	3.655662	25.8089	8
TC110000C	Efemp1	NM_14601	"Mus musc	RefSeq	9.064316	4.388436	25.56114	9
TC0600001	Mfap5	NM_01577	"Mus musc	RefSeq	10.35433	5.723978	24.76703	10
TC150000C	Col14a1	NM_18127	"Mus musc	RefSeq	9.704146	5.110921	24.13785	11
TC1000001	Lama2	NM_00848	"Mus musc	RefSeq	10.38365	5.859805	23.00453	12
TC0700001	Olfml1	NM_17290	"Mus musc	RefSeq	7.867038	3.39464	22.19862	13
TC010000C	Pi15	NM_05319	"Mus musc	RefSeq	7.843122	3.379739	22.06034	14
TC0500001	Gpr133	NM_00108	"Mus musc	RefSeq	9.662302	5.233691	21.53499	15
TC1600001	Pla1a	NM_13410	"Mus musc	RefSeq	9.273029	4.861741	21.27796	16
TC050000C	Sema3c	NM_01365	"Mus musc	RefSeq	8.252346	3.849847	21.14873	17
TC130000C	Omd	NM_01205	"Mus musc	RefSeq	7.805657	3.415406	20.96994	18
TC0200003	Scn7a	NM_00913	"Mus musc	RefSeq	10.01564	5.63295	20.86027	19
TC1200001	Serpina3n	NM_00925	"Mus musc	RefSeq	9.498101	5.1176	20.8287	20
TC1300002	Vcan	NM_00113	"Mus musc	RefSeq	8.736898	4.378549	20.51133	21
TC0X00001	Srpx2	NM_02683	"Mus musc	RefSeq	8.813413	4.485347	20.08527	22
TC1100003	Abca8a	NM_15314	"Mus musc	RefSeq	10.44816	6.131154	19.9319	23
TC0500003	Medag	NM_02751	"Mus musc	RefSeq	9.514001	5.202251	19.8594	24
TC170000C	Tnxb	NM_03117	"Mus musc	RefSeq	10.60375	6.324486	19.41725	25
TC0900001	Pcolce2	NM_02962	"Mus musc	RefSeq	10.02489	5.747079	19.39762	26
TC0300002	Igsf10	NM_00116	"Mus musc	RefSeq	8.420385	4.1446	19.37044	27
TC110000C	Egfr	NM_00791	"Mus musc	RefSeq	8.98475	4.720648	19.21421	28
TC050000C	Il6	NM_03116	"Mus musc	RefSeq	8.234257	4.002262	18.79133	29
TC0800001	Mmp2	NM_00861	"Mus musc	RefSeq	11.35605	7.124074	18.79112	30
TC1000001	Tcf21	NM_01154	"Mus musc	RefSeq	9.709562	5.503065	18.46213	31
TC0800001	Dpep1	NM_00787	"Mus musc	RefSeq	10.58843	6.386493	18.40385	32
TC0900002	Loxl1	NM_01072	"Mus musc	RefSeq	10.50025	6.318099	18.15321	33
TC0900002	Islr	NM_00119	"Mus musc	RefSeq	9.470211	5.295041	18.06556	34
TC070000C	Pcsk6	NM_01104	"Mus musc	RefSeq	10.74182	6.60007	17.65193	35
TC130000C	Tgfbi	NM_00936	"Mus musc	RefSeq	10.18431	6.05162	17.54138	36
TC1100003	Abca8b	NM_01385	"Mus musc	RefSeq	8.992709	4.869924	17.42136	37
TC080000C	Sfrp1	NM_01383	"Mus musc	RefSeq	9.846081	5.735999	17.26863	38
TC190000C	Lrrn4cl	NM_00101	"Mus musc	RefSeq	8.935759	4.827095	17.25167	39
TC0100001	Myoc	NM_01086	"Mus musc	RefSeq	8.680308	4.573011	17.23533	40
TC1100001	Ccl7	NM_01365	"Mus musc	RefSeq	8.056894	3.958473	17.12962	41
TC0600002	Antxr1	NM_05404	"Mus musc	RefSeq	9.512844	5.420735	17.05484	42
TC0200005	Sulf2	NM_00125	"Mus musc	RefSeq	10.35372	6.264021	17.02641	43
TC030000C	Postn	NM_00119	"Mus musc	RefSeq	9.414208	5.347497	16.75722	44
TC1000001	Lum	NM_00852	"Mus musc	RefSeq	10.47261	6.41788	16.61867	45
TC020000C	Plxdc2	NM_02616	"Mus musc	RefSeq	10.47682	6.430157	16.52599	46

TC1600000	Abi3bp	NM_00101	"Mus musc	RefSeq	9.904744	5.889967	16.16472	47
TC1900000	Ms4a4d	NM_02565	"Mus musc	RefSeq	9.778174	5.784103	15.93438	48
TC0200000	Itih5	NM_17247	"Mus musc	RefSeq	8.905849	4.913481	15.91558	49
TC0X00002	Gabra3	NM_00806	"Mus musc	RefSeq	8.10325	4.126899	15.73986	50
TC1700001	Thbs2	NM_01158	"Mus musc	RefSeq	9.59001	5.632303	15.53776	51
TC1300000	Ogn	NM_00876	"Mus musc	RefSeq	10.94485	7.002756	15.37055	52
TC0300001	F3	NM_01017	"Mus musc	RefSeq	7.41203	3.481693	15.24577	53
TC1000003	Lrp1	NM_00851	"Mus musc	RefSeq	10.92542	6.996996	15.22554	54
TC1700000	Smoc2	NM_02231	"Mus musc	RefSeq	9.664726	5.738447	15.20295	55
TC0100002	Serpine2	NM_00925	"Mus musc	RefSeq	8.69521	4.770588	15.1855	56
TC0100000	Col3a1	NM_00993	"Mus musc	RefSeq	10.66913	6.746287	15.16673	57
TC0100003	Ddr2	NM_02256	"Mus musc	RefSeq	9.475402	5.559481	15.09419	58
TC0900000	Cilp	NM_17338	"Mus musc	RefSeq	8.735873	4.828939	15.00045	59
TC1700001	Qpct	NM_02745	"Mus musc	RefSeq	8.233848	4.332274	14.94482	60
TC0100002	Cdh19	NM_00108	"Mus musc	RefSeq	7.153038	3.254796	14.91035	61
TC0800001	Crispld2	NM_03020	"Mus musc	RefSeq	9.728717	5.837375	14.83921	62
TC0100001	Olfml2b	NM_17706	"Mus musc	RefSeq	9.162374	5.27294	14.81959	63
TC0200003	Frzb	NM_01135	"Mus musc	RefSeq	8.430536	4.542925	14.80088	64
TC1900000	Cd248	NM_05404	"Mus musc	RefSeq	10.50761	6.643854	14.55811	65
TC1000001	Enpp1	NM_00881	"Mus musc	RefSeq	7.886095	4.031273	14.46829	66
TC0100000	Efhd1	NM_02888	"Mus musc	RefSeq	8.314173	4.464009	14.42165	67
TC0300000	Hmgcs2	NM_00825	"Mus musc	RefSeq	8.981927	5.139481	14.3447	68
TC1900000	Aldh1a1	NM_01346	"Mus musc	RefSeq	8.926653	5.092446	14.26301	69
TC1300000	Aspn	NM_02571	"Mus musc	RefSeq	9.664908	5.854146	14.0331	70
TC0100003	Pla2g4a	NM_00886	"Mus musc	RefSeq	7.527216	3.72077	13.99118	71
TC0700003	Anpep	NM_00848	"Mus musc	RefSeq	9.503523	5.722171	13.74993	72
TC0600000	Col1a2	NM_00774	"Mus musc	RefSeq	10.26409	6.485061	13.72782	73
TC0300000	Mme	NM_00128	"membrane	RefSeq	7.282216	3.516938	13.59758	74
TC0500002	Htra3	NM_03012	"Mus musc	RefSeq	10.91548	7.163639	13.47153	75
TC0200001	Thbs1	NM_01158	"Mus musc	RefSeq	10.13719	6.392996	13.40028	76
TC1500000	Sdc2	NM_00830	"Mus musc	RefSeq	9.512215	5.77323	13.35201	77
TC0800001	Htra4	NM_00108	"Mus musc	RefSeq	8.986083	5.251083	13.31518	78
TC0600001	C1rb	NM_00111	"Mus musc	RefSeq	9.115675	5.382723	13.29629	79
TC0400003	Pdpn	NM_01032	"Mus musc	RefSeq	8.393104	4.665215	13.24971	80
TC0900001	Naalad2	NM_02827	"Mus musc	RefSeq	8.114182	4.390116	13.21465	81
TC0900001	Tbx20	NM_19426	"Mus musc	RefSeq	10.34843	6.637087	13.09866	82
TC1500000	Matn2	NM_01676	"Mus musc	RefSeq	8.914239	5.2032	13.09586	83
TC1000002	Bicc1	NM_03139	"Mus musc	RefSeq	9.478445	5.768165	13.08897	84
TC0800001	Mt2	NM_00863	"Mus musc	RefSeq	11.45462	7.74728	13.06233	85
TC0600003	C1s	NM_14493	"Mus musc	RefSeq	8.884859	5.194334	12.91097	86
TC0400000	Gm13301	ENSMUST0	cdna:know	ENSEMBL	7.442954	3.757826	12.86276	87
TC1400001	Bmpr1a	NM_00975	"Mus musc	RefSeq	8.27183	4.58995	12.83383	88
TC1000001	Dse	NM_17250	"Mus musc	RefSeq	8.221529	4.566284	12.59907	89
TC0500000	Pdgfra	NM_01105	"Mus musc	RefSeq	10.44936	6.796767	12.57596	90
TC0200001	Fam171b	NM_17551	"Mus musc	RefSeq	7.122452	3.469879	12.57575	91
TC0700004	Nupr1	NM_01973	"Mus musc	RefSeq	8.234543	4.583322	12.56397	92
TC0100001	Dpt	NM_01975	"Mus musc	RefSeq	11.80672	8.159849	12.52614	93

TC0300000	Cp	NM_00775	"Mus musc	RefSeq	8.62971	4.992443	12.44304	94
TC0900002	Oaf	NM_17864	"Mus musc	RefSeq	8.61009	4.979676	12.38407	95
TC1900001	Scd1	NM_00912	"Mus musc	RefSeq	10.08092	6.453141	12.36143	96
TC1100001	Ccl2	NM_01133	"Mus musc	RefSeq	8.301573	4.675707	12.3451	97
TC1700001	Fndc1	NM_00108	"Mus musc	RefSeq	8.464807	4.842224	12.31703	98
TC0700002	Axl	NM_00946	"Mus musc	RefSeq	10.82563	7.250443	11.91895	99
TC1400000	Ebf2	NM_01009	"Mus musc	RefSeq	8.910652	5.338856	11.89098	100
TC0600003	Gm5077	NM_17386	"Mus musc	RefSeq	8.324237	4.768911	11.75601	101
TC0900000	Aldh1a2	NM_00902	"Mus musc	RefSeq	7.838708	4.292782	11.67966	102
TC0500002	Spon2	NM_13390	"Mus musc	RefSeq	8.693323	5.184481	11.38326	103
TC0100002	Irs1	NM_01057	"Mus musc	RefSeq	7.035169	3.526862	11.37904	104
TC1100001	Col1a1	NM_00774	"Mus musc	RefSeq	10.48125	6.978707	11.33367	105
TC0100000	Sulf1	NM_00119	"Mus musc	RefSeq	10.00579	6.504906	11.32064	106
TC0400002	Svep1	NM_02281	"Mus musc	RefSeq	8.040477	4.548647	11.24982	107
TC1500000	Kdelr3	NM_13409	"Mus musc	RefSeq	8.954509	5.467598	11.21153	108
TC0100000	Cyp27a1	NM_02426	"Mus musc	RefSeq	9.098023	5.612763	11.19871	109
TC1900001	Anxa1	NM_01073	"Mus musc	RefSeq	9.581556	6.114292	11.05988	110
TC0300002	Sgms2	NM_02894	"Mus musc	RefSeq	7.801054	4.338976	11.0202	111
TC1100001	Mrc2	NM_00862	"Mus musc	RefSeq	8.926595	5.465722	11.011	112
TC0200003	Rnd3	NM_02881	"Mus musc	RefSeq	8.515618	5.056071	11.00088	113
TC1000002	Col6a1	NM_00993	"Mus musc	RefSeq	10.13523	6.676036	10.99822	114
TC0600001	C1ra	NM_02314	"Mus musc	RefSeq	8.700404	5.24182	10.99354	115
TC0200005	Entpd2	NM_00984	"Mus musc	RefSeq	9.266197	5.845262	10.71036	116
TC0200003	Pde1a	NM_01674	"Mus musc	RefSeq	7.205268	3.789851	10.66947	117
TC0800000	Pdgfrl	NM_02684	"Mus musc	RefSeq	8.004271	4.595367	10.62141	118
TC1100001	Ccl11	NM_01133	"Mus musc	RefSeq	7.789303	4.391236	10.54193	119
TC1400000	Gm21451	XM_00368	"PREDICTEI	RefSeq	9.121288	5.72355	10.53953	120
TC1600000	Vgll3	NM_02857	"Mus musc	RefSeq	7.686231	4.290045	10.52819	121
TC0900001	Clec3b	NM_01160	"Mus musc	RefSeq	8.413428	5.020745	10.50266	122
TC1700000	Pi16	NM_02373	"Mus musc	RefSeq	9.862012	6.474995	10.46149	123
TC1000002	Col6a2	NM_14600	"Mus musc	RefSeq	9.872415	6.487995	10.44268	124
TC1200002	Ifi27l2a	NM_00128	"interferon	RefSeq	9.013762	5.635209	10.4003	125
TC0500000	Fgl2	NM_00801	"Mus musc	RefSeq	8.281307	4.907612	10.36534	126
TC1200000	Osr1	NM_01185	"Mus musc	RefSeq	9.593149	6.220127	10.3605	127
TC0100003	Cfh	NM_00988	"Mus musc	RefSeq	11.01519	7.657831	10.2486	128
TC0500000	Cxcl1	NM_00817	"Mus musc	RefSeq	10.13271	6.776875	10.23779	129
TC0200002	Gm11037	ENSMUST0	cdna:know	ENSEMBL	3.328341	6.679293	-10.2032	130
TC0800000	Fat1	NM_00108	"Mus musc	RefSeq	8.732134	5.385047	10.17592	131
TC1500001	Enpp2	NM_00113	"Mus musc	RefSeq	8.809086	5.472831	10.0998	132
TC0500003	Serpine1	NM_00887	"Mus musc	RefSeq	9.213432	5.881487	10.06967	133
TC0900000	Itga11	NM_17692	"Mus musc	RefSeq	8.521543	5.198336	10.00887	134
TC0X00003	Srpx	NM_01691	"Mus musc	RefSeq	7.284581	3.968616	9.958752	135
TC0200003	Serping1	NM_00977	"Mus musc	RefSeq	11.56924	8.255488	9.943474	136
TC1900000	Il33	NM_13377	"Mus musc	RefSeq	7.540575	4.228603	9.931227	137
TC1100001	Tmem100	NM_02643	"Mus musc	RefSeq	7.97942	4.674391	9.883548	138
TC1500000	Fbln1	NM_01018	"Mus musc	RefSeq	10.3249	7.027284	9.83288	139
TC0300000	Lhfp	NM_17538	"Mus musc	RefSeq	9.766135	6.485227	9.719674	140

TC0X00002	Itm2a	NM_00840	"Mus musc	RefSeq	9.056317	5.780612	9.684684	141
TC0300001	Ngf	NM_00111	"Mus musc	RefSeq	9.525289	6.255459	9.645326	142
TC0400004	Podn	NM_00128	"podocan (	RefSeq	9.355919	6.098609	9.561984	143
TC0900003	Rbms3	NM_00117	"Mus musc	RefSeq	8.368533	5.119785	9.505404	144
TC0700002	Rcn3	NM_02655	"Mus musc	RefSeq	10.10184	6.86143	9.450607	145
TC1300002	Gpx8	NM_02712	"Mus musc	RefSeq	8.675526	5.443545	9.395572	146
TC0500000	C1qtnf7	NM_00113	"Mus musc	RefSeq	7.81871	4.592763	9.356358	147
TC1600000	Ccdc80	NM_02643	"Mus musc	RefSeq	10.42763	7.215215	9.269021	148
TC1400001	Gpc6	NM_01182	"Mus musc	RefSeq	8.834048	5.624593	9.25001	149
TC1600001	Tmem45a	NM_01963	"Mus musc	RefSeq	6.930958	3.724938	9.228013	150
TC0100002	Col6a3	NM_00124	"Mus musc	RefSeq	8.728032	5.522352	9.225838	151
TC0100003	Prelp	NM_05407	"Mus musc	RefSeq	11.62415	8.420704	9.211582	152
TC0100000	Npas2	NM_00871	"Mus musc	RefSeq	8.85097	5.653696	9.172239	153
TC1100000	Slit3	NM_01141	"Mus musc	RefSeq	8.747743	5.55835	9.122271	154
TC0200000	Hmcn2	XM_00308	"PREDICTEI	RefSeq	8.714573	5.539207	9.034007	155
TC0300001	Pcdh18	NM_13044	"Mus musc	RefSeq	8.040283	4.868405	9.012192	156
TC0900000	Mmp3	NM_01080	"Mus musc	RefSeq	7.09035	3.926528	8.962008	157
TC1100001	Sox9	NM_01144	"Mus musc	RefSeq	8.875647	5.713259	8.953104	158
TC0900001	Arhgap42	NM_02782	"Mus musc	RefSeq	7.14341	3.985138	8.927598	159
TC1000001	Ntn4	NM_02132	"Mus musc	RefSeq	8.351673	5.194201	8.922648	160
TC0X00001	Maob	NM_17277	"Mus musc	RefSeq	7.484751	4.327865	8.919025	161
TC0200002	Lbp	NM_00848	"Mus musc	RefSeq	8.782724	5.6293	8.897648	162
TC1100000	Mfap4	NM_02956	"Mus musc	RefSeq	10.17552	7.027116	8.866741	163
TC0700002	Lsp1	NM_00113	"Mus musc	RefSeq	8.585427	5.43885	8.85552	164
TC1100000	Adamts2	NM_00127	"Mus musc	RefSeq	9.47055	6.325478	8.846287	165
TC0600003	Mgp	NM_00859	"Mus musc	RefSeq	11.62241	8.492051	8.756547	166
TC1800000	Colec12	NM_13044	"Mus musc	RefSeq	9.216483	6.091834	8.72194	167
TC0400000	Gem	NM_01027	"Mus musc	RefSeq	9.583283	6.469181	8.658409	168
TC1900001	Myof	NM_00109	"Mus musc	RefSeq	7.554644	4.458858	8.54918	169
TC1000002	Sobp	NM_17540	"Mus musc	RefSeq	7.621814	4.528008	8.537455	170
TC0500000	Sema3d	NM_02888	"Mus musc	RefSeq	6.869658	3.780823	8.508088	171
TC4_GL456	LOC10105€	ENSMUST0	cdna:know	ENSEMBL	7.889756	4.810019	8.454603	172
TC0900001	Rbp1	NM_01125	"Mus musc	RefSeq	10.18989	7.112438	8.441246	173
TC0400004	Angptl7	NM_00103	"Mus musc	RefSeq	7.891155	4.815876	8.428518	174
TC0400001	Lepr	NM_01070	"Mus musc	RefSeq	6.568073	3.505842	8.352633	175
TC0100003	Cd55	NM_01001	"Mus musc	RefSeq	10.59139	7.540923	8.284818	176
TC0X00000	Ar	NM_01347	"Mus musc	RefSeq	8.441268	5.394408	8.264113	177
TC0100001	Plekha6	NM_00116	"Mus musc	RefSeq	8.51324	5.475949	8.209481	178
TC0300000	S100a6	NM_01131	"Mus musc	RefSeq	8.779554	5.743327	8.203429	179
TC0X00001	Gpr64	NM_17871	"Mus musc	RefSeq	6.255924	3.223976	8.179133	180
TC0600000	Akr1b8	NM_00801	"Mus musc	RefSeq	7.138819	4.116126	8.126832	181
TC1000001	Rassf9	NM_14624	"Mus musc	RefSeq	4.750019	7.771024	-8.11733	182
TC0200004	Cpxm1	NM_01969	"Mus musc	RefSeq	8.109875	5.095996	8.077333	183
TC0200004	Fibin	NM_02627	"Mus musc	RefSeq	7.626641	4.612943	8.07632	184
TC1500000	Lgals1	NM_00849	"Mus musc	RefSeq	9.87574	6.869423	8.035106	185
TC1700002	Cyp1b1	NM_00999	"Mus musc	RefSeq	7.207875	4.206299	8.008744	186
TC1100002	Olfr1396	NM_14633	"Mus musc	RefSeq	5.33476	8.333995	-7.99576	187

TC0700003	Emp3	NM_00114	"Mus musc	RefSeq	10.71926	7.732801	7.925253	188
TC1600000	Fstl1	NM_00804	"Mus musc	RefSeq	10.82415	7.840875	7.90777	189
TC0700001	Adamtsl3	NM_00119	"Mus musc	RefSeq	8.662101	5.679057	7.906526	190
TC4_JH584	Gm3893	ENSMUST0	cdna:know	ENSEMBL	8.018448	5.059528	7.775417	191
TC0400002	Gpr153	NM_17840	"Mus musc	RefSeq	8.704016	5.748123	7.75912	192
TC0900000	Cdon	NM_02133	"Mus musc	RefSeq	8.423744	5.469731	7.749015	193
TC0600002	Ret	NM_00905	"Mus musc	RefSeq	8.585426	5.633156	7.739659	194
TC1400001	Pcdh17	NM_00101	"Mus musc	RefSeq	4.794196	7.743738	-7.72504	195
TC0900003	Trf	NM_13397	"Mus musc	RefSeq	8.474835	5.526815	7.716892	196
TC0500000	Pcdh7	NM_01876	"Mus musc	RefSeq	7.160385	4.212835	7.714379	197
TC0600003	Klra9	NM_01065	"Mus musc	RefSeq	3.938719	6.877435	-7.66729	198
TC0100003	Cfhr2	NM_00102	"Mus musc	RefSeq	7.589203	4.65095	7.664826	199
TC0600002	Rarres2	NM_02785	"Mus musc	RefSeq	8.173999	5.238924	7.64796	200
TC1600001	Adamts5	NM_01178	"Mus musc	RefSeq	10.40095	7.468578	7.633644	201
TC0200003	Cd302	NM_02542	"Mus musc	RefSeq	7.980342	5.078479	7.473909	202
TC0400001	Ror1	NM_01384	"Mus musc	RefSeq	7.140919	4.245979	7.43813	203
TC0400003	Dock7	NM_02608	"Mus musc	RefSeq	7.696571	4.804745	7.422093	204
TC1300000	Dapk1	NM_02965	"Mus musc	RefSeq	7.911338	5.019783	7.420699	205
TC0800001	Mt1	NM_01360	"Mus musc	RefSeq	10.84961	7.959576	7.412869	206
TC0800001	Gnao1	NM_00111	"Mus musc	RefSeq	8.32053	5.43083	7.411163	207
TC0600000	Foxp2	NM_05324	"Mus musc	RefSeq	6.693416	3.81116	7.373022	208
TC0300000	Selenbp1	NM_00915	"Mus musc	RefSeq	8.932341	6.052643	7.35996	209
TC1600001	Apod	NM_00747	"Mus musc	RefSeq	7.566026	4.687137	7.355834	210
TC1400000	Ang	NM_00116	"Mus musc	RefSeq	7.966702	5.10433	7.2721	211
TC0700000	Ndn	NM_01088	"Mus musc	RefSeq	8.211223	5.352455	7.253956	212
TC0700000	Clip3	NM_00108	"Mus musc	RefSeq	8.317243	5.465192	7.220261	213
TC1300002	Gas1	NM_00808	"Mus musc	RefSeq	8.862494	6.011951	7.212718	214
TC0300000	Notch2	NM_01092	"Mus musc	RefSeq	8.83836	5.989356	7.205028	215
TC1100004	Timp2	NM_01159	"Mus musc	RefSeq	10.50241	7.660772	7.168325	216
TC1500000	Adamts12	NM_17550	"Mus musc	RefSeq	7.607219	4.781124	7.091521	217
TC0X00001	Gpm6b	NM_00117	"Mus musc	RefSeq	7.981886	5.166526	7.038949	218
TC0100003	Gm4788	NM_00116	"Mus musc	RefSeq	7.578531	4.773894	6.986825	219
TC1500001	Igfbp6	NM_00834	"Mus musc	RefSeq	8.787991	5.991187	6.948993	220
TC0100000	Gsta3	NM_00128	"glutathion	RefSeq	6.993719	4.198233	6.942648	221
TC0700001	Spon1	NM_14558	"Mus musc	RefSeq	8.08727	5.294804	6.92813	222
TC0400000	Reck	NM_01667	"Mus musc	RefSeq	8.663901	5.874462	6.913609	223
TC0900000	Clmp	NM_13373	"Mus musc	RefSeq	7.792749	5.006282	6.899381	224
TC1400000	Mmp14	NM_00860	"Mus musc	RefSeq	9.589367	6.806157	6.883823	225
TC0400002	Mxra8	NM_02426	"Mus musc	RefSeq	9.478023	6.710751	6.808193	226
TC1300002	Cxcl14	NM_01956	"Mus musc	RefSeq	8.25973	5.494244	6.79977	227
TC0100001	Angptl1	NM_02833	"Mus musc	RefSeq	6.692763	3.92985	6.787654	228
TC0300000	Selenbp2	NM_01941	"Mus musc	RefSeq	8.495876	5.736223	6.772333	229
TC0X00002	Gpc3	NM_01669	"Mus musc	RefSeq	7.117006	4.362268	6.749301	230
TC0900000	Pvrl1	NM_02142	"Mus musc	RefSeq	7.715743	4.965884	6.726514	231
TC1400002	Pcdh9	NM_00127	"Mus musc	RefSeq	6.625521	3.887905	6.669673	232
TC1000000	Ctgf	NM_01021	"Mus musc	RefSeq	9.533941	6.801958	6.643682	233
TC1400000	Scara5	NM_00116	"Mus musc	RefSeq	7.821072	5.091396	6.633067	234

TC1500002	Slc38a4	NM_02705	"Mus musc	RefSeq	6.342768	3.61368	6.630364	235
TC1400001	Gm9780	XM_00147	"PREDICTEI	RefSeq	9.866395	7.140517	6.615627	236
TC0900002	Tbx18	NM_02381	"Mus musc	RefSeq	6.309435	3.597277	6.553011	237
TC020000C	Angptl2	NM_01192	"Mus musc	RefSeq	8.712865	6.004557	6.535547	238
TC1000002	Plxnc1	NM_01879	"Mus musc	RefSeq	6.50985	3.801826	6.534261	239
TC0400002	Necab1	NM_17861	"Mus musc	RefSeq	6.466725	3.76002	6.528289	240
TC0200003	Dpp4	NM_01007	"Mus musc	RefSeq	6.074352	3.372178	6.507818	241
TC140000C	Stc1	NM_00928	"Mus musc	RefSeq	4.949274	7.644088	-6.4747	242
TC0600001	Gxylt2	NM_19861	"Mus musc	RefSeq	7.534322	4.85259	6.416257	243
TC0900003	Rtp3	NM_15310	"Mus musc	RefSeq	5.666934	8.325712	-6.31498	244
TC0100001	5430435G2	NM_14550	"Mus musc	RefSeq	6.984352	4.328186	6.303556	245
TC020000C	Olfml2a	NM_17285	"Mus musc	RefSeq	7.827068	5.176101	6.280881	246
TC0100002	St8sia4	NM_00918	"Mus musc	RefSeq	5.484162	8.126376	-6.24289	247
TC010000C	Aox1	NM_00967	"Mus musc	RefSeq	6.754842	4.116419	6.226507	248
TC1100002	Ugp2	NM_13929	"Mus musc	RefSeq	8.462686	5.826824	6.215464	249
TC0200003	Dnm1	NM_01006	"Mus musc	RefSeq	7.900035	5.26477	6.212892	250
TC1400001	Itgbl1	NM_14546	"Mus musc	RefSeq	7.233826	4.598725	6.212186	251
TC0300003	Fabp9	NM_01159	"Mus musc	RefSeq	4.837862	7.472445	-6.20996	252
TC0X00003	Il2rg	NM_01356	"Mus musc	RefSeq	6.311442	8.94463	-6.20395	253
TC090000C	Arhgap20	NM_17553	"Mus musc	RefSeq	6.639917	4.011195	6.184779	254
TC0500002	Ugdh	NM_00946	"Mus musc	RefSeq	9.11746	6.490347	6.177885	255
TC0300003	Ifi44	NM_13387	"Mus musc	RefSeq	6.538617	9.164711	-6.17352	256
TC0200001	Casc4	NM_19903	"Mus musc	RefSeq	7.178049	4.555632	6.157808	257
TC0700004	Dkk3	NM_01581	"Mus musc	RefSeq	8.256362	5.637403	6.143066	258
TC050000C	Lrrc17	NM_02897	"Mus musc	RefSeq	6.875123	4.260432	6.12492	259
TC0400002	AI464131	NM_00108	"Mus musc	RefSeq	6.451979	3.838638	6.119191	260
TC0100002	Dock10	NM_00128	"dedicator	RefSeq	6.431976	3.821392	6.107509	261
TC0X00001	Klhl4	NM_17278	"Mus musc	RefSeq	4.636318	7.246802	-6.10709	262
TC0200002	St8sia6	NM_14583	"Mus musc	RefSeq	5.679417	8.28919	-6.10408	263
TC1600001	Bcl6	NM_00974	"Mus musc	RefSeq	7.909142	5.299457	6.103704	264
TC030000C	Ctsk	NM_00780	"Mus musc	RefSeq	6.645002	4.035665	6.102232	265
TC1000001	Cd63	NM_00104	"Mus musc	RefSeq	9.339638	6.731225	6.098325	266
TC010000C	Twist2	NM_00785	"Mus musc	RefSeq	7.266052	4.658856	6.093183	267
TC020000C	Adamtsl2	NM_02998	"Mus musc	RefSeq	8.238219	5.634318	6.079282	268
TC140000C	Mmrn2	NM_15312	"Mus musc	RefSeq	7.058173	9.652641	-6.03966	269
TC1100001	Igfbp4	NM_01051	"Mus musc	RefSeq	10.16958	7.576681	6.03311	270
TC1100003	Scpep1	NM_02902	"Mus musc	RefSeq	9.020447	6.428074	6.030899	271
TC1000001	Mmp19	NM_00116	"Mus musc	RefSeq	7.11175	4.522277	6.018788	272
TC1200002	Pgf	NM_00127	"Mus musc	RefSeq	8.653151	6.064721	6.014438	273
TC1300001	Gm5454	XM_48431	"PREDICTEI	RefSeq	7.861925	5.277497	5.997777	274
TC0100002	Col5a2	NM_00773	"Mus musc	RefSeq	7.956141	5.37255	5.994299	275
TC160000C	Robo1	NM_01941	"Mus musc	RefSeq	6.861347	4.279741	5.986057	276
TC050000C	Cd38	NM_00764	"Mus musc	RefSeq	6.702959	9.282151	-5.97605	277
TC1500001	Has2	NM_00821	"Mus musc	RefSeq	7.697036	5.118077	5.975084	278
TC0100001	Pydc3	NM_00116	"Mus musc	RefSeq	4.57571	7.152436	-5.96584	279
TC0600002	Inmt	NM_00934	"Mus musc	RefSeq	6.670563	4.094333	5.963792	280
TC0X0000C	Timp1	NM_00104	"Mus musc	RefSeq	7.173044	4.614814	5.889846	281

TC0800001	Adcy7	NM_00740	"Mus musc	RefSeq	7.797838	5.241893	5.880525	282
TC180000C	Tubb6	NM_02647	"Mus musc	RefSeq	9.279799	6.723945	5.880154	283
TC1300002	Ctla2b	NM_00779	"Mus musc	RefSeq	5.12239	7.676302	-5.87224	284
TC0800002	Tenm3	NM_00114	"Mus musc	RefSeq	7.094586	4.541129	5.870393	285
TC1100003	Serpinf1	NM_01134	"Mus musc	RefSeq	9.02882	6.475879	5.868293	286
TC0600002	Hk2	NM_01382	"Mus musc	RefSeq	7.392107	4.842179	5.856051	287
TC0400004	Rbp7	NM_02202	"Mus musc	RefSeq	6.692603	9.239593	-5.84414	288
TC0800002	Tmem184c	NM_14559	"Mus musc	RefSeq	8.305362	5.758456	5.843797	289
TC0100003	Cxcr4	NM_00991	"Mus musc	RefSeq	6.319406	8.86587	-5.84201	290
TC110000C	Aebp1	NM_00963	"Mus musc	RefSeq	8.75504	6.216031	5.811896	291
TC1300002	Rasgrf2	NM_00902	"Mus musc	RefSeq	6.107127	8.643605	-5.80171	292
TC0600001	8430408G2	NM_00116	"Mus musc	RefSeq	7.693295	10.22383	-5.77785	293
TC0X0000C	Dock11	NM_00100	"Mus musc	RefSeq	7.210016	4.684109	5.759354	294
TC050000C	Slit2	NM_17880	"Mus musc	RefSeq	6.161769	3.637581	5.752496	295
TC120000C	Fam49a	NM_00114	"Mus musc	RefSeq	8.298017	5.775265	5.746773	296
TC030000C	Tnfsf10	NM_00942	"Mus musc	RefSeq	6.048827	8.565086	-5.72097	297
TC060000C	Gimap4	NM_17499	"Mus musc	RefSeq	7.039266	9.547847	-5.6906	298
TC0500002	Cxcl9	NM_00859	"Mus musc	RefSeq	6.518565	9.026658	-5.68868	299
TC0100002	Aff3	NM_01067	"Mus musc	RefSeq	7.184222	4.677936	5.681556	300
TC0200003	Fap	NM_00798	"Mus musc	RefSeq	6.657086	4.156996	5.657207	301
TC0800001	Rhou	NM_13395	"Mus musc	RefSeq	7.908368	5.409782	5.651313	302
TC1000002	Aim1	NM_17239	"Mus musc	RefSeq	4.839577	7.33352	-5.63315	303
TC1900001	Dtx4	NM_17244	"Mus musc	RefSeq	8.021329	5.535052	5.603301	304
TC0500002	Antxr2	NM_13373	"Mus musc	RefSeq	8.908144	6.429128	5.575171	305
TC0700002	Fxyd1	NM_05299	"Mus musc	RefSeq	10.3192	7.841011	5.571984	306
TC0500003	Pcolce	NM_00878	"Mus musc	RefSeq	8.889259	6.412122	5.567914	307
TC1400002	Gm3629	ENSMUST0	cdna:putat	ENSEMBL	5.818052	8.29154	-5.55385	308
TC1500001	Angpt1	NM_00964	"Mus musc	RefSeq	5.805788	3.333603	5.548835	309
TC150000C	Cpq	NM_17607	"Mus musc	RefSeq	7.712195	5.243253	5.536376	310
TC010000C	Tmem182	NM_00108	"Mus musc	RefSeq	5.620164	8.086462	-5.52624	311
TC0600002	Gimap6	NM_15317	"Mus musc	RefSeq	6.608638	9.069721	-5.5063	312
TC130000C	Gli3	NM_00813	"Mus musc	RefSeq	6.92544	4.471078	5.480707	313
TC1200002	Itgb8	NM_17729	"Mus musc	RefSeq	6.13986	3.686541	5.476746	314
TC030000C	Thbs3	NM_01369	"Mus musc	RefSeq	7.344911	4.891633	5.47659	315
TC1600001	Col8a1	NM_00773	"Mus musc	RefSeq	6.997908	4.552037	5.448545	316
TC0600003	Clec1a	NM_17552	"Mus musc	RefSeq	6.579737	9.024122	-5.44294	317
TC0700001	Vwa3a	NM_17769	"Mus musc	RefSeq	6.319259	8.762451	-5.43844	318
TC1700001	Galm	NM_17696	"Mus musc	RefSeq	6.196969	3.756209	5.429277	319
TC0200001	Pamr1	NM_17374	"Mus musc	RefSeq	6.79809	4.361863	5.412244	320
TC1200001	Ahr	NM_01346	"Mus musc	RefSeq	6.256998	8.687344	-5.39023	321
TC1000001	Dcn	NM_00119	"Mus musc	RefSeq	11.57491	9.146332	5.383629	322
TC1400002	Gm8281	ENSMUST0	cdna:putat	ENSEMBL	5.247974	7.667814	-5.35112	323
TC1400002	Gm10340	ENSMUST0	cdna:putat	ENSEMBL	5.958035	8.377874	-5.35111	324
TC120000C	Wdr35	NM_00115	"Mus musc	RefSeq	5.954505	3.536796	5.343218	325
TC070000C	Ppp1r14a	NM_02673	"Mus musc	RefSeq	8.446202	6.03822	5.307314	326
TC0300001	Gstm5	NM_01036	"Mus musc	RefSeq	7.140906	4.736692	5.293471	327
TC0100003	Atp1a2	NM_17840	"Mus musc	RefSeq	8.66713	6.263457	5.291486	328

TC1700001	Vit	NM_02881	"Mus musc	RefSeq	6.937416	4.534121	5.2901	329
TC1600001	Ppl	NM_00890	"Mus musc	RefSeq	7.592842	5.190241	5.287556	330
TC0100003	Prrx1	NM_17568	"Mus musc	RefSeq	6.322165	3.920353	5.284665	331
TC1000002	Nav3	NM_00108	"Mus musc	RefSeq	6.019067	8.420375	-5.28282	332
TC1300002	Ctla2a	NM_00114	"Mus musc	RefSeq	5.645649	8.0465	-5.28115	333
TC140000C	Gm3317	NM_00124	"Mus musc	RefSeq	5.570532	7.966564	-5.26354	334
TC1400002	Gm3752	ENSMUST0	cdna:putat	ENSEMBL	5.63594	8.031971	-5.26353	335
TC1400002	Dach1	NM_00782	"Mus musc	RefSeq	6.210407	8.605601	-5.26048	336
TC0600003	Art4	NM_02663	"Mus musc	RefSeq	6.680746	4.286798	5.255937	337
TC070000C	Atp10a	NM_00972	"Mus musc	RefSeq	7.114139	4.721299	5.251902	338
TC100000C	Myct1	NM_02679	"Mus musc	RefSeq	7.104264	9.495381	-5.24563	339
TC130000C	Gcnt2	NM_02388	"Mus musc	RefSeq	5.249346	7.63932	-5.24148	340
TC0600002	Scrn1	NM_02726	"Mus musc	RefSeq	7.687758	5.298657	5.238308	341
TC1400001	Plac9a	NM_20722	"Mus musc	RefSeq	9.551528	7.164239	5.231733	342
TC1400002	Ednrb	NM_00127	"Mus musc	RefSeq	6.959049	9.345047	-5.22705	343
TC1400002	Gm3173	ENSMUST0	cdna:putat	ENSEMBL	5.975898	8.360011	-5.22023	344
TC0500002	Ldb2	NM_01069	"Mus musc	RefSeq	6.622529	9.006046	-5.21807	345
TC0600001	Apold1	NM_00110	"Mus musc	RefSeq	6.778768	9.161199	-5.21415	346
TC050000C	Abcb1a	NM_01107	"Mus musc	RefSeq	7.39314	9.77507	-5.21234	347
TC1300001	Zfp366	NM_00100	"Mus musc	RefSeq	7.969911	10.34586	-5.19079	348
TC0500001	Fam20c	NM_03056	"Mus musc	RefSeq	7.54452	5.169236	5.188379	349
TC070000C	Cacng7	NM_13318	"Mus musc	RefSeq	8.230003	5.855431	5.18582	350
TC1800001	Setbp1	NM_05309	"Mus musc	RefSeq	7.654639	5.283611	5.173096	351
TC1600001	AU021092	NM_00103	"Mus musc	RefSeq	5.810475	8.181051	-5.17148	352
TC0600001	Pde3a	NM_01877	"Mus musc	RefSeq	7.702798	5.333324	5.167527	353
TC140000C	Gm3043	XM_00147	"PREDICTEI	RefSeq	4.246931	6.612883	-5.15493	354
TC030000C	Ptx3	NM_00898	"Mus musc	RefSeq	7.194561	4.836657	5.126251	355
TC1200002	LOC100047	XM_00368	"PREDICTEI	MGI/Jacksc	8.209736	5.854069	5.118308	356
TC090000C	Sesn3	NM_03026	"Mus musc	RefSeq	7.766145	5.410841	5.11702	357
TC1100002	Tgtp2	NM_00114	"Mus musc	RefSeq	7.442976	9.797544	-5.11441	358
TC1400002	Gm3005	ENSMUST0	cdna:putat	ENSEMBL	6.338326	8.691387	-5.10907	359
TC080000C	Fgfr1	NM_00107	"Mus musc	RefSeq	9.255914	6.905916	5.098235	360
TC0700004	Chst15	NM_02993	"Mus musc	RefSeq	6.096837	8.44645	-5.09688	361
TC0100001	F11r	NM_17264	"Mus musc	RefSeq	6.796703	9.145657	-5.09455	362
TC1700001	Rasgrp3	NM_00116	"Mus musc	RefSeq	8.105174	10.4536	-5.09269	363
TC1100001	Slfn3	NM_01140	"Mus musc	RefSeq	5.949217	8.29473	-5.08241	364
TC0600001	Pparg	NM_00112	"Mus musc	RefSeq	6.612323	8.955343	-5.07364	365
TC1100003	Alox12	NM_00744	"Mus musc	RefSeq	5.695808	8.036424	-5.06519	366
TC190000C	Cd274	NM_02189	"Mus musc	RefSeq	6.545994	8.886319	-5.06417	367
TC0300003	St6galnac3	NM_01137	"Mus musc	RefSeq	5.613388	7.952325	-5.0593	368
TC1100002	Btnl9	NM_17279	"Mus musc	RefSeq	7.837184	10.17469	-5.05426	369
TC0900002	Pknox2	NM_00102	"Mus musc	RefSeq	6.841389	4.506081	5.046587	370
TC0300002	Ecm1	NM_00125	"Mus musc	RefSeq	9.184268	6.850626	5.040763	371
TC1400002	Gm10340	XM_00368	"PREDICTEI	RefSeq	6.411445	8.744834	-5.03988	372
TC1400002	Gm2974	ENSMUST0	cdna:putat	ENSEMBL	5.787486	8.120875	-5.03988	373
TC0300002	Palmd	NM_02324	"Mus musc	RefSeq	7.105933	9.438363	-5.03653	374
TC020000C	Gsn	NM_00120	"Mus musc	RefSeq	11.82764	9.496329	5.032635	375

TC0800000	Tmem255b	NM_00114	"Mus musc	RefSeq	7.463867	9.793257	-5.02593	376
TC0200001	Slc28a2	NM_17298	"Mus musc	RefSeq	7.47872	9.807062	-5.02228	377
TC1400000	Gm3239	ENSMUST0	cdna:putat	ENSEMBL	5.873667	8.200581	-5.01731	378
TC1400000	D830030K2	NM_17713	"Mus musc	RefSeq	3.909884	6.236797	-5.01731	379
TC1000003	Slc26a10	NM_17761	"Mus musc	RefSeq	8.061352	10.38584	-5.00888	380
TC0300000	Dclk1	NM_00111	"Mus musc	RefSeq	6.421795	4.09853	5.004636	381
TC1600000	Dcbld2	NM_02852	"Mus musc	RefSeq	7.376999	5.053872	5.004157	382
TC0200004	Fbn1	NM_00799	"Mus musc	RefSeq	10.65222	8.331983	4.994149	383
TC1400002	Gm3591	ENSMUST0	cdna:putat	ENSEMBL	6.290323	8.609921	-4.99193	384
TC0500003	Oas2	NM_14522	"Mus musc	RefSeq	6.506766	8.823367	-4.98157	385
TC1400001	Gm10128	ENSMUST0	cdna:know	ENSEMBL	6.009912	8.325998	-4.97979	386
TC1600000	Cd80	NM_00985	"Mus musc	RefSeq	5.946187	3.631402	4.975305	387
TC1000001	Kitl	NM_01359	"Mus musc	RefSeq	7.9084	10.22184	-4.97068	388
TC0200002	Plcb1	NM_01967	"Mus musc	RefSeq	6.403466	8.716448	-4.96909	389
TC0200001	Aplnr	NM_01178	"Mus musc	RefSeq	7.072255	9.384367	-4.9661	390
TC1900001	Fads2	NM_01969	"Mus musc	RefSeq	7.984712	5.675698	4.955443	391
TC1000001	Fam26e	NM_17890	"Mus musc	RefSeq	6.02865	3.723716	4.941448	392
TC1400000	Gm3494	ENSMUST0	cdna:putat	ENSEMBL	5.980015	8.280418	-4.92595	393
TC0300002	Kirrel	NM_00117	"Mus musc	RefSeq	7.734817	5.43499	4.923987	394
TC1400001	Plac9b	NM_00127	"Mus musc	RefSeq	9.263497	6.969829	4.903011	395
TC1400002	Gm3642	XM_00368	"PREDICTEI	RefSeq	5.040403	7.333883	-4.90237	396
TC0200004	Cd93	NM_01074	"Mus musc	RefSeq	7.998935	10.29145	-4.89908	397
TC0800001	Efnb2	NM_01011	"Mus musc	RefSeq	7.887359	10.17802	-4.89282	398
TC0200004	Ptprj	NM_00113	"Mus musc	RefSeq	7.713491	5.423198	4.891554	399
TC1200002	Tgfb3	NM_00936	"Mus musc	RefSeq	8.80675	6.518585	4.884345	400
TC1000001	Syne1	NM_15339	"Mus musc	RefSeq	6.850823	9.137815	-4.88038	401
TC1200001	Rsad2	NM_02138	"Mus musc	RefSeq	6.72168	9.007502	-4.87642	402
TC0300002	Gm4392	XM_00148	"PREDICTEI	RefSeq	6.853548	4.570887	4.865746	403
TC1400000	D830030K2	NM_17713	"Mus musc	RefSeq	4.653882	6.931484	-4.84871	404
TC1400002	Gm3636	XM_00368	"PREDICTEI	RefSeq	5.992423	8.26801	-4.84195	405
TC0600000	Cav2	NM_00127	"Mus musc	RefSeq	7.232609	9.503983	-4.82783	406
TC0200000	Col5a1	NM_01573	"Mus musc	RefSeq	8.268698	5.999951	4.819044	407
TC1400000	Gm10408	NM_00125	"Mus musc	RefSeq	4.014044	6.279572	-4.8083	408
TC0X00001	4930578C1	NM_17522	"Mus musc	RefSeq	5.598751	7.862141	-4.80118	409
TC0500002	Naaa	NM_00116	"Mus musc	RefSeq	7.070055	4.809065	4.793203	410
TC0700002	Ceacam1	NM_00103	"Mus musc	RefSeq	7.839518	10.09857	-4.78678	411
TC0300001	Igsf3	NM_20720	"Mus musc	RefSeq	7.706697	5.45129	4.77469	412
TC0500000	Cacna2d1	NM_00111	"Mus musc	RefSeq	6.101176	3.847089	4.770323	413
TC0600003	Klra10	NM_00845	"Mus musc	RefSeq	3.748872	5.994742	-4.74323	414
TC0900001	Gm11114	ENSMUST0	cdna:know	ENSEMBL	4.675664	6.919006	-4.73493	415
TC1000000	Nepn	NM_02568	"Mus musc	RefSeq	4.374456	6.617516	-4.734	416
TC1400001	Lmo7	NM_20152	"Mus musc	RefSeq	5.370317	7.612714	-4.73183	417
TC1500000	Myc	NM_00117	"Mus musc	RefSeq	8.190085	5.949028	4.727433	418
TC1200001	Efr3b	NM_00108	"Mus musc	RefSeq	6.633327	8.872323	-4.72068	419
TC0200002	Celf2	NM_00111	"Mus musc	RefSeq	6.258338	4.019914	4.718813	420
TC0300000	Stmn2	NM_02528	"Mus musc	RefSeq	6.54791	8.785783	-4.71701	421
TC0300002	Vcam1	NM_01169	"Mus musc	RefSeq	8.811947	6.576689	4.708469	422

TC1500000	Parvb	NM_13316	"Mus musc	RefSeq	6.011881	8.245749	-4.70394	423
TC0200005	Ptgis	NM_00896	"Mus musc	RefSeq	8.01426	5.781367	4.700757	424
TC1900000	Ltbp3	NM_00852	"Mus musc	RefSeq	8.398299	6.16564	4.699994	425
TC0200001	Gm14085	NM_00108	"Mus musc	RefSeq	6.051926	8.284423	-4.69947	426
TC1400002	LOC100861	NM_00127	"Mus musc	RefSeq	5.777152	8.004397	-4.68239	427
TC0900000	Robo4	NM_02878	"Mus musc	RefSeq	7.731835	9.958856	-4.68166	428
TC0700003	Gm4070	NM_00124	"Mus musc	RefSeq	6.921353	9.148296	-4.68141	429
TC1400002	Gm3095	XM_00308	"PREDICTEI	RefSeq	6.016874	8.241636	-4.67434	430
TC1800001	Pcdh12	NM_01737	"Mus musc	RefSeq	6.371642	8.593444	-4.66476	431
TC1100003	Pitpnc1	NM_14582	"Mus musc	RefSeq	6.498242	8.719743	-4.66378	432
TC1700001	Has1	NM_00821	"Mus musc	RefSeq	7.244857	5.023632	4.662892	433
TC0200001	Dll4	NM_01945	"Mus musc	RefSeq	7.703525	9.922075	-4.65425	434
TC1500000	Zfpm2	NM_01176	"Mus musc	RefSeq	7.411306	5.192778	4.654183	435
TC1800001	Arap3	NM_13920	"Mus musc	RefSeq	7.234087	9.452285	-4.65312	436
TC1600002	Erg	NM_13365	"Mus musc	RefSeq	6.686506	8.903556	-4.64942	437
TC0100003	Uap1	NM_13380	"Mus musc	RefSeq	8.870206	6.653247	4.649124	438
TC1900000	Scd2	NM_00912	"Mus musc	RefSeq	9.598916	7.385624	4.637322	439
TC1200001	Nova1	NM_02136	"Mus musc	RefSeq	6.438579	4.228875	4.625804	440
TC1000002	Tmtc2	NM_17736	"Mus musc	RefSeq	4.92179	7.131215	-4.62491	441
TC0800001	Irs2	NM_00108	"Mus musc	RefSeq	9.497908	7.289757	4.620827	442
TC1700002	Twsg1	NM_02305	"Mus musc	RefSeq	9.710228	7.503931	4.614892	443
TC1200000	Etv1	NM_00796	"Mus musc	RefSeq	6.808794	4.603095	4.61298	444
TC1800001	Ecscr	NM_00103	"Mus musc	RefSeq	7.370599	9.574756	-4.60805	445
TC1400001	Gm2237	ENSMUST0	cdna:putat	ENSEMBL	5.397391	7.601237	-4.60706	446
TC0100002	1700019D(	NM_14495	"Mus musc	RefSeq	6.454549	4.250864	4.606545	447
TC1400002	Gm3264	NM_00124	"Mus musc	RefSeq	6.474144	8.676822	-4.60333	448
TC1400002	Gm3739	ENSMUST0	cdna:putat	ENSEMBL	5.29584	7.497301	-4.59945	449
TC0400000	Col15a1	NM_00992	"Mus musc	RefSeq	10.13786	7.937065	4.597313	450
TC0800001	Cdh13	NM_01970	"Mus musc	RefSeq	6.819355	9.019755	-4.59607	451
TC1200000	Dact1	NM_00119	"Mus musc	RefSeq	8.642095	6.443141	4.591463	452
TC0200004	Mall	NM_14553	"Mus musc	RefSeq	6.338498	8.535937	-4.58664	453
TC0100003	Daf2	NM_00782	"Mus musc	RefSeq	6.673442	4.479252	4.576327	454
TC1000001	Igf1	NM_01051	"Mus musc	RefSeq	6.553955	4.359923	4.575825	455
TC0900002	Smad3	NM_01676	"Mus musc	RefSeq	7.828316	5.635436	4.572173	456
TC0500000	Nos3	NM_00871	"Mus musc	RefSeq	7.0479	9.239798	-4.56906	457
TC1500001	Samd12	NM_17722	"Mus musc	RefSeq	4.868389	7.059486	-4.56653	458
TC1100000	Flt4	NM_00802	"Mus musc	RefSeq	6.446761	8.637607	-4.56573	459
TC0200000	Etl4	NM_00108	"Mus musc	RefSeq	6.19645	8.387201	-4.56543	460
TC1500001	Pdgfb	NM_01105	"Mus musc	RefSeq	7.484879	9.675299	-4.56438	461
TC0900001	Sh3bgrl2	NM_17250	"Mus musc	RefSeq	4.774832	6.963656	-4.55934	462
TC1000001	Ust	NM_17738	"Mus musc	RefSeq	6.721352	4.532724	4.558717	463
TC1200000	Prkch	NM_00885	"Mus musc	RefSeq	7.262531	9.451158	-4.55871	464
TC1200000	Frmd6	NM_02812	"Mus musc	RefSeq	8.366083	6.179893	4.55102	465
TC0900001	Plscr2	NM_00888	"Mus musc	RefSeq	4.973009	7.157927	-4.54701	466
TC1100001	Nos2	NM_01092	"Mus musc	RefSeq	5.771758	7.955685	-4.54389	467
TC0400002	Aqp7	NM_00747	"Mus musc	RefSeq	8.615979	10.7964	-4.53285	468
TC1700002	Tubb4a	NM_00945	"Mus musc	RefSeq	8.620494	6.440353	4.531978	469

TC0900003	Cmtm8	NM_02729	"Mus musc	RefSeq	5.691813	7.870738	-4.52816	470
TC0300000	Car13	NM_02449	"Mus musc	RefSeq	6.671566	4.492961	4.527156	471
TC0600002	Timp4	NM_08063	"Mus musc	RefSeq	7.561941	9.740528	-4.5271	472
TC1100001	Cd300lg	NM_02798	"Mus musc	RefSeq	8.186467	10.36476	-4.52618	473
TC0200001	Nostrin	NM_18154	"Mus musc	RefSeq	6.231361	8.408314	-4.52198	474
TC0100001	Sox17	NM_01144	"Mus musc	RefSeq	5.535822	7.711201	-4.51704	475
TC0400003	Ptplad2	NM_02576	"Mus musc	RefSeq	4.922948	7.097896	-4.5157	476
TC0900000	Esam	NM_02710	"Mus musc	RefSeq	7.589701	9.763337	-4.51159	477
TC1700002	Stap2	NM_14593	"Mus musc	RefSeq	6.506559	8.679221	-4.50855	478
TC0900000	Bmper	NM_02847	"Mus musc	RefSeq	6.87749	4.704913	4.50828	479
TC0400002	Gm11787	XM_98433	"PREDICTEI	RefSeq	6.962628	9.133866	-4.5041	480
TC0200001	Sirpa	NM_00117	"Mus musc	RefSeq	7.536576	5.366451	4.500624	481
TC1100003	Arhgef15	NM_17756	"Mus musc	RefSeq	6.977323	9.145655	-4.49503	482
TC1400002	Gm3667	ENSMUST0	cdna:putat	ENSEMBL	6.103836	8.269375	-4.48634	483
TC0400003	Cyp4b1	NM_00782	"Mus musc	RefSeq	7.520871	9.686285	-4.48595	484
TC1100000	Gpx3	NM_00816	"Mus musc	RefSeq	10.32624	8.161088	4.485143	485
TC1400000	Il17d	NM_14583	"Mus musc	RefSeq	7.28192	5.116772	4.485124	486
TC0800001	Gpr56	NM_00119	"Mus musc	RefSeq	8.669345	10.83274	-4.47967	487
TC0300002	S1pr1	NM_00790	"Mus musc	RefSeq	7.740748	9.902854	-4.47568	488
TC0400000	Glipr2	NM_02745	"Mus musc	RefSeq	7.931741	5.772586	4.466532	489
TC1400002	Gm2897	NM_00117	"Mus musc	RefSeq	5.249527	7.408393	-4.46564	490
TC1400002	Gm3696	NM_00102	"Mus musc	RefSeq	4.637654	6.79652	-4.46564	491
TC0700001	Tm6sf1	NM_14537	"Mus musc	RefSeq	5.415636	7.570858	-4.45437	492
TC1000000	Dcbld1	NM_02570	"Mus musc	RefSeq	4.686653	6.840552	-4.45029	493
TC1600001	Cyyr1	NM_14485	"Mus musc	RefSeq	6.774858	8.928576	-4.44973	494
TC1800001	Afap1l1	NM_17892	"Mus musc	RefSeq	7.360503	9.51263	-4.44483	495
TC1400002	Gm3252	ENSMUST0	cdna:putat	ENSEMBL	6.489627	8.640009	-4.43945	496
TC1200001	Rapgef5	NM_17593	"Mus musc	RefSeq	6.943552	9.093857	-4.43922	497
TC0600000	Gimap8	NM_00107	"Mus musc	RefSeq	5.34222	7.490995	-4.43451	498
TC0X00000	Lonrf3	NM_02889	"Mus musc	RefSeq	6.739442	4.591385	4.432305	499
TC0900000	Sema7a	NM_01135	"Mus musc	RefSeq	7.935817	10.0831	-4.42994	500
TC1400002	Gm3173	XM_00308	"PREDICTEI	RefSeq	5.901681	8.048826	-4.4295	501
TC0900002	Me1	NM_00119	"Mus musc	RefSeq	7.227889	5.082836	4.423085	502
TC0900001	Fam198a	NM_17774	"Mus musc	RefSeq	6.408027	4.263944	4.420112	503
TC0700004	Gvin1	NM_00103	"Mus musc	RefSeq	7.589533	9.733027	-4.41831	504
TC1400000	Gm3532	ENSMUST0	cdna:putat	ENSEMBL	4.084225	6.224471	-4.40837	505
TC0400003	Nbl1	NM_00867	"Mus musc	RefSeq	8.277474	6.139621	4.401066	506
TC1700002	Efna5	NM_20765	"Mus musc	RefSeq	6.297813	4.160122	4.400572	507
TC0600000	Dysf	NM_00107	"Mus musc	RefSeq	6.66012	8.793605	-4.38776	508
TC0300001	Emcn	NM_00116	"Mus musc	RefSeq	8.589047	10.72196	-4.38601	509
TC0300001	Negr1	NM_17727	"Mus musc	RefSeq	6.882869	4.753666	4.374757	510
TC0600001	Pon3	NM_17300	"Mus musc	RefSeq	7.526896	5.39824	4.373099	511
TC1000002	Tspan15	NM_19799	"Mus musc	RefSeq	6.350727	8.479347	-4.37299	512
TC0400003	Tie1	NM_01158	"Mus musc	RefSeq	8.192481	10.31926	-4.36742	513
TC1600000	Itgb5	NM_01058	"Mus musc	RefSeq	9.651452	7.526703	4.361272	514
TC0200005	Sox18	NM_00923	"Mus musc	RefSeq	8.037371	10.16181	-4.36034	515
TC0900002	Col6a6	NM_00110	"Mus musc	RefSeq	7.409907	5.289074	4.34945	516

TC0900002	Trpc1	NM_01164	"Mus musc	RefSeq	5.761642	3.641554	4.347205	517
TC0900000	Mcam	NM_02306	"Mus musc	RefSeq	7.71827	9.838311	-4.34706	518
TC1900000	Exoc6	NM_17535	"Mus musc	RefSeq	6.811528	8.929842	-4.34186	519
TC0700000	Ssc5d	NM_17300	"Mus musc	RefSeq	7.923226	5.806853	4.336025	520
TC0900002	Pik3cb	NM_02909	"Mus musc	RefSeq	5.893478	8.009399	-4.33467	521
TC0200002	Ccm2l	NM_14553	"Mus musc	RefSeq	7.247972	9.362985	-4.33194	522
TC1200001	Pqlc3	NM_17257	"Mus musc	RefSeq	7.678662	5.564211	4.330252	523
TC0700001	Nox4	NM_00128	"NADPH ox	RefSeq	5.815526	3.701738	4.328263	524
TC0600000	Gimap9	NM_17496	"Mus musc	RefSeq	3.50301	5.616629	-4.32776	525
TC0600002	Arhgap25	NM_17547	"Mus musc	RefSeq	5.246263	7.359673	-4.32713	526
TC1700001	Epb4.1l3	NM_01381	"Mus musc	RefSeq	6.462853	4.349655	4.326493	527
TC1100002	Sgcd	NM_01189	"Mus musc	RefSeq	5.91327	3.802318	4.319763	528
TC0X00000	Shroom4	NM_00104	"Mus musc	RefSeq	6.905437	9.013782	-4.31196	529
TC0700002	Cyp2e1	NM_02128	"Mus musc	RefSeq	6.149377	4.041717	4.309917	530
TC1400002	Gm3558	NM_00127	"Mus musc	RefSeq	5.917682	8.023103	-4.30323	531
TC1800001	Ablim3	NM_19864	"Mus musc	RefSeq	8.5625	10.66593	-4.2973	532
TC0200001	Rapgef4	NM_00120	"Mus musc	RefSeq	4.893285	6.99194	-4.2831	533
TC1000000	Icosl	NM_01579	"Mus musc	RefSeq	8.036028	5.939163	4.277788	534
TC1100003	Prkca	NM_01110	"Mus musc	RefSeq	7.00029	4.906286	4.269313	535
TC1100003	Fam101b	NM_02965	"Mus musc	RefSeq	8.033611	10.12666	-4.26649	536
TC1500002	Plxnb2	NM_00115	"Mus musc	RefSeq	7.825556	5.735963	4.25628	537
TC0200000	Apbb1ip	NM_01945	"Mus musc	RefSeq	6.292662	4.204355	4.252488	538
TC0200003	Zeb2	NM_00128	"zinc finger	RefSeq	6.845475	4.760655	4.242222	539
TC0300001	Fabp4	NM_02440	"Mus musc	RefSeq	9.288854	11.37322	-4.24087	540
TC1800001	Lox	NM_00128	"lysyl oxida	RefSeq	6.510979	4.427118	4.239403	541
TC0700000	Ptgir	NM_00896	"Mus musc	RefSeq	7.130415	5.047556	4.236459	542
TC0700001	Pde2a	NM_00114	"Mus musc	RefSeq	8.12721	10.20869	-4.23241	543
TC1300001	Txndc5	NM_00128	"thioredoxi	RefSeq	9.270203	7.189489	4.230165	544
TC0700000	Gpr4	NM_17566	"Mus musc	RefSeq	7.189479	9.27013	-4.22998	545
TC0600001	Mgst1	NM_01994	"Mus musc	RefSeq	6.856495	4.776575	4.227838	546
TC1100003	Bcl6b	NM_00752	"Mus musc	RefSeq	6.98597	9.063844	-4.22185	547
TC1100001	Car4	NM_00760	"Mus musc	RefSeq	7.62462	9.700875	-4.21711	548
TC1000003	Rassf3	NM_13895	"Mus musc	RefSeq	7.181829	9.255113	-4.20844	549
TC1200001	Tspan13	NM_02535	"Mus musc	RefSeq	7.919418	9.990036	-4.20067	550
TC1400001	Gm3500	ENSMUST0	cdna:putat	ENSEMBL	5.984351	8.053999	-4.19784	551
TC1400002	Ndrg2	NM_00114	"Mus musc	RefSeq	9.253188	7.184386	4.195381	552
TC1400002	Scara3	NM_17260	"Mus musc	RefSeq	6.855938	4.78721	4.195166	553
TC0400000	Epha7	NM_00112	"Mus musc	RefSeq	5.907887	3.839219	4.194992	554
TC0200001	Trp53i11	NM_00102	"Mus musc	RefSeq	8.063217	10.13088	-4.19206	555
TC1800000	Pdgfrb	NM_00114	"Mus musc	RefSeq	9.599476	7.532246	4.190813	556
TC1700000	Clic5	NM_17262	"Mus musc	RefSeq	8.360705	10.42756	-4.18973	557
TC1400000	C1qtnf9	NM_18317	"Mus musc	RefSeq	7.788089	9.85443	-4.18823	558
TC0800002	Ushbp1	NM_18141	"Mus musc	RefSeq	6.949049	9.011068	-4.1757	559
TC0200002	Ppp1r16b	NM_15308	"Mus musc	RefSeq	6.64345	8.704345	-4.17245	560
TC0400000	Lyn	NM_00111	"Mus musc	RefSeq	6.620972	8.679628	-4.16598	561
TC1000002	Prdm1	NM_00754	"Mus musc	RefSeq	4.467769	6.524682	-4.16095	562
TC0700003	Thrsp	NM_00938	"Mus musc	RefSeq	5.861492	7.917593	-4.15861	563

TC1400001422.mm.1		uc007sdy.1	"alpha43-ta	UCSC Gene	5.268383	7.320926	-4.14837	564
TC080000C	Hmox1	NM_01044	"Mus musc	RefSeq	9.462677	7.410717	4.146689	565
TC1100002	Gm12185	NM_00104	"Mus musc	RefSeq	6.61688	8.668403	-4.14543	566
TC0200003	Ggta1	NM_00114	"Mus musc	RefSeq	6.102496	8.153398	-4.14365	567
TC170000C	Kank3	NM_03069	"Mus musc	RefSeq	7.137968	9.186007	-4.13544	568
TC1000002	Glt8d2	NM_02910	"Mus musc	RefSeq	6.399286	4.35223	4.132618	569
TC1400002	Gm10406	NM_00116	"Mus musc	RefSeq	5.588905	7.635497	-4.13129	570
TC1200001	Rin3	NM_17762	"Mus musc	RefSeq	6.053404	8.099956	-4.13117	571
TC1100003	Spns2	NM_00127	"Mus musc	RefSeq	6.661152	8.707454	-4.13046	572
TC1100002	Ccdc85a	NM_00116	"Mus musc	RefSeq	5.824889	7.870969	-4.12982	573
TC090000C	Pdgfd	NM_02792	"Mus musc	RefSeq	7.492661	9.53772	-4.1269	574
TC150000C	Fzd6	NM_00116	"Mus musc	RefSeq	6.815885	8.860856	-4.12665	575
TC050000C	Rasl11b	NM_02687	"Mus musc	RefSeq	7.749743	5.706059	4.12297	576
TC140000C	Sema3g	NM_00102	"Mus musc	RefSeq	6.007821	8.050647	-4.12052	577
TC0300001	Arsj	NM_17345	"Mus musc	RefSeq	5.770847	3.72814	4.120179	578
TC020000C	Pkp4	NM_02636	"Mus musc	RefSeq	7.25055	9.293219	-4.12007	579
TC0500003	Flt1	NM_01022	"Mus musc	RefSeq	8.849695	10.89171	-4.11821	580
TC0700002	Bcam	NM_02048	"Mus musc	RefSeq	7.728507	9.770509	-4.11817	581
TSUnmapp	Cd83	NM_00128	"CD83 anti	RefSeq	6.727712	4.686867	4.114865	582
TC0500003	Scarb1	NM_00120	"Mus musc	RefSeq	6.777017	8.817847	-4.11482	583
TC110000C	Pmp22	NM_00888	"Mus musc	RefSeq	10.64494	8.604355	4.11412	584
TC120000C	Galnt16	NM_00108	"Mus musc	RefSeq	6.816869	4.777218	4.111461	585
TC1400001	Gm8237	ENSMUST0	cdna:putat	ENSEMBL	5.579947	7.617453	-4.10535	586
TC0100003	Gm16340	XM_00308	"PREDICTEI	RefSeq	7.667882	9.699055	-4.08737	587
TC1200001	Clec14a	NM_02580	"Mus musc	RefSeq	5.673287	7.703499	-4.08465	588
TC090000C	Nxpe4	NM_17292	"Mus musc	RefSeq	6.185746	8.215783	-4.08415	589
TC130000C	Akr1c14	NM_13407	"Mus musc	RefSeq	5.18681	7.215397	-4.08005	590
TC1800001	Arhgap26	NM_17516	"Mus musc	RefSeq	5.504423	7.532906	-4.07976	591
TC1000002	Man1a	NM_00854	"Mus musc	RefSeq	9.210954	7.185753	4.070486	592
TC030000C	Sh3d19	NM_00108	"Mus musc	RefSeq	8.644668	6.620957	4.066284	593
TC190000C	Ppp1r14b	NM_00888	"Mus musc	RefSeq	8.507485	6.485613	4.061104	594
TC150000C	Sema5a	NM_00915	"Mus musc	RefSeq	6.702597	4.687331	4.042551	595
TC0100003	AI607873	NM_00120	"Mus musc	RefSeq	9.130141	7.117258	4.035879	596
TC0100001	Rgs16	NM_01126	"Mus musc	RefSeq	6.502544	8.514505	-4.0333	597
TC0600002	Podxl	NM_01372	"Mus musc	RefSeq	8.730446	10.74141	-4.03051	598
TC0300002	BC028528	NM_15351	"Mus musc	RefSeq	5.58246	7.592649	-4.02835	599
TC160000C	Cldn5	NM_01380	"Mus musc	RefSeq	7.600229	9.610172	-4.02766	600
TC0200002	Itih2	NM_01058	"Mus musc	RefSeq	5.878108	3.868363	4.02711	601
TC1400001	Gfra2	NM_00811	"Mus musc	RefSeq	6.668266	4.660679	4.021091	602
TC0800002	Ifi30	NM_02306	"Mus musc	RefSeq	8.533188	6.526268	4.019232	603
TC040000C	Acer2	NM_13930	"Mus musc	RefSeq	6.272257	8.274539	-4.00633	604
TC0500003	Hspb8	NM_03070	"Mus musc	RefSeq	7.809381	5.807644	4.004819	605
TC0400003	Tinagl1	NM_00116	"Mus musc	RefSeq	7.168042	9.167894	-3.99959	606
TC1100004	9930111J2	NM_00111	"Mus musc	RefSeq	6.419883	8.416406	-3.99037	607
TC0800001	Mmp15	NM_00860	"Mus musc	RefSeq	7.477737	9.473916	-3.98942	608
TC070000C	Slc1a5	NM_00920	"Mus musc	RefSeq	8.691047	6.696415	3.985144	609
TC0600001	Cecr2	NM_00112	"Mus musc	RefSeq	5.245179	7.239141	-3.98329	610

TC1400000	Gm8271	XM_98577	"PREDICTEI	RefSeq	4.072124	6.063965	-3.97744	611
TC1100003	Ntn1	NM_00874	"Mus musc	RefSeq	8.874011	6.889975	3.955982	612
TC0200000	Cercam	NM_20729	"Mus musc	RefSeq	7.161932	5.179253	3.952263	613
TC0300000	Nes	NM_01670	"Mus musc	RefSeq	6.379932	8.361745	-3.94989	614
TC0600002	Adamts9	NM_17531	"Mus musc	RefSeq	7.174805	9.155716	-3.94742	615
TC0600000	Loxl3	NM_01358	"Mus musc	RefSeq	6.919868	4.939767	3.945207	616
TC1500000	Dennd3	NM_00108	"Mus musc	RefSeq	6.240912	8.218893	-3.93941	617
TC1100001	Coro6	NM_13912	"Mus musc	RefSeq	6.723925	8.699787	-3.93363	618
TC1200002	Jag2	NM_01058	"Mus musc	RefSeq	7.590755	9.566197	-3.93249	619
TSUnmapp	Rora	NM_00128	"RAR-relate	RefSeq	8.017058	6.045063	3.923102	620
TC0300003	Lphn2	NM_00108	"Mus musc	RefSeq	6.810815	8.782295	-3.9217	621
TC1400002	Adcy4	NM_08043	"Mus musc	RefSeq	7.313784	9.285084	-3.92121	622
TC0200003	Stom	NM_01351	"Mus musc	RefSeq	7.032519	9.003495	-3.92033	623
TC1800000	Sncaip	NM_00119	"Mus musc	RefSeq	5.416858	7.387177	-3.91855	624
TC0400003	2610305D1	NM_14507	"Mus musc	RefSeq	6.194016	8.162921	-3.91471	625
TC0800000	D8Ertd82e	NM_17291	"Mus musc	RefSeq	6.363889	8.332679	-3.9144	626
TC1100001	Adap2	NM_17213	"Mus musc	RefSeq	7.703991	5.736362	3.911248	627
TC0100000	Xrcc5	NM_00953	"Mus musc	RefSeq	5.692978	3.726784	3.90736	628
TC1800000	Lama3	NM_01068	"Mus musc	RefSeq	5.053465	7.019546	-3.90705	629
TC0500000	Cytl1	NM_00108	"Mus musc	RefSeq	9.394667	7.428748	3.906615	630
TC0200005	Sdc4	NM_01152	"Mus musc	RefSeq	6.920483	4.954756	3.906095	631
TC0600001	Slc6a6	NM_00932	"Mus musc	RefSeq	8.24641	10.2075	-3.89355	632
TC0200001	Itgav	NM_00840	"Mus musc	RefSeq	8.248762	6.292297	3.881098	633
TC0X00002	Mtap7d3	NM_17729	"Mus musc	RefSeq	5.214694	3.258414	3.880601	634
TC0300001	Eltd1	NM_13322	"Mus musc	RefSeq	8.894864	10.85059	-3.87912	635
TC1300002	Rhobtb3	NM_02849	"Mus musc	RefSeq	6.618176	4.662451	3.879108	636
TC0100002	Pam	NM_01362	"Mus musc	RefSeq	9.73038	7.775074	3.877982	637
TC0100002	Steap3	NM_13318	"Mus musc	RefSeq	6.992749	5.037515	3.877788	638
TC0600000	Flnc	NM_00108	"Mus musc	RefSeq	7.507842	5.553737	3.874755	639
TC1200002	Ppp1r13b	NM_01162	"Mus musc	RefSeq	7.070798	9.024848	-3.87461	640
TC0900000	Casp1	NM_00980	"Mus musc	RefSeq	4.241741	6.195397	-3.87355	641
TC0400000	Nr4a3	NM_01574	"Mus musc	RefSeq	8.230714	6.277218	3.873119	642
TC1300001	Lrrc16a	NM_02682	"Mus musc	RefSeq	6.481943	4.530327	3.868076	643
TC0300002	Fnbp1l	NM_15311	"Mus musc	RefSeq	6.740664	8.692184	-3.86782	644
TC0900001	Myo5a	NM_01086	"Mus musc	RefSeq	6.410148	4.459637	3.865114	645
TC0200003	Pla2r1	NM_00886	"Mus musc	RefSeq	5.832213	3.882037	3.864217	646
TC0600001	Mgll	NM_01184	"Mus musc	RefSeq	7.9179	9.867882	-3.8637	647
TC0700001	Ucp2	NM_01167	"Mus musc	RefSeq	7.156424	9.105203	-3.86048	648
TC0500000	Cxcl2	NM_00914	"Mus musc	RefSeq	7.21829	5.269522	3.860447	649
TC1100003	Pecam1	NM_00103	"Mus musc	RefSeq	8.626008	10.57252	-3.85441	650
TC1000001	Ptprb	NM_02992	"Mus musc	RefSeq	9.312787	11.25839	-3.85199	651
TC1600001	Cd200	NM_01081	"Mus musc	RefSeq	7.342591	9.281214	-3.8334	652
TC0700004	Sult1a1	NM_13367	"Mus musc	RefSeq	7.45224	9.390014	-3.83114	653
TC0100001	Fam129a	NM_02201	"Mus musc	RefSeq	8.147407	6.209837	3.830599	654
TC0900000	Vsig2	NM_02051	"Mus musc	RefSeq	6.306069	8.242873	-3.82857	655
TC0600001	Ica1	NM_00125	"Mus musc	RefSeq	5.062598	6.998106	-3.82513	656
TC1100000	Pik3r6	NM_00100	"Mus musc	RefSeq	5.973959	7.909165	-3.82433	657

TC0900002	Fam46a	NM_00116	"Mus musc	RefSeq	6.882707	4.948593	3.821434	658
TC1100003	Dgke	NM_01950	"Mus musc	RefSeq	5.151161	7.084694	-3.8199	659
TC190000C	Plce1	NM_01958	"Mus musc	RefSeq	6.928008	4.995342	3.8176	660
TC1500001	Mtss1	NM_00114	"Mus musc	RefSeq	6.302588	8.23144	-3.80752	661
TC1500001	Mfng	NM_00859	"Mus musc	RefSeq	5.941147	7.869814	-3.80703	662
TC150000C	Myo10	NM_01947	"Mus musc	RefSeq	8.12806	10.05431	-3.80066	663
TC010000C	Il1rl2	NM_13319	"Mus musc	RefSeq	6.601445	4.679626	3.789005	664
TC0900002	Tspan3	NM_01979	"Mus musc	RefSeq	8.675427	6.756628	3.781082	665
TC1100002	9930111J2	NM_17343	"Mus musc	RefSeq	6.920728	8.836007	-3.77187	666
TC1100001	Fkbp10	NM_00116	"Mus musc	RefSeq	8.492075	6.576841	3.77175	667
TC1200001	Tnfaip2	NM_00939	"Mus musc	RefSeq	7.455571	5.54049	3.77135	668
TC1700002	Emilin2	NM_14515	"Mus musc	RefSeq	6.603911	4.690439	3.767146	669
TC1900001	Aldh18a1	NM_15355	"Mus musc	RefSeq	7.255342	5.343256	3.763529	670
TC010000C	Sdpr	NM_13874	"Mus musc	RefSeq	8.888308	10.79406	-3.74703	671
TC010000C	Nrp2	NM_00107	"Mus musc	RefSeq	7.807642	9.712002	-3.74343	672
TC070000C	Nova2	NM_00102	"Mus musc	RefSeq	6.239198	8.140535	-3.73559	673
TC0500003	Gbp9	NM_17277	"Mus musc	RefSeq	8.14701	10.04827	-3.7354	674
TC0200004	Foxs1	NM_01022	"Mus musc	RefSeq	7.505862	5.606825	3.729642	675
TC0800002	Ces1d	NM_05320	"Mus musc	RefSeq	5.846298	3.947744	3.728393	676
TC0900001	Mst1r	NM_00907	"Mus musc	RefSeq	6.552412	4.655879	3.723174	677
TC1100003	Abi3	NM_02565	"Mus musc	RefSeq	5.985415	7.880374	-3.71911	678
TC0200002	Plcb4	NM_01382	"Mus musc	RefSeq	7.64082	9.535435	-3.71823	679
TC0200001	Wt1	NM_14478	"Mus musc	RefSeq	5.910618	7.804765	-3.71702	680
TC0400001	Rex2	NM_00117	"Mus musc	RefSeq	5.862242	7.754966	-3.71336	681
TC0200004	Duoxa1	NM_14539	"Mus musc	RefSeq	6.842638	4.950766	3.711165	682
TC1100003	Icam2	NM_01049	"Mus musc	RefSeq	5.811765	7.70347	-3.71074	683
TC0700002	LOC100862	XM_00368	"PREDICTEI	MGI/Jackso	6.880693	8.771577	-3.70862	684
TC0X00003	Maged2	NM_00119	"Mus musc	RefSeq	7.978608	6.08856	3.706476	685
TC1400001	Gm8050	XM_00308	"PREDICTEI	RefSeq	4.754637	6.643633	-3.70377	686
TC0600003	Bhlhe41	NM_02446	"Mus musc	RefSeq	6.231255	4.343578	3.700389	687
TC0600003	Gimap5	NM_17503	"Mus musc	RefSeq	4.722452	6.60926	-3.69816	688
TC0900001	Gk5	NM_17735	"Mus musc	RefSeq	6.629787	4.743733	3.696229	689
TC1100004	Cygb	NM_03020	"Mus musc	RefSeq	9.623008	7.73756	3.694676	690
TC0900002	Nnmt	NM_01092	"Mus musc	RefSeq	6.194697	4.309311	3.694518	691
TC1400001	Stab1	NM_13867	"Mus musc	RefSeq	6.20508	8.089871	-3.69299	692
TC010000C	Gm9994	NM_00120	"Mus musc	RefSeq	7.126907	5.242493	3.692029	693
TC0X0000C	Bgn	NM_00754	"Mus musc	RefSeq	11.45118	9.567532	3.690075	694
TC010000C	Inpp5d	NM_01056	"Mus musc	RefSeq	5.13881	7.021172	-3.68678	695
TC1700002	C4b	NM_00978	"Mus musc	RefSeq	6.71874	4.836391	3.686748	696
TC0400004	Mmp23	NM_01198	"Mus musc	RefSeq	7.224533	5.343928	3.682294	697
TC190000C	Entpd1	NM_00984	"Mus musc	RefSeq	6.200724	8.078316	-3.67461	698
TC110000C	Gas7	NM_00808	"Mus musc	RefSeq	6.492656	4.61711	3.669405	699
TC140000C	Adk	NM_13407	"Mus musc	RefSeq	6.929621	5.055427	3.665968	700
TC120000C	Meox2	NM_00858	"Mus musc	RefSeq	6.808902	8.682841	-3.66532	701
TC080000C	Thsd1	NM_01957	"Mus musc	RefSeq	5.584012	7.457334	-3.66375	702
TC1400002	Spry2	NM_01189	"Mus musc	RefSeq	8.3523	6.479748	3.661797	703
TC0500002	Arap2	NM_17840	"Mus musc	RefSeq	4.954812	6.827186	-3.66135	704

TC100000C	Arhgap18	NM_17683	"Mus musc	RefSeq	7.589555	9.458432	-3.65248	705
TC1400001	Gm3755	ENSMUST0	cdna:putat	ENSEMBL	4.072683	5.940112	-3.64882	706
TC030000C	Fabp5	NM_01063	"Mus musc	RefSeq	5.722105	7.588801	-3.64696	707
TC1300002	Itga1	NM_00103	"Mus musc	RefSeq	8.019747	9.885859	-3.64549	708
TC080000C	Slc7a2	NM_00751	"Mus musc	RefSeq	6.346763	4.48229	3.641349	709
TC0700002	Ceacam2	NM_00111	"Mus musc	RefSeq	7.08426	8.948382	-3.64046	710
TC1000002	Cdh23	NM_02337	"Mus musc	RefSeq	6.051384	7.91452	-3.63798	711
TC1000002	Gstt1	NM_00818	"Mus musc	RefSeq	8.433885	6.571204	3.636829	712
TC0X0000C	Tspan7	NM_01963	"Mus musc	RefSeq	9.550197	11.41012	-3.62987	713
TC050000C	Kit	NM_00112	"Mus musc	RefSeq	5.028972	6.887492	-3.62636	714
TC1600001	Alcam	NM_00965	"Mus musc	RefSeq	5.991271	4.135899	3.61845	715
TC1800001	Sema6a	NM_01874	"Mus musc	RefSeq	7.936245	9.791451	-3.61803	716
TC040000C	Unc13b	NM_00108	"Mus musc	RefSeq	5.768184	7.623287	-3.61778	717
TC1000001	Epyc	NM_00788	"Mus musc	RefSeq	5.014399	3.163133	3.608167	718
TC1700002	Ptprs	NM_00125	"Mus musc	RefSeq	8.365866	6.516004	3.604657	719
TC130000C	Arrdc3	NM_00104	"Mus musc	RefSeq	8.50894	6.659193	3.60437	720
TC0200002	Itga8	NM_00100	"Mus musc	RefSeq	6.222319	4.374153	3.600422	721
TC040000C	LOC100861	XM_00368	"PREDICTEI	RefSeq	7.066457	5.218317	3.600357	722
TC040000C	Gm2564	XM_00147	"PREDICTEI	RefSeq	7.164872	5.316733	3.600355	723
TC4_JH584	CR974586.:	ENSMUST0	cdna:novel	ENSEMBL	6.898593	5.050454	3.600355	723
TC0300002	Efna1	NM_01010	"Mus musc	RefSeq	7.234004	9.081153	-3.59789	725
TC160000C	Ahsg	NM_01346	"Mus musc	RefSeq	6.706879	4.860234	3.596628	726
TC0600001	Emp1	NM_01012	"Mus musc	RefSeq	10.27013	8.424067	3.595185	727
TC0100003	Pydc4	NM_00117	"Mus musc	RefSeq	3.800287	5.644632	-3.5909	728
TC1100001	Ace	NM_20762	"Mus musc	RefSeq	10.41822	8.574012	3.590558	729
TC0X00003	Kctd12b	NM_17542	"Mus musc	RefSeq	6.267896	8.106967	-3.5778	730
TC0200001	Slc43a1	NM_00108	"Mus musc	RefSeq	7.175943	5.337261	3.576831	731
TC1300002	Snx18	NM_13079	"Mus musc	RefSeq	8.425802	6.587266	3.576469	732
TC0600002	Plxnd1	NM_02637	"Mus musc	RefSeq	8.804252	10.64187	-3.57419	733
TC030000C	Gja5	NM_00127	"Mus musc	RefSeq	5.266629	7.103857	-3.57323	734
TC050000C	Slc4a4	NM_00113	"Mus musc	RefSeq	6.008198	4.172754	3.568812	735
TC0300001	Sec24d	NM_02713	"Mus musc	RefSeq	8.832472	6.997408	3.567872	736
TC0800001	Rasa3	NM_00902	"Mus musc	RefSeq	8.209988	6.375978	3.565267	737
TC0600002	Dennd2a	NM_17247	"Mus musc	RefSeq	6.78082	4.948172	3.561902	738
TC1400002	Rgcc	NM_02542	"Mus musc	RefSeq	8.524854	10.3539	-3.55301	739
TC0200005	Egfl7	NM_17844	"Mus musc	RefSeq	7.318739	9.147287	-3.55179	740
TC1600001	Gp1bb	NM_01032	"Mus musc	RefSeq	7.426983	5.598973	3.55047	741
TC0600003	Mansc1	NM_02634	"Mus musc	RefSeq	6.087177	7.91414	-3.54789	742
TC0600002	Lrig1	NM_00837	"Mus musc	RefSeq	7.289636	5.465094	3.541945	743
TC1400001	Gm2244	XM_00147	"PREDICTEI	RefSeq	4.390039	6.213528	-3.53936	744
TC140000C	Gm3278	XM_00147	"PREDICTEI	RefSeq	4.34218	6.165668	-3.53936	745
TC140000C	Tnfrsf10b	NM_02027	"Mus musc	RefSeq	5.547969	7.369462	-3.53447	746
TC0500003	Eln	NM_00792	"Mus musc	RefSeq	9.097499	7.276485	3.533294	747
TC0400002	Car8	NM_00759	"Mus musc	RefSeq	6.977909	8.798872	-3.53317	748
TC1400002	Dgkh	NM_00125	"diacylglyc	RefSeq	5.897746	7.717724	-3.53076	749
TC0100002	Hecw2	NM_00100	"Mus musc	RefSeq	6.432998	8.251975	-3.52831	750
TC150000C	Dap	NM_14605	"Mus musc	RefSeq	9.641358	7.826568	3.518084	751

TC1500000	Gpihbp1	NM_02673	"Mus musc	RefSeq	9.315295	11.12809	-3.51323	752
TC0100003	Pcp4l1	NM_02555	"Mus musc	RefSeq	6.506325	8.316781	-3.50753	753
TC1900000	1810055G(	NM_02807	"Mus musc	RefSeq	7.686607	5.876907	3.505694	754
TC0300002	Gpsm2	NM_02952	"Mus musc	RefSeq	5.628934	3.82091	3.501624	755
TC0400001	Heyl	NM_01390	"Mus musc	RefSeq	8.024878	6.218208	3.498339	756
TC0600003	Leprel2	NM_01353	"Mus musc	RefSeq	7.673265	5.866665	3.498169	757
TC0400001	Zfp600	NM_00117	"Mus musc	RefSeq	5.265429	7.071439	-3.49674	758
TC1500002	Zfp385a	NM_01386	"Mus musc	RefSeq	8.052419	6.248808	3.490929	759
TC0700004	GM8989	ENSMUST0	cdna:know	ENSEMBL	5.456032	7.259488	-3.49055	760
TC1800000	4833403I1!	NM_02900	"Mus musc	RefSeq	5.233803	3.431529	3.487695	761
TC1800000	Iigp1	NM_02179	"Mus musc	RefSeq	5.876206	7.678157	-3.48692	762
TC0200002	Hspa12b	NM_02830	"Mus musc	RefSeq	7.566218	9.367924	-3.48632	763
TC1800000	Apcdd1	NM_13323	"Mus musc	RefSeq	7.475742	5.676829	3.47958	764
TC0X00001	Col4a5	NM_00773	"Mus musc	RefSeq	6.262047	4.464309	3.476747	765
TC0500002	Lnx1	NM_00115	"Mus musc	RefSeq	5.60033	7.397901	-3.47634	766
TC0600003	Zfp9	NM_01176	"Mus musc	RefSeq	7.060692	5.264874	3.472123	767
TC1800000	Rnf125	NM_02630	"Mus musc	RefSeq	6.92385	8.718054	-3.46824	768
TC1200002	Serpina3c	NM_00845	"Mus musc	RefSeq	5.059545	3.267366	3.463376	769
TC0700004	Galnt18	NM_17373	"Mus musc	RefSeq	6.189117	7.978189	-3.45593	770
TC0400001	Gm13145	ENSMUST0	cdna:novel	ENSEMBL	5.841904	7.627799	-3.44832	771
TC0X00003	Reps2	NM_17825	"Mus musc	RefSeq	6.165912	4.384307	3.438084	772
TC0600001	Fgd5	NM_17273	"Mus musc	RefSeq	7.009678	8.789437	-3.43369	773
TC1400002	Gata4	NM_00809	"Mus musc	RefSeq	7.395132	5.615617	3.433107	774
TC1400002	Gm8206	ENSMUST0	cdna:putat	ENSEMBL	5.106857	6.88637	-3.4331	775
TC1200000	Dock4	NM_17280	"Mus musc	RefSeq	6.437785	8.21583	-3.42961	776
TC0500000	Art3	NM_18172	"Mus musc	RefSeq	5.021818	6.797947	-3.42506	777
TC4_JH584	Gm2023	ENSMUST0	cdna:know	ENSEMBL	7.408427	5.632944	3.423526	778
TC0500003	Gbp4	NM_00862	"Mus musc	RefSeq	7.726426	9.50166	-3.42294	779
TC1400000	Spata13	NM_00103	"Mus musc	RefSeq	5.798556	7.572664	-3.42027	780
TC1000000	Egr2	NM_01011	"Mus musc	RefSeq	8.790263	7.01695	3.418381	781
TC0X00001	Figf	NM_01021	"Mus musc	RefSeq	5.981613	4.209817	3.414788	782
TC1500000	Khdrbs3	NM_01015	"Mus musc	RefSeq	6.448648	4.677285	3.413763	783
TC0X00000	Zc3h12b	NM_00103	"Mus musc	RefSeq	5.255827	3.48835	3.40458	784
TC1100004	Fasn	NM_00798	"Mus musc	RefSeq	7.482739	5.71532	3.404444	785
TC0500001	Fry	NM_17288	"Mus musc	RefSeq	6.756337	8.523006	-3.40267	786
TC0500002	Kdr	NM_01061	"Mus musc	RefSeq	9.790692	11.55608	-3.39965	787
TC1700002	Tgif1	NM_00116	"Mus musc	RefSeq	7.546278	5.781314	3.398655	788
TC1100000	Scgb3a1	NM_05403	"Mus musc	RefSeq	4.912118	6.676939	-3.39832	789
TC1200000	Akap5	NM_00110	"Mus musc	RefSeq	4.572908	6.337678	-3.3982	790
TC1900000	Map4k2	NM_00900	"Mus musc	RefSeq	6.710745	8.475012	-3.39701	791
TC1700002	Nudt12	NM_02649	"Mus musc	RefSeq	5.179189	6.942562	-3.39491	792
TC1500000	Shank3	NM_02142	"Mus musc	RefSeq	6.770604	8.533467	-3.39371	793
TC0500001	Tbx3	NM_19805	"Mus musc	RefSeq	6.110752	7.871874	-3.38962	794
TC1000001	1500009L1	NM_00114	"Mus musc	RefSeq	6.28104	4.520232	3.388879	795
TC0100001	Rgs5	NM_00906	"Mus musc	RefSeq	7.870567	9.631337	-3.38879	796
TC1900001	Pgm5	NM_17501	"Mus musc	RefSeq	5.128103	6.888394	-3.38766	797
TC0200000	Olfm1	NM_00103	"Mus musc	RefSeq	7.274078	5.514916	3.385014	798

TC1100004	Psme2	ENSMUST0	cdna:know	ENSEMBL	8.26799	10.02632	-3.38306	799
TC1700000	Gpr116	NM_00108	"Mus musc	RefSeq	10.01626	11.77437	-3.38255	800
TC0200000	Nrarp	NM_02598	"Mus musc	RefSeq	6.241782	7.998193	-3.37857	801
TC0800001	1810011O1	NM_02693	"Mus musc	RefSeq	7.623299	9.378691	-3.37618	802
TC1200002	Dio2	NM_01005	"Mus musc	RefSeq	5.799118	4.044427	3.37454	803
TC1400000	Oxnad1	NM_14546	"Mus musc	RefSeq	7.286997	5.532911	3.373126	804
TC1500001	Lynx1	NM_01183	"Mus musc	RefSeq	7.448249	5.695198	3.370706	805
TC1100003	Tmem88	NM_02591	"Mus musc	RefSeq	8.680634	10.4321	-3.36701	806
TC0700000	Vimp	NM_02443	"Mus musc	RefSeq	7.335127	5.585721	3.362201	807
TC1100001	Scarf1	NM_00100	"Mus musc	RefSeq	6.497578	8.245196	-3.35804	808
TC1400000	Gdf10	NM_14574	"Mus musc	RefSeq	6.683163	4.937479	3.353538	809
TC0200005	Pmepa1	NM_02299	"Mus musc	RefSeq	8.633917	6.890003	3.349426	810
TC0100001	Pik3c2b	NM_00109	"Mus musc	RefSeq	6.901703	8.645229	-3.34853	811
TC0100002	Ptp4a1	NM_01120	"Mus musc	RefSeq	9.985772	8.243016	3.346739	812
TC0900002	Nxpe2	NM_03006	"Mus musc	RefSeq	3.670185	5.412404	-3.34549	813
TC1000000	Eid3	NM_02549	"Mus musc	RefSeq	7.497946	5.756002	3.344856	814
TC0600002	Pdzrn3	NM_01888	"Mus musc	RefSeq	7.033822	5.292046	3.344466	815
TC0800000	Mcf2l	NM_17807	"Mus musc	RefSeq	6.700693	8.442344	-3.34418	816
TC0300002	Adamtsl4	NM_14489	"Mus musc	RefSeq	8.300576	6.559298	3.343312	817
TC1600002	Vasn	NM_13930	"Mus musc	RefSeq	7.396008	5.655572	3.341361	818
TC0500002	Rasgef1b	NM_14583	"Mus musc	RefSeq	6.672764	4.935249	3.334603	819
TC1700001	H2-T24	NM_00820	"Mus musc	RefSeq	6.138621	7.872969	-3.32729	820
TC1900001	Sorbs1	NM_00103	"Mus musc	RefSeq	6.563206	8.297037	-3.3261	821
TC0200005	Lama5	NM_00108	"Mus musc	RefSeq	6.225152	7.958604	-3.32523	822
TC0X00000	Usp11	NM_14562	"Mus musc	RefSeq	6.367905	4.635191	3.323525	823
TC1100000	Sh3pxd2b	NM_17736	"Mus musc	RefSeq	6.970556	5.237903	3.323384	824
TC1100001	Gngt2	NM_02312	"Mus musc	RefSeq	5.508459	7.240718	-3.32248	825
TC1000002	Nts	NM_02443	"Mus musc	RefSeq	4.31959	6.051842	-3.32246	826
TC1100000	Stk10	NM_00928	"Mus musc	RefSeq	7.306523	9.037001	-3.31838	827
TC0900000	Eepd1	NM_02618	"Mus musc	RefSeq	6.586163	8.313174	-3.31041	828
TC0300002	Il6ra	NM_01055	"Mus musc	RefSeq	7.436773	5.712475	3.304193	829
TC1000002	Susd2	NM_02789	"Mus musc	RefSeq	6.788504	5.064728	3.302998	830
TC0100002	Myo1b	NM_00116	"Mus musc	RefSeq	6.74653	8.470303	-3.30299	831
TC1700001	Tnfrsf12a	NM_01374	"Mus musc	RefSeq	8.573317	6.850997	3.299666	832
TC1200001	Cys1	NM_13868	"Mus musc	RefSeq	6.251379	4.529691	3.298221	833
TC0400000	Gm10590	ENSMUST0	cdna:know	ENSEMBL	5.2212	3.501727	3.293161	834
TC0900001	Fli1	NM_00802	"Mus musc	RefSeq	8.447342	10.1645	-3.28788	835
TC0700004	Sez6l2	NM_00125	"Mus musc	RefSeq	5.73711	7.452633	-3.28416	836
TC1200001	Prkd1	NM_00885	"Mus musc	RefSeq	5.822965	7.537014	-3.2808	837
TC0100001	Itpkb	NM_00108	"Mus musc	RefSeq	8.827881	10.53373	-3.2622	838
TC1600001	Cbr3	NM_17304	"Mus musc	RefSeq	5.786885	4.081583	3.260972	839
TC0100003	Hsd11b1	NM_00104	"Mus musc	RefSeq	6.017675	4.312706	3.260219	840
TC0500001	Chek2	NM_01668	"Mus musc	RefSeq	5.195576	6.90049	-3.2601	841
TC1800001	Pcdh1	NM_02935	"Mus musc	RefSeq	6.848008	8.551322	-3.25648	842
TC1200000	Ttc8	NM_02955	"Mus musc	RefSeq	6.148512	4.445494	3.255813	843
TC1400000	Thrb	NM_00111	"Mus musc	RefSeq	5.960391	4.2582	3.253948	844
TC0700002	Peg3	NM_00881	"Mus musc	RefSeq	7.448122	5.747261	3.250949	845

TC1100000	Ubtd2	NM_17378	"Mus musc	RefSeq	8.153088	6.455248	3.244149	846
TC1800001	LOC10086	XM_00368	"PREDICTE	RefSeq	8.589851	6.89852	3.229545	847
TC0800002	B3gnt9	NM_17887	"Mus musc	RefSeq	6.480306	4.789002	3.229485	848
TC1100003	Arhgap27	NM_13371	"Mus musc	RefSeq	5.945339	7.635298	-3.22648	849
TC1800001	Epb4.1l4a	NM_01351	"Mus musc	RefSeq	5.390823	7.080604	-3.22608	850
TC1000002	Dram1	NM_02787	"Mus musc	RefSeq	7.506444	5.818141	3.222774	851
TC0400001	Pik3r3	NM_18158	"Mus musc	RefSeq	6.468929	8.154576	-3.21685	852
TC0100002	Gli2	NM_00108	"Mus musc	RefSeq	6.863363	5.180046	3.211655	853
TC1100001	Rab34	NM_00115	"Mus musc	RefSeq	6.70211	5.019956	3.209067	854
TC0900003	Parp3	NM_14561	"Mus musc	RefSeq	7.243278	5.561714	3.207755	855
TC1400002	Dock9	NM_00108	"Mus musc	RefSeq	8.719766	10.40125	-3.20758	856
TC0900000	Arhgap32	NM_00119	"Mus musc	RefSeq	7.107834	5.426532	3.207173	857
TC0500000	Sod3	NM_01143	"Mus musc	RefSeq	6.534042	4.852806	3.207026	858
TC0300001	Syde2	NM_00116	"Mus musc	RefSeq	6.148081	4.467345	3.205915	859
TC1300000	Slc12a7	NM_01139	"Mus musc	RefSeq	5.994318	7.669766	-3.19419	860
TC1700000	Notch4	NM_01092	"Mus musc	RefSeq	7.099857	8.77341	-3.18999	861
TC0900000	Ets1	NM_00103	"Mus musc	RefSeq	7.92161	9.593784	-3.18695	862
TC1600000	Ppm1f	NM_17683	"Mus musc	RefSeq	6.950652	8.622783	-3.18685	863
TC0300000	Tiparp	NM_17889	"Mus musc	RefSeq	7.789461	6.117999	3.185372	864
TC0300000	P2ry1	NM_00877	"Mus musc	RefSeq	5.700807	4.029998	3.183931	865
TC0200002	Camk1d	NM_17734	"Mus musc	RefSeq	5.785823	4.116735	3.180135	866
TC0300001	Hey1	NM_01042	"Mus musc	RefSeq	8.362531	10.03145	-3.17976	867
TC1400000	Sox7	NM_01144	"Mus musc	RefSeq	7.354761	9.023379	-3.1791	868
TC0800001	Cdh5	NM_00986	"Mus musc	RefSeq	10.32769	11.99578	-3.17794	869
TC1700001	Slc9a3r2	NM_02344	"Mus musc	RefSeq	9.220765	10.88848	-3.17711	870
TC0300000	She	NM_17253	"Mus musc	RefSeq	6.924658	8.592298	-3.17695	871
TC0500003	Oas1a	NM_14521	"Mus musc	RefSeq	5.74067	7.402138	-3.16338	872
TC1900000	Vldlr	NM_00116	"Mus musc	RefSeq	6.403839	4.744495	3.158729	873
TC1500000	Apobec3	NM_00116	"Mus musc	RefSeq	5.680038	7.338811	-3.15748	874
TC1900000	Efemp2	NM_02147	"Mus musc	RefSeq	8.044758	6.387903	3.153284	875
TC1000002	Elk3	NM_00128	"ELK3, mer	RefSeq	8.314332	9.971111	-3.15312	876
TC0200001	Mertk	NM_00858	"Mus musc	RefSeq	6.377225	8.03397	-3.15304	877
TC0900002	Col12a1	NM_00773	"Mus musc	RefSeq	5.759923	4.103233	3.152923	878
TC1100003	Slc16a13	NM_17237	"Mus musc	RefSeq	6.020665	7.676581	-3.15123	879
TC0700002	Plekhf1	NM_02441	"Mus musc	RefSeq	7.985672	6.329814	3.151105	880
TC0900000	Kdelc2	NM_21244	"Mus musc	RefSeq	7.694461	6.039806	3.148479	881
TC0200000	Bend7	NM_00119	"Mus musc	RefSeq	5.103446	6.757948	-3.14815	882
TC1000002	Trhde	NM_14624	"Mus musc	RefSeq	4.95592	3.3015	3.147966	883
TC1700001	Epas1	NM_01013	"Mus musc	RefSeq	9.723208	11.37728	-3.14722	884
TC1300001	Gfod1	NM_00103	"Mus musc	RefSeq	5.094979	6.748024	-3.14497	885
TC0500002	Apbb2	NM_00120	"Mus musc	RefSeq	7.609606	9.262085	-3.14373	886
TC1800000	Gata6	NM_01025	"Mus musc	RefSeq	8.799044	7.147123	3.142518	887
TC0500000	Ppbp	NM_02378	"Mus musc	RefSeq	5.213469	6.864705	-3.14103	888
TC0100002	Dsel	NM_00108	"Mus musc	RefSeq	7.97892	6.328453	3.139352	889
TC0200000	Ptgs1	NM_00896	"Mus musc	RefSeq	5.223124	6.872528	-3.13704	890
TC0200004	Kcnip3	NM_00111	"Mus musc	RefSeq	7.118133	5.470966	3.13218	891
TC0600000	Arhgef5	NM_13367	"Mus musc	RefSeq	5.551171	7.197644	-3.13067	892

TC1400000	Ptprg	NM_00898	"Mus musc	RefSeq	8.013914	9.659748	-3.12929	893
TC0300001	Bdh2	ENSMUST0	cdna:know	ENSEMBL	5.755503	4.11183	3.124603	894
TC0400004	Mup12	NM_00119	"Mus musc	RefSeq	5.498737	3.856426	3.121655	895
TC0600001	Vwf	NM_01170	"Mus musc	RefSeq	5.300607	6.942121	-3.11993	896
TC1900001	Mamdc2	NM_17485	"Mus musc	RefSeq	6.532204	4.890732	3.11984	897
TC0X00001	Rgag1	NM_00104	"Mus musc	RefSeq	4.923137	3.283962	3.114877	898
TC1600001	Arhgap31	NM_02026	"Mus musc	RefSeq	8.081422	9.71881	-3.11102	899
TC0700004	Tnfrsf22	NM_02368	"Mus musc	RefSeq	6.210883	7.847877	-3.11017	900
TC0X00001	Klhl13	NM_02616	"Mus musc	RefSeq	6.028212	4.392531	3.107342	901
TC0700000	Prkd2	NM_00125	"Mus musc	RefSeq	8.041866	9.677518	-3.10728	902
TC1200001	Inf2	NM_19841	"Mus musc	RefSeq	6.186447	7.821819	-3.10668	903
TC1600000	Fetub	NM_00108	"Mus musc	RefSeq	5.607222	3.972197	3.105929	904
TC1400001	Gm3248	ENSMUST0	cdna:putat	ENSEMBL	5.350426	6.984744	-3.10441	905
TC0500002	Evc	NM_02129	"Mus musc	RefSeq	6.708596	5.075077	3.102689	906
TC0900003	Sema3f	NM_01134	"Mus musc	RefSeq	6.513952	8.147071	-3.10183	907
TC1500001	Nr4a1	NM_01044	"Mus musc	RefSeq	11.27286	9.640124	3.100995	908
TC0600001	Cxcl12	NM_02170	"Mus musc	RefSeq	7.895506	9.527551	-3.09952	909
TC1200002	Ston2	NM_17536	"Mus musc	RefSeq	6.443878	4.814449	3.093905	910
TC0X00001	Magix	NM_01883	"Mus musc	RefSeq	5.318495	6.946167	-3.09014	911
TC0600002	Skap2	NM_01877	"Mus musc	RefSeq	6.26362	7.887449	-3.08192	912
TC1600000	B4galt4	NM_01980	"Mus musc	RefSeq	6.304342	7.926302	-3.07793	913
TC1900000	Trpm3	NM_00103	"Mus musc	RefSeq	4.81215	6.432326	-3.07413	914
TC0300002	Syt11	NM_01880	"Mus musc	RefSeq	6.501293	4.881396	3.073531	915
TC1700000	Rps6ka2	NM_01129	"Mus musc	RefSeq	6.523573	8.143048	-3.07263	916
TC0X00002	Apln	NM_01391	"Mus musc	RefSeq	3.780895	5.399175	-3.07009	917
TC1700002	Ptprm	NM_00898	"Mus musc	RefSeq	8.107856	9.725812	-3.0694	918
TC0500000	Yes1	NM_00120	"Mus musc	RefSeq	6.478997	8.096612	-3.06867	919
TC1100004	Caskin2	NM_08064	"Mus musc	RefSeq	6.938124	8.555474	-3.06811	920
TC0500002	Sh3tc1	NM_19434	"Mus musc	RefSeq	5.914776	7.531604	-3.067	921
TC1300002	Naip6	NM_01087	"Mus musc	RefSeq	4.080441	5.695704	-3.06367	922
TC1500002	Rapgef3	NM_14485	"Mus musc	RefSeq	6.769937	8.384433	-3.06205	923
TC1100001	Slfn2	NM_01140	"Mus musc	RefSeq	4.964502	6.578086	-3.06011	924
TC0400003	Epb4.1	NM_00112	"Mus musc	RefSeq	6.231545	7.84481	-3.05943	925
TC1000001	Ikbip	NM_02616	"Mus musc	RefSeq	7.041546	5.434656	3.045945	926
TC0600001	Plekha5	NM_14492	"Mus musc	RefSeq	7.857065	6.252832	3.040341	927
TC0800000	Pdlim3	NM_01679	"Mus musc	RefSeq	8.281922	6.677869	3.039961	928
TC0900000	Gucy1a2	NM_00103	"Mus musc	RefSeq	5.682624	4.078641	3.039814	929
TC1400001	LOC10050	XM_00308	"PREDICTE	MGI/Jackso	3.426813	5.029967	-3.03807	930
TC0100003	Ifi203	NM_00832	"Mus musc	RefSeq	7.655429	9.258284	-3.03744	931
TC0400000	Chd7	NM_00127	"Mus musc	RefSeq	5.489805	7.092553	-3.03721	932
TC0600001	Gm17482	ENSMUST0	cdna:know	ENSEMBL	1.584963	3.187302	-3.03635	933
TC0800002	Tppp3	NM_02648	"Mus musc	RefSeq	8.772597	7.170305	3.036253	934
TC0600001	Lmcd1	NM_14479	"Mus musc	RefSeq	6.968441	8.570719	-3.03622	935
TC0600003	Dennd5b	NM_17719	"Mus musc	RefSeq	5.786188	7.385641	-3.03028	936
TC0600002	Ssu2	NM_17552	"Mus musc	RefSeq	5.711313	7.30952	-3.02767	937
TC1400002	Abcc4	NM_00116	"Mus musc	RefSeq	6.581976	8.180095	-3.02748	938
TC0400001	Mfap2	NM_00116	"Mus musc	RefSeq	7.801673	6.20366	3.027261	939

TC0200005	Kcnb1	NM_00842	"Mus musc	RefSeq	5.812785	7.408759	-3.02299	940
TC1500001	Apol10b	NM_17782	"Mus musc	RefSeq	5.251379	6.843884	-3.01573	941
TC0200003	Notch1	NM_00871	"Mus musc	RefSeq	7.68304	9.274554	-3.01365	942
TC0400001	Epha2	NM_01013	"Mus musc	RefSeq	6.421393	8.01174	-3.01122	943
TC1000001	Fgd6	NM_05307	"Mus musc	RefSeq	4.51462	6.104834	-3.01094	944
TC170000C	Pde9a	NM_00116	"Mus musc	RefSeq	7.659816	6.069822	3.010481	945
TC180000C	Adamts19	NM_17550	"Mus musc	RefSeq	5.433691	3.846115	3.00544	946
TC1400001	Lpar6	NM_17511	"Mus musc	RefSeq	2.957351	4.543946	-3.0034	947
TC0400003	Alpl	NM_00128	"alkaline pl	RefSeq	7.038041	8.623761	-3.00158	948
TC060000C	Aqp1	NM_00747	"Mus musc	RefSeq	10.15931	11.74452	-3.00052	949
TC1700002	H2-Q7	NM_01039	"Mus musc	RefSeq	7.043316	8.628106	-2.99964	950
TC1400002	Lrch1	NM_00125	"Mus musc	RefSeq	6.283351	7.867213	-2.99771	951
TC1700002	Map4k3	NM_00108	"Mus musc	RefSeq	7.851901	9.434829	-2.99577	952
TC0500001	Cd36	NM_00115	"Mus musc	RefSeq	9.984305	11.56691	-2.99509	953
TC1100002	Gm5431	NM_00102	"Mus musc	RefSeq	4.589523	6.16809	-2.98673	954
TC0100003	Lamc1	NM_01068	"Mus musc	RefSeq	11.06996	9.491559	2.986395	955
TC0100002	Mgat4a	NM_17387	"Mus musc	RefSeq	6.134057	7.711124	-2.98363	956
TC0400002	Ptpn3	NM_01120	"Mus musc	RefSeq	5.796106	4.219515	2.982642	957
TC1000002	Cep85l	NM_00120	"Mus musc	RefSeq	7.077921	8.654286	-2.98218	958
TC0100003	Tgfb2	NM_00936	"Mus musc	RefSeq	8.009201	6.433561	2.980677	959
TC0100003	Rasal2	NM_17764	"Mus musc	RefSeq	6.910498	8.485112	-2.97856	960
TC0900001	Slc9a9	NM_17790	"Mus musc	RefSeq	5.738078	4.164317	2.976797	961
TC190000C	Stambpl1	NM_02968	"Mus musc	RefSeq	6.308629	4.736523	2.973384	962
TC0200003	Lrrc55	NM_00103	"Mus musc	RefSeq	5.640321	4.069627	2.970476	963
TC0600002	Rassf4	NM_17804	"Mus musc	RefSeq	5.042342	6.611538	-2.96739	964
TC0400002	Penk	NM_00100	"Mus musc	RefSeq	6.653667	5.084844	2.966626	965
TC0700001	Htra1	NM_01956	"Mus musc	RefSeq	7.914161	6.345401	2.966496	966
TC0600001	Irak2	NM_00111	"Mus musc	RefSeq	6.266698	7.834794	-2.96513	967
TC0900002	Ephb1	NM_17344	"Mus musc	RefSeq	4.905064	6.473153	-2.96512	968
TC1800001	Slc39a6	NM_13914	"Mus musc	RefSeq	7.861807	6.29418	2.964168	969
TC0300002	Ptgfrn	NM_01119	"Mus musc	RefSeq	9.322551	7.756816	2.960283	970
TC0700004	Syt17	NM_13864	"Mus musc	RefSeq	6.26154	4.698905	2.953929	971
TC0400004	H6pd	NM_17337	"Mus musc	RefSeq	7.69841	6.135895	2.953683	972
TC1100003	Plxdc1	NM_02819	"Mus musc	RefSeq	7.507093	5.944895	2.953034	973
TC0500002	Tec	NM_00111	"Mus musc	RefSeq	4.178143	5.740018	-2.95237	974
TC010000C	Casp8	NM_00981	"Mus musc	RefSeq	7.605916	9.167214	-2.95119	975
TC140000C	Arhgef40	NM_19824	"Mus musc	RefSeq	7.745076	6.18434	2.950043	976
TC0400001	Errfi1	NM_13375	"Mus musc	RefSeq	9.198475	7.637915	2.949683	977
TC0700001	Ampd3	NM_00127	"Mus musc	RefSeq	6.091175	7.65165	-2.94951	978
TC1700001	Rhoq	NM_14549	"Mus musc	RefSeq	8.414844	6.854675	2.948884	979
TC0600002	Magi1	NM_00108	"Mus musc	RefSeq	6.498566	8.05838	-2.94816	980
TC1400001	3632451O(	NM_02614	"Mus musc	RefSeq	4.781704	3.225022	2.941765	981
TC060000C	Cav1	NM_00761	"Mus musc	RefSeq	9.212177	10.76863	-2.9413	982
TC0500003	Oas1g	NM_01185	"Mus musc	RefSeq	4.934984	6.489488	-2.93733	983
TC0600001	Gata2	NM_00809	"Mus musc	RefSeq	7.163285	8.717185	-2.9361	984
TC0X00001	Arhgap6	NM_00970	"Mus musc	RefSeq	5.872461	4.321313	2.930502	985
TC030000C	Pld1	NM_00116	"Mus musc	RefSeq	7.492673	5.942098	2.929339	986

TC1400001	Sncg	NM_01143	"Mus musc	RefSeq	5.816465	7.366869	-2.92899	987
TC0800001	Ces2e	NM_17275	"Mus musc	RefSeq	4.355208	5.905065	-2.92788	988
TC0300000	Fat4	NM_18322	"Mus musc	RefSeq	7.54505	9.09476	-2.92758	989
TC0500002	Lgi2	NM_14494	"Mus musc	RefSeq	5.20602	3.656459	2.927281	990
TC0800000	Rnf150	NM_17737	"Mus musc	RefSeq	6.104361	4.55579	2.925272	991
TC1500000	Tbc1d22a	NM_14547	"Mus musc	RefSeq	6.705297	8.252392	-2.92228	992
TC0400000	Tlr4	NM_02129	"Mus musc	RefSeq	7.967988	6.422933	2.918152	993
TC0900001	Als2cl	NM_00114	"Mus musc	RefSeq	6.188763	7.731564	-2.9136	994
TC0400002	Gm10595	ENSMUST0	cdna:know	ENSEMBL	5.314084	3.771384	2.913392	995
TC0400002	Gm10595	ENSMUST0	cdna:know	ENSEMBL	5.314084	3.771384	2.913392	995
TC0700000	Klk8	NM_00894	"Mus musc	RefSeq	5.254917	6.796293	-2.91072	997
TC0900000	Cyp1a1	NM_00999	"Mus musc	RefSeq	4.542322	6.082863	-2.90904	998
TC0900000	Igdcc4	NM_02004	"Mus musc	RefSeq	6.783769	5.245119	2.905225	999
TC1700002	H2-Q8	NM_02312	"Mus musc	RefSeq	8.351861	9.888749	-2.90168	1000
TC0200002	Bmp2	NM_00755	"Mus musc	RefSeq	6.612502	5.076473	2.899952	1001
TC1400001	Hr	NM_02187	"Mus musc	RefSeq	7.118309	5.582542	2.899425	1002
TC0500002	Tgfbr3	NM_01157	"Mus musc	RefSeq	9.359439	7.824021	2.898724	1003
TC0400000	Gm10594	ENSMUST0	cdna:know	ENSEMBL	5.225224	3.691077	2.896171	1005
TC0400002	Gm10594	ENSMUST0	cdna:know	ENSEMBL	5.225224	3.691077	2.896171	1005
TC4_GL456	Gm10594	ENSMUST0	cdna:know	ENSEMBL	5.225224	3.691077	2.896171	1005
TC4_JH584	Gm10594	ENSMUST0	cdna:know	ENSEMBL	5.225224	3.691077	2.896171	1005
TC4_JH584	Gm10590	ENSMUST0	cdna:know	ENSEMBL	5.263843	3.729696	2.896171	1008
TC0700002	4931406P1	NM_17274	"Mus musc	RefSeq	8.057367	9.589774	-2.89268	1009
TC1000002	Gipc3	NM_14895	"Mus musc	RefSeq	6.580234	8.111806	-2.89101	1010
TC1100004	4-Sep	ENSMUST0	cdna:novel	ENSEMBL	7.391851	8.923083	-2.89033	1011
TC1400000	Gm8297	XM_98607	"PREDICTEI	RefSeq	4.522963	6.054146	-2.89023	1012
TC0500000	Arhgap24	NM_02927	"Mus musc	RefSeq	5.618282	4.087191	2.890043	1013
TC1400001	Fam107a	NM_18318	"Mus musc	RefSeq	5.473089	7.00375	-2.88918	1014
TC0300002	Enpep	NM_00793	"Mus musc	RefSeq	6.527431	4.997041	2.888639	1015
TC0200004	Itpripl1	NM_00116	"Mus musc	RefSeq	7.097944	8.627897	-2.88776	1016
TC1500000	Nov	NM_01093	"Mus musc	RefSeq	7.53196	6.003428	2.884921	1017
TC0800003	Sipa1l2	NM_00108	"Mus musc	RefSeq	6.682895	8.211067	-2.8842	1018
TC1500002	Fmnl3	NM_01171	"Mus musc	RefSeq	7.301932	8.828128	-2.88025	1019
TC0100000	Adhfe1	NM_17523	"Mus musc	RefSeq	6.117376	4.592532	2.877556	1020
TC0500002	Atp8a1	NM_00972	"Mus musc	RefSeq	5.996903	7.519513	-2.8731	1021
TC1000002	Slc41a2	NM_17738	"Mus musc	RefSeq	5.930877	4.409778	2.870096	1022
TC0400001	Camk2n1	NM_02545	"Mus musc	RefSeq	8.544685	7.023691	2.869887	1023
TC0900003	Dync2h1	NM_02985	"Mus musc	RefSeq	5.076761	3.555884	2.869654	1024
TC0900000	Pygo1	NM_02811	"Mus musc	RefSeq	6.2804	4.762161	2.864412	1025
TC1100003	Leprel4	NM_17683	"Mus musc	RefSeq	7.303692	5.785715	2.863892	1026
TC1000001	Avpr1a	NM_01684	"Mus musc	RefSeq	6.490402	4.973581	2.861598	1027
TC0500002	Sgcb	NM_01189	"Mus musc	RefSeq	8.86336	7.348142	2.85842	1028
TC0400000	Gm2457	XM_00147	"PREDICTEI	RefSeq	6.594961	5.083349	2.851285	1029
TC0600000	Adcyap1r1	NM_00740	"Mus musc	RefSeq	6.974707	5.463736	2.850018	1030
TC1800000	Megf10	NM_00100	"Mus musc	RefSeq	6.26651	4.757785	2.845584	1031
TC1900001	Fam189a2	NM_00111	"Mus musc	RefSeq	6.996509	8.504527	-2.84419	1032
TC0200005	Ppp1r3d	NM_00108	"Mus musc	RefSeq	4.178982	5.682839	-2.836	1033

TC0200001	Lrp4	NM_00114	"Mus musc	RefSeq	6.971915	5.468569	2.834995	1034
TC010000C	Pnkd	NM_02558	"Mus musc	RefSeq	7.07475	8.577857	-2.83453	1035
TC030000C	S100a7a	NM_19942	"Mus musc	RefSeq	5.300861	3.799025	2.832029	1036
TC0900001	Irak1bp1	NM_00116	"Mus musc	RefSeq	4.953971	3.452493	2.831326	1037
TC0300001	Tspan2	NM_02753	"Mus musc	RefSeq	6.659598	8.159578	-2.82839	1038
TC1600001	Ets2	NM_01180	"Mus musc	RefSeq	7.780233	9.277054	-2.8222	1039
TC0400002	Gm12394	ENSMUST0	cdna:putat	ENSEMBL	5.228189	3.733004	2.819003	1040
TC0300001	Gbp7	NM_00108	"Mus musc	RefSeq	8.573057	10.06675	-2.8161	1041
TC0500001	Ephb4	NM_01014	"Mus musc	RefSeq	7.533905	9.025562	-2.81212	1042
TC0700004	Nmb	NM_02652	"Mus musc	RefSeq	6.256841	4.765235	2.812018	1043
TC190000C	Syt7	NM_01880	"Mus musc	RefSeq	5.927174	7.418041	-2.81058	1044
TC0300001	Slc16a4	NM_14613	"Mus musc	RefSeq	3.577744	5.067716	-2.80884	1045
TC090000C	Casp12	NM_00980	"Mus musc	RefSeq	7.376622	5.887087	2.807985	1046
TC1300002	Dbn1	NM_00117	"Mus musc	RefSeq	7.96376	6.474666	2.807126	1047
TC130000C	Rnf144b	NM_14604	"Mus musc	RefSeq	5.971629	7.46033	-2.80636	1048
TC0100003	Mndal	NM_00117	"Mus musc	RefSeq	7.596655	9.085163	-2.80599	1049
TC4_JH584	CR974586.1	ENSMUST0	cdna:novel	ENSEMBL	6.498175	5.011811	2.801819	1050
TC080000C	Jak3	NM_00119	"Mus musc	RefSeq	6.824092	8.310409	-2.80173	1051
TC0900001	Snrk	NM_00116	"Mus musc	RefSeq	8.259458	9.745562	-2.80132	1052
TC0X0000C	Efnb1	NM_01011	"Mus musc	RefSeq	7.103213	8.588596	-2.79992	1053
TC1500002	Mapk11	NM_01116	"Mus musc	RefSeq	6.633327	8.116742	-2.7961	1054
TC1000001	Eya4	NM_01016	"Mus musc	RefSeq	5.023407	3.540439	2.795232	1055
TC050000C	Fam114a1	NM_02666	"Mus musc	RefSeq	8.432259	6.949823	2.794201	1056
TC1100003	Aspa	NM_02311	"Mus musc	RefSeq	5.433582	3.952756	2.791085	1057
TC1300001	Ssbp2	NM_02427	"Mus musc	RefSeq	6.132786	4.65406	2.787025	1058
TC010000C	Dgkd	NM_17764	"Mus musc	RefSeq	6.586579	8.064468	-2.78541	1059
TC010000C	Nop58	NM_01886	"Mus musc	RefSeq	9.104267	7.627579	2.783091	1060
TC0200004	Hdc	NM_00823	"Mus musc	RefSeq	4.478417	5.954073	-2.7811	1061
TC0800001	Adam9	NM_00127	"Mus musc	RefSeq	9.344659	7.869961	2.779255	1062
TC1300001	Net1	NM_00104	"Mus musc	RefSeq	5.645814	7.119749	-2.77779	1063
TC0700001	Rgma	NM_17774	"Mus musc	RefSeq	7.627242	6.154235	2.775999	1064
TC180000C	9430020KC	NM_00108	"Mus musc	RefSeq	8.713583	10.18437	-2.77173	1065
TC1300002	Naip5	NM_01087	"Mus musc	RefSeq	3.647199	5.117304	-2.77042	1066
TC020000C	Rapgef1	NM_00103	"Mus musc	RefSeq	8.426034	9.893472	-2.7653	1067
TC1300001	Esm1	NM_02361	"Mus musc	RefSeq	4.447372	5.912773	-2.7614	1068
TC130000C	Mef2c	NM_02528	"Mus musc	RefSeq	6.856642	8.321401	-2.76017	1069
TC140000C	Anxa8	NM_01347	"Mus musc	RefSeq	6.158261	4.694408	2.758441	1070
TC1400002	Dzip1	NM_02594	"Mus musc	RefSeq	5.690126	4.227005	2.757042	1071
TC050000C	Tcf23	NM_05308	"Mus musc	RefSeq	4.610303	6.072983	-2.7562	1072
TC1400002	Uggt2	NM_00108	"Mus musc	RefSeq	5.193124	3.730687	2.755735	1073
TC1400001	Lrrc3b	NM_14605	"Mus musc	RefSeq	4.697461	6.159046	-2.75411	1074
TC190000C	Map3k11	NM_02201	"Mus musc	RefSeq	6.926397	8.387231	-2.75267	1075
TC1600001	Tmem44	NM_17261	"Mus musc	RefSeq	5.438745	6.897276	-2.74828	1076
TC0800002	Fhod1	NM_17769	"Mus musc	RefSeq	5.828052	7.286454	-2.74804	1077
TC150000C	Atxn10	NM_01684	"Mus musc	RefSeq	8.772753	7.314744	2.74729	1078
TC0X00003	Gm614	NM_00103	"Mus musc	RefSeq	3.691252	5.147623	-2.74417	1079
TC1000001	Cdk17	NM_14623	"Mus musc	RefSeq	6.732894	8.189202	-2.74405	1080

TC0200001	Duox1	NM_00109	"Mus musc	RefSeq	5.991851	4.535947	2.743284	1081
TC1900000	Slc1a1	NM_00919	"Mus musc	RefSeq	4.811889	6.267454	-2.74264	1082
TC0800002	Nfix	NM_00108	"Mus musc	RefSeq	9.168775	7.714357	2.74046	1083
TC0700002	Atf5	NM_03069	"Mus musc	RefSeq	7.104843	5.650884	2.739588	1084
TC1600000	Jam2	NM_02384	"Mus musc	RefSeq	9.063138	10.51666	-2.73876	1085
TC0300001	Fndc3b	NM_17318	"Mus musc	RefSeq	9.602121	8.148906	2.738176	1086
TC1000001	Ulbp1	NM_02997	"Mus musc	RefSeq	4.278489	5.73145	-2.73769	1087
TC1700002	Sema6b	NM_01366	"Mus musc	RefSeq	6.918495	8.370701	-2.73626	1088
TC1800000	Gramd3	NM_02624	"Mus musc	RefSeq	6.097492	4.645729	2.735421	1089
TC0200000	Tanc1	NM_19829	"Mus musc	RefSeq	6.959259	8.4108	-2.735	1090
TC0300002	Plekho1	NM_02332	"Mus musc	RefSeq	7.297125	8.748565	-2.73481	1091
TC0500000	Cc2d2a	NM_17227	"Mus musc	RefSeq	7.155373	5.703995	2.734691	1092
TC0X00000	Stard8	NM_19901	"Mus musc	RefSeq	6.02741	7.477302	-2.73188	1093
TC0200005	Rbl1	NM_01124	"Mus musc	RefSeq	4.900485	6.34946	-2.73014	1094
TC0200005	Zbtb46	NM_02765	"Mus musc	RefSeq	5.349099	6.79695	-2.72801	1095
TC1000000	Plekhg1	NM_00115	"Mus musc	RefSeq	7.647899	9.095233	-2.72704	1096
TC0X00002	Cysltr1	NM_02147	"Mus musc	RefSeq	3.237849	4.684961	-2.72662	1097
TC1800000	Snx24	NM_02939	"Mus musc	RefSeq	6.313244	4.86795	2.723183	1098
TC1100001	Chad	NM_00768	"Mus musc	RefSeq	5.66051	4.215249	2.723121	1099
TC1000000	Txlnb	NM_13862	"Mus musc	RefSeq	5.527206	6.9719	-2.72205	1100
TC1300002	Thbs4	NM_01158	"Mus musc	RefSeq	6.689711	5.246705	2.718868	1101
TC0500001	P2rx4	NM_01102	"Mus musc	RefSeq	7.58278	6.140289	2.717897	1102
TC0500003	Pitpnm2	NM_00128	"phosphati	RefSeq	6.827787	8.269032	-2.71555	1103
TC1200001	Vipr2	NM_00951	"Mus musc	RefSeq	6.461664	5.021299	2.713895	1104
TC1200001	Trib2	NM_14455	"Mus musc	RefSeq	6.818474	8.258452	-2.71317	1105
TC1200000	Gm527	NM_00102	"Mus musc	RefSeq	6.88284	5.443248	2.712441	1106
TC0500001	Lrrc8c	NM_13389	"Mus musc	RefSeq	8.834443	10.27259	-2.70973	1107
TC0700001	Gdpd5	NM_20135	"Mus musc	RefSeq	6.202955	7.640567	-2.70872	1108
TC0100002	Rftn2	NM_02871	"Mus musc	RefSeq	6.566813	5.129978	2.707263	1109
TC0100003	G0s2	NM_00805	"Mus musc	RefSeq	7.487243	6.05077	2.706584	1110
TC1300002	Map3k1	NM_01194	"Mus musc	RefSeq	7.015379	8.451736	-2.70637	1111
TC0200001	Fgf7	NM_00800	"Mus musc	RefSeq	4.664832	3.22963	2.7042	1112
TC1300002	Naip2	NM_01087	"Mus musc	RefSeq	3.681323	5.112694	-2.69703	1113
TC1600001	Klhl6	NM_18339	"Mus musc	RefSeq	5.715216	7.146586	-2.69703	1114
TC1400000	Gm3029	XM_00147	"PREDICTEI	RefSeq	4.864952	6.296207	-2.69681	1115
TC0200005	D630003M	NM_00113	"Mus musc	RefSeq	6.759216	5.329302	2.694307	1116
TC1400001	Tspan14	NM_14592	"Mus musc	RefSeq	7.106621	8.535101	-2.69163	1117
TC1100003	Myo1d	NM_17739	"Mus musc	RefSeq	7.679838	9.106134	-2.68756	1118
TC0300001	Adh1	NM_00740	"Mus musc	RefSeq	7.838734	9.264851	-2.68723	1119
TC0600000	Gpnmb	NM_05311	"Mus musc	RefSeq	6.011962	4.586559	2.685895	1120
TC0100003	Dnm3	NM_00103	"Mus musc	RefSeq	5.860811	7.286146	-2.68577	1121
TC0800002	Dok4	NM_05324	"Mus musc	RefSeq	6.711567	8.136166	-2.6844	1122
TC0600001	Fbln2	NM_00799	"Mus musc	RefSeq	10.53871	9.114708	2.683296	1123
TC0900001	Panx1	NM_01948	"Mus musc	RefSeq	6.336359	4.914637	2.679051	1124
TC0900000	Cryab	NM_00128	"crystallin,	RefSeq	8.481207	7.060393	2.677365	1125
TC1700002	Slc8a1	NM_01140	"Mus musc	RefSeq	5.219142	3.798464	2.677113	1126
TC0100003	Soat1	NM_00923	"Mus musc	RefSeq	7.317954	5.898466	2.674906	1127

TC0400003	Zfp69	NM_00100	"Mus musc	RefSeq	5.686674	7.10541	-2.67351	1128
TC1200000	Lamb1	NM_00848	"Mus musc	RefSeq	9.963551	8.546552	2.670295	1129
TC0200000	Fam102a	NM_15356	"Mus musc	RefSeq	7.303441	8.72004	-2.66955	1130
TC0400001	Fabp3	NM_01017	"Mus musc	RefSeq	7.118409	8.528785	-2.65806	1131
TC0900001	Ccbp2	NM_02160	"Mus musc	RefSeq	7.061183	5.651378	2.657012	1132
TC0600000	Rtkn	NM_00113	"Mus musc	RefSeq	6.702592	5.292946	2.65672	1133
TC0500003	Cmklr1	NM_00815	"Mus musc	RefSeq	7.862178	6.453461	2.655009	1134
TC0500002	Prom1	NM_00116	"Mus musc	RefSeq	3.384234	4.790299	-2.65013	1135
TC0700001	Pde3b	NM_01105	"Mus musc	RefSeq	4.996758	3.590693	2.650133	1135
TC0800001	Wfdc1	NM_02339	"Mus musc	RefSeq	6.731255	5.326764	2.647244	1137
TC0500002	Fryl	NM_02819	"Mus musc	RefSeq	7.410501	8.813707	-2.64489	1138
TC0900001	Dock6	NM_17703	"Mus musc	RefSeq	7.782488	9.184577	-2.64284	1139
TC1900000	Rasgrp2	NM_01124	"Mus musc	RefSeq	6.406621	7.807513	-2.64065	1140
TC0900000	Usp2	NM_19809	"Mus musc	RefSeq	6.652638	5.253413	2.637599	1141
TC1700001	Dync2li1	NM_17225	"Mus musc	RefSeq	6.499265	5.100694	2.636403	1142
TC1700001	Ltbp1	NM_01991	"Mus musc	RefSeq	8.493542	7.095114	2.636142	1143
TC0200000	B3galt1	NM_02028	"Mus musc	RefSeq	4.97519	3.577369	2.635033	1144
TC1200001	Scin	NM_00114	"Mus musc	RefSeq	5.098006	3.700494	2.634469	1145
TC1400001	Gm3685	XM_00147	"PREDICTEI	RefSeq	3.663879	5.06064	-2.6331	1146
TC0200003	Sestd1	NM_17546	"Mus musc	RefSeq	7.867025	6.470332	2.632973	1147
TC0300002	Myoz2	NM_02150	"Mus musc	RefSeq	5.727134	7.123461	-2.63231	1148
TC1400001	Kcnk5	NM_02154	"Mus musc	RefSeq	5.528655	4.1326	2.631809	1149
TC0500001	Fscn1	NM_00798	"Mus musc	RefSeq	7.400439	8.79625	-2.63136	1150
TC0900003	Nbeal2	NM_18327	"Mus musc	RefSeq	5.866152	7.260537	-2.62877	1151
TC0800001	Car7	NM_05307	"Mus musc	RefSeq	6.414526	7.808644	-2.62828	1152
TC0700004	Igf2	NM_00112	"Mus musc	RefSeq	6.218912	7.61292	-2.62808	1153
TC1200001	Prkar2b	NM_01115	"Mus musc	RefSeq	5.863514	4.47013	2.626941	1154
TC0700003	Cpeb1	NM_00775	"Mus musc	RefSeq	5.60923	4.215858	2.62692	1155
TC0100000	Fam117b	NM_00103	"Mus musc	RefSeq	7.615116	9.00838	-2.62672	1156
TC0X00000	Fhl1	NM_01021	"Mus musc	RefSeq	8.479943	7.087268	2.625651	1157
TC0X00000	Aff2	NM_00803	"Mus musc	RefSeq	5.264736	3.872234	2.625336	1158
TC1100001	Trim25	NM_00954	"Mus musc	RefSeq	7.03963	8.43209	-2.62526	1159
TC0200002	Id1	NM_01049	"Mus musc	RefSeq	9.248682	10.64104	-2.62507	1160
TC0100001	Plxna2	NM_00888	"Mus musc	RefSeq	7.971632	9.363466	-2.62412	1161
TC0300001	Arhgap29	NM_17252	"Mus musc	RefSeq	8.112219	9.503634	-2.62336	1162
TC1400001	Gm3542	ENSMUST0	cdna:putat	ENSEMBL	4.231548	5.622326	-2.6222	1163
TC1400001	Gm9603	ENSMUST0	cdna:putat	ENSEMBL	4.231548	5.622326	-2.6222	1163
TC1300000	Akr1c19	NM_00101	"Mus musc	RefSeq	3.826478	5.216692	-2.62118	1165
TC0400003	Nipal3	NM_02899	"Mus musc	RefSeq	6.0082	7.397696	-2.61987	1166
TC0500002	Stk32b	NM_02241	"Mus musc	RefSeq	6.097983	4.70894	2.619049	1167
TC0900003	Stt3b	NM_02422	"Mus musc	RefSeq	9.123231	7.73497	2.61763	1168
TC0400002	Mup19	NM_00113	"Mus musc	RefSeq	5.077458	3.689318	2.61741	1169
TC0600003	Ntf3	NM_00116	"Mus musc	RefSeq	5.08039	6.46283	-2.60709	1170
TC0600002	Ptn	NM_00897	"Mus musc	RefSeq	5.62877	4.246938	2.605991	1171
TC0200003	Fkbp7	NM_01022	"Mus musc	RefSeq	7.099061	5.719017	2.602763	1172
TC0700003	Man2a2	NM_17290	"Mus musc	RefSeq	8.207996	6.829379	2.60019	1173
TC1300001	Plk2	NM_15280	"Mus musc	RefSeq	9.382307	10.76066	-2.59971	1174

TC1200001	Ifrd1	NM_01356	"Mus musc	RefSeq	8.923821	7.545719	2.599262	1175
TC0200001	Olfr1033	NM_14657	"Mus musc	RefSeq	4.217257	2.840442	2.596944	1176
TC0700002	Scn1b	NM_01132	"Mus musc	RefSeq	8.233791	6.857282	2.596393	1177
TC1400001	Bmp4	NM_00755	"Mus musc	RefSeq	9.077081	7.703006	2.592017	1178
TC1900001	Sgms1	NM_00116	"Mus musc	RefSeq	6.5512	7.923074	-2.58807	1179
TC1100004	Cyth1	NM_00111	"Mus musc	RefSeq	7.036789	8.408595	-2.58794	1180
TC0600000	Abcg2	NM_01192	"Mus musc	RefSeq	6.544433	7.914926	-2.58559	1181
TC1200001	Hdac9	NM_00127	"Mus musc	RefSeq	4.57896	5.949164	-2.58507	1182
TC1100000	Wscd1	NM_17761	"Mus musc	RefSeq	5.826596	7.195645	-2.583	1183
TC0600003	Hebp1	NM_01354	"Mus musc	RefSeq	6.204073	4.835349	2.582421	1184
TC1700001	Dll1	NM_00786	"Mus musc	RefSeq	5.711252	7.07994	-2.58236	1185
TC0700002	Cpt1c	NM_15367	"Mus musc	RefSeq	6.383777	5.017793	2.577521	1186
TC1500000	Ptp4a3	NM_00116	"Mus musc	RefSeq	7.481096	8.846176	-2.57591	1187
TC0X00003	Mospd2	NM_02973	"Mus musc	RefSeq	6.189667	7.552004	-2.57101	1188
TC0300001	Gbp5	NM_15356	"Mus musc	RefSeq	5.873593	7.235551	-2.57034	1189
TC0100003	Cadm3	NM_05319	"Mus musc	RefSeq	6.235504	4.873754	2.569967	1190
TC0900001	9230110C1	NM_19901	"Mus musc	RefSeq	5.001237	3.64134	2.566669	1191
TC0100003	Fmo1	NM_01023	"Mus musc	RefSeq	8.539798	9.8996	-2.5665	1192
TC0500000	Pi4k2b	NM_02595	"Mus musc	RefSeq	5.445465	4.087033	2.564064	1193
TC0800002	Fam149a	NM_15353	"Mus musc	RefSeq	7.147619	5.789741	2.563079	1194
TC1200001	Kif26a	NM_00109	"Mus musc	RefSeq	6.634831	7.992425	-2.56258	1195
TC1400002	Efs	NM_01011	"Mus musc	RefSeq	6.840026	5.482761	2.56199	1196
TC1900000	Tmem2	NM_00103	"Mus musc	RefSeq	5.953046	7.31002	-2.56147	1197
TC0200004	Tspan18	NM_18318	"Mus musc	RefSeq	7.787935	9.142917	-2.55794	1198
TC1000002	Fabp3-ps1	ENSMUST0	cdna:know	ENSEMBL	7.246328	8.599867	-2.55538	1199
TC0400002	Mup15	NM_00120	"Mus musc	RefSeq	4.684852	3.331409	2.555212	1200
TC0300000	Phf17	NM_00113	"Mus musc	RefSeq	8.08544	6.732187	2.554876	1201
TC0100003	Mnda	NM_00103	"Mus musc	RefSeq	8.604754	7.251709	2.554507	1202
TC1300000	Mboat1	NM_15354	"Mus musc	RefSeq	5.07572	3.722791	2.554302	1203
TC0800002	Cdh11	NM_00986	"Mus musc	RefSeq	7.278288	5.92547	2.554105	1204
TC1000001	Ndufa4l2	NM_00109	"Mus musc	RefSeq	7.743089	6.390498	2.553703	1205
TC1900000	Ctsf	NM_01986	"Mus musc	RefSeq	7.338468	5.985928	2.553613	1206
TC1800001	Adrb2	NM_00742	"Mus musc	RefSeq	6.36962	7.721752	-2.55289	1207
TC1900000	Tmem252	NM_18316	"Mus musc	RefSeq	4.856931	6.208891	-2.55259	1208
TC1800000	Lims2	NM_14486	"Mus musc	RefSeq	9.669333	11.02029	-2.55081	1209
TC0700001	Fzd4	NM_00805	"Mus musc	RefSeq	8.618806	9.969599	-2.55052	1210
TC0200002	Wisp2	NM_01687	"Mus musc	RefSeq	7.335703	5.985163	2.550076	1211
TC0X00000	AA414768	NM_00127	"Mus musc	RefSeq	4.663055	3.31453	2.546516	1212
TC0900000	Casp4	NM_00760	"Mus musc	RefSeq	7.651723	6.303265	2.546398	1213
TC0300001	Ttll7	NM_02759	"Mus musc	RefSeq	5.550891	6.898037	-2.54408	1214
TC1900000	Atrnl1	NM_18141	"Mus musc	RefSeq	8.912566	7.566341	2.54246	1215
TC0700001	Pak1	NM_01103	"Mus musc	RefSeq	5.175825	3.830305	2.541218	1216
TC0Y00000	G530011O	ENSMUST0	cdna:know	ENSEMBL	9.114132	7.769188	2.540203	1217
TC0Y00000	G530011O	ENSMUST0	cdna:know	ENSEMBL	9.114132	7.769188	2.540203	1217
TC0400003	Zdhhc18	NM_00101	"Mus musc	RefSeq	7.173399	8.518315	-2.54015	1219
TC1400002	Gm21560	XM_00368	"PREDICTEI	RefSeq	4.206519	5.550212	-2.538	1220
TC1400002	Gm3512	ENSMUST0	cdna:putat	ENSEMBL	5.57209	6.915189	-2.53696	1221

TC1500001	Trps1	NM_03200	"Mus musc	RefSeq	7.124343	5.781597	2.536336	1222
TC0700003	Ntrk3	NM_00874	"Mus musc	RefSeq	5.475889	4.133208	2.536222	1223
TC0400000	Mmp16	NM_01972	"Mus musc	RefSeq	4.839577	3.497661	2.534877	1224
TC1400000	Gm8159	XM_98454	"PREDICTEI	RefSeq	4.49736	5.839219	-2.53478	1225
TC1300002	Ror2	NM_01384	"Mus musc	RefSeq	6.253315	4.911688	2.53437	1226
TC1700000	Mllt4	NM_01080	"Mus musc	RefSeq	7.832149	9.173104	-2.53319	1227
TC0700001	Ptpre	NM_01121	"Mus musc	RefSeq	4.941361	6.281212	-2.53125	1228
TC0100000	Cxcr7	NM_00772	"Mus musc	RefSeq	8.680507	7.34072	2.531139	1229
TC1000000	Gm5423	ENSMUST0	cdna:know	ENSEMBL	6.64454	7.983307	-2.52935	1230
TC0700003	Trim30a	NM_00909	"Mus musc	RefSeq	6.695313	8.033732	-2.52874	1231
TC1300002	Hexb	NM_01042	"Mus musc	RefSeq	8.121444	6.783034	2.528725	1232
TC0700001	Olfr550	ENSMUST0	cdna:know	ENSEMBL	5.483721	4.145644	2.528141	1233
TC1100004	Rhbdf2	NM_00116	"Mus musc	RefSeq	6.479123	7.817185	-2.52812	1234
TC1000002	Srgn	NM_01115	"Mus musc	RefSeq	7.423793	8.761301	-2.52714	1235
TC0400001	Ctnnbip1	NM_02346	"Mus musc	RefSeq	7.461148	8.798129	-2.52622	1236
TC0100002	Igfbp5	NM_01051	"Mus musc	RefSeq	8.520207	7.184016	2.524838	1237
TC0700004	Pik3c2a	NM_01108	"Mus musc	RefSeq	6.938466	8.273916	-2.52354	1238
TC1400002	Myh6	NM_01085	"Mus musc	RefSeq	6.428762	7.763106	-2.52161	1239
TC0800000	Comp	NM_01668	"Mus musc	RefSeq	6.81101	5.476804	2.521367	1240
TC1100001	Mmd	NM_02617	"Mus musc	RefSeq	4.408295	5.738851	-2.515	1241
TC1300000	Sfrp4	NM_01668	"Mus musc	RefSeq	5.095708	3.766472	2.512696	1242
TC1000002	Slc16a10	NM_00111	"Mus musc	RefSeq	5.654632	4.325491	2.51253	1243
TC1100001	Rtn4rl1	NM_17770	"Mus musc	RefSeq	6.241754	4.915729	2.507109	1244
TC1700002	Fez2	NM_00128	"fasciculati	RefSeq	7.7505	9.076433	-2.50695	1245
TC0400003	Ptprf	NM_01121	"Mus musc	RefSeq	6.334694	5.00911	2.506343	1246
TC0800000	Zfp703	NM_00111	"Mus musc	RefSeq	7.465322	6.141486	2.503308	1247
TC0200000	Acvr2a	NM_00739	"Mus musc	RefSeq	6.287195	7.610991	-2.50324	1248
TC0900000	Ldlr	NM_01070	"Mus musc	RefSeq	7.519634	6.195896	2.503138	1249
TC0700004	Vkorc1	NM_17860	"Mus musc	RefSeq	9.64973	8.327067	2.501274	1250
TC0200004	1110051M	NM_17512	"Mus musc	RefSeq	5.357374	4.034951	2.500858	1251
TC4_JH584	CR974586..	ENSMUST0	cdna:novel	ENSEMBL	7.137424	5.815993	2.499139	1252
TC0600000	Ggcx	NM_01980	"Mus musc	RefSeq	8.769258	7.449622	2.496031	1253
TC1100004	Igtp	NM_01873	"Mus musc	RefSeq	7.245449	8.562299	-2.49122	1254
TC1000002	Slc35e3	NM_02987	"Mus musc	RefSeq	7.213276	5.897471	2.489412	1255
TC0100001	Slc30a1	NM_00957	"Mus musc	RefSeq	6.781169	8.09685	-2.4892	1256
TC0900002	Sorl1	NM_01143	"Mus musc	RefSeq	5.667287	4.3521	2.488346	1257
TC1200000	Rgs6	NM_01581	"Mus musc	RefSeq	6.060283	4.745337	2.48793	1258
TC1600001	Parp14	NM_00103	"Mus musc	RefSeq	7.122589	8.436976	-2.48697	1259
TC1000000	Pln	NM_00114	"Mus musc	RefSeq	6.717557	8.029895	-2.48344	1260
TC1800001	Tmem173	NM_02826	"Mus musc	RefSeq	7.144464	8.456651	-2.48318	1261
TC1600002	Tiam1	NM_00114	"Mus musc	RefSeq	6.030474	4.719602	2.480914	1262
TC0400004	Mup7	NM_00113	"Mus musc	RefSeq	5.203318	3.893007	2.47995	1263
TC1000001	Wif1	NM_01191	"Mus musc	RefSeq	6.274677	4.964427	2.479845	1264
TC1100003	Nlk	NM_00870	"Mus musc	RefSeq	6.501705	7.81142	-2.47893	1265
TC0400001	Casz1	NM_02719	"Mus musc	RefSeq	5.619552	6.928034	-2.47681	1266
TC1100000	Grap	NM_02781	"Mus musc	RefSeq	6.484347	7.792716	-2.47661	1267
TC0700003	Capn5	NM_00760	"Mus musc	RefSeq	7.042232	5.734022	2.476341	1268

TC0100000	Gmppa	NM_13370	"Mus musc	RefSeq	7.971966	6.664798	2.474553	1269
TC1200001	Rnf144a	NM_08056	"Mus musc	RefSeq	7.098525	8.404927	-2.47324	1270
TC1900001	Asah2	NM_01883	"Mus musc	RefSeq	6.675326	7.981274	-2.47246	1271
TC0200003	Dpp7	NM_03184	"Mus musc	RefSeq	6.715485	5.410227	2.471279	1272
TC1700002	Vegfa	NM_00111	"Mus musc	RefSeq	8.171797	6.866972	2.470538	1273
TC0100000	Sp100	NM_01367	"Mus musc	RefSeq	7.573253	8.877803	-2.47007	1274
TC1300001	Adamts6	NM_00108	"Mus musc	RefSeq	5.93883	4.634394	2.469872	1275
TC1200002	Ltbp2	NM_01358	"Mus musc	RefSeq	6.687672	5.383905	2.468726	1276
TC1000001	Utrn	NM_01168	"Mus musc	RefSeq	8.202138	9.504841	-2.46691	1277
TC0X00002	G6pdx	NM_00806	"Mus musc	RefSeq	8.382437	7.079802	2.46679	1278
TC1900000	Casp7	NM_00761	"Mus musc	RefSeq	6.40384	7.70617	-2.46627	1279
TC0500002	Gbp11	NM_00103	"Mus musc	RefSeq	7.027078	8.329292	-2.46607	1280
TC1200001	Ralgapa1	NM_00111	"Mus musc	RefSeq	7.67013	8.972285	-2.46597	1281
TC0700000	Pnmal2	NM_00109	"Mus musc	RefSeq	5.542528	4.241733	2.463646	1282
TC1900000	Hhex	NM_00824	"Mus musc	RefSeq	4.390415	5.690851	-2.46303	1283
TC0400003	Nkain1	NM_02599	"Mus musc	RefSeq	6.071475	4.77131	2.46257	1284
TC0300002	Gucy1b3	NM_01746	"Mus musc	RefSeq	7.938119	6.638063	2.462384	1285
TC0300000	Sfrp2	NM_00914	"Mus musc	RefSeq	7.012663	5.713011	2.461695	1286
TC0500003	Gbp8	NM_02950	"Mus musc	RefSeq	5.891966	7.191434	-2.46138	1287
TC0100002	Rnf152	NM_00116	"Mus musc	RefSeq	4.455218	5.754499	-2.46106	1288
TC1800000	Zadh2	NM_14609	"Mus musc	RefSeq	7.100507	5.801521	2.460559	1289
TC0X00001	Fancb	NM_17502	"Mus musc	RefSeq	3.953697	5.25195	-2.45931	1290
TC1900001	Stx3	NM_01150	"Mus musc	RefSeq	6.220345	7.518405	-2.45898	1291
TC0900002	Tmod2	NM_00103	"Mus musc	RefSeq	6.38246	5.084482	2.45884	1292
TC1000002	Adamtsl5	NM_00111	"Mus musc	RefSeq	6.749619	5.451931	2.458346	1293
TC1700001	Crim1	NM_01580	"Mus musc	RefSeq	7.743125	9.040258	-2.4574	1294
TC1300001	Epdr1	NM_13406	"Mus musc	RefSeq	5.764578	4.467551	2.45722	1295
TC0200002	Cds2	NM_13865	"Mus musc	RefSeq	8.093665	9.389254	-2.45477	1296
TC0X00002	Smarca1	NM_05312	"Mus musc	RefSeq	4.555386	3.261653	2.451616	1297
TC0300001	Gbp3	NM_00128	"guanylate	RefSeq	6.747841	8.040803	-2.45031	1298
TC0600003	Adipor2	NM_19798	"Mus musc	RefSeq	6.597518	7.889972	-2.44944	1299
TC1600000	Glis2	NM_03118	"Mus musc	RefSeq	7.833236	6.541195	2.448742	1300
TC0700000	Brsk1	NM_00116	"Mus musc	RefSeq	6.308505	5.0167	2.448342	1301
TC0400002	Ccl19	NM_01188	"Mus musc	RefSeq	5.713768	4.422644	2.447186	1302
TC0300001	Slc44a5	NM_00108	"Mus musc	RefSeq	3.331209	4.62206	-2.44672	1303
TC0X00001	Clcn5	NM_01669	"Mus musc	RefSeq	6.201264	4.91069	2.446254	1304
TC1500001	Fam84b	NM_00116	"Mus musc	RefSeq	6.17912	7.46839	-2.44404	1305
TC1400002	Ripk3	NM_00116	"Mus musc	RefSeq	5.962218	7.251438	-2.44396	1306
TC0900003	Uba7	NM_02373	"Mus musc	RefSeq	6.622426	7.911015	-2.44289	1307
TC0900000	Bace1	NM_01179	"Mus musc	RefSeq	7.129157	5.84075	2.442582	1308
TC1300000	Nid1	NM_01091	"Mus musc	RefSeq	11.18226	9.894317	2.4418	1309
TC0700004	Sigirr	NM_02305	"Mus musc	RefSeq	5.55439	6.842003	-2.44124	1310
TC0500003	2900026A0	NM_17288	"Mus musc	RefSeq	6.566119	7.852392	-2.43897	1311
TC1000002	Gstt2	NM_01036	"Mus musc	RefSeq	5.999972	7.285566	-2.43782	1312
TC0800001	Fam65a	NM_00108	"Mus musc	RefSeq	7.878545	9.16395	-2.43751	1313
TC1300002	Arsk	NM_02984	"Mus musc	RefSeq	7.357827	6.072738	2.436971	1314
TC0300002	Rapgef2	NM_00109	"Mus musc	RefSeq	7.76812	9.053007	-2.43663	1315

TC1500001	Dip2b	NM_00115	"Mus musc	RefSeq	6.58672	7.871362	-2.43622	1316
TC0800002	Arhgap10	NM_03011	"Mus musc	RefSeq	8.102784	6.818738	2.43521	1317
TC1200002	Wars	NM_00116	"Mus musc	RefSeq	6.897447	8.180729	-2.43392	1318
TC0600001	Samd9l	NM_01015	"Mus musc	RefSeq	6.325247	7.607596	-2.43235	1319
TC1000000	Fstl3	NM_03138	"Mus musc	RefSeq	7.597317	6.315152	2.432037	1320
TC0700002	Fosb	NM_00803	"Mus musc	RefSeq	10.93843	9.657574	2.429831	1321
TC0500001	Cdk8	NM_15359	"Mus musc	RefSeq	8.015238	6.735016	2.428763	1322
TC0700000	Lgi4	NM_14455	"Mus musc	RefSeq	5.960921	4.680924	2.428385	1323
TC0700001	Trim34a	NM_03068	"Mus musc	RefSeq	5.928802	7.207612	-2.42639	1324
TC1000000	Adora2a	NM_00963	"Mus musc	RefSeq	7.196293	8.474473	-2.42533	1325
TC0500002	Fndc4	NM_02242	"Mus musc	RefSeq	6.415595	5.137578	2.425054	1326
TC0600000	Gng11	NM_02533	"Mus musc	RefSeq	6.528447	7.806021	-2.42431	1327
TC1100001	Camkk1	NM_01888	"Mus musc	RefSeq	6.339598	5.064899	2.419483	1328
TC0400002	Gm12407	XM_00147	"PREDICTEI	RefSeq	6.66445	5.390167	2.418786	1329
TC1300002	Nfil3	NM_01737	"Mus musc	RefSeq	7.251984	5.978446	2.417537	1330
TC0700002	Apoe	NM_00969	"Mus musc	RefSeq	9.221473	7.948366	2.416815	1331
TC1100000	C1qtnf2	NM_02697	"Mus musc	RefSeq	7.447407	6.174836	2.415917	1332
TC0400003	Rps6ka1	NM_00909	"Mus musc	RefSeq	5.549266	6.821544	-2.41543	1333
TC0200005	Samhd1	NM_01885	"Mus musc	RefSeq	7.789358	9.061212	-2.41472	1334
TC0800000	Tbc1d9	NM_00111	"Mus musc	RefSeq	5.537296	6.80909	-2.41462	1335
TC1700002	Rsph9	NM_02933	"Mus musc	RefSeq	5.236346	3.964805	2.414193	1336
TC1600000	Hcls1	NM_00822	"Mus musc	RefSeq	5.544351	6.815632	-2.41376	1337
TC0400001	Sync	NM_02348	"Mus musc	RefSeq	6.966336	5.695961	2.412243	1338
TC1300001	Shc3	BC105645	"Mus musc	GenBank	5.972814	4.702817	2.411611	1339
TC0900001	Col5a3	NM_01691	"Mus musc	RefSeq	7.277997	6.008014	2.411587	1340
TC1700000	Adamts10	NM_17261	"Mus musc	RefSeq	8.768578	7.498612	2.411559	1341
TC0600000	Tes	NM_20717	"Mus musc	RefSeq	4.63594	5.903529	-2.40759	1342
TC0600001	Golt1b	NM_02587	"Mus musc	RefSeq	7.115557	5.849432	2.405147	1343
TC0X00002	Maged1	NM_01979	"Mus musc	RefSeq	8.165205	6.89982	2.403914	1344
TC0300003	Ptgfr	NM_00896	"Mus musc	RefSeq	5.533327	4.268311	2.403299	1345
TC0200004	Dgkz	NM_13830	"Mus musc	RefSeq	6.346216	7.608784	-2.39922	1346
TC1000000	Gadd45b	NM_00865	"Mus musc	RefSeq	10.39201	9.130435	2.397567	1347
TC1700000	Abcg1	NM_00959	"Mus musc	RefSeq	5.972426	7.233227	-2.39629	1348
TC0600001	Tspan12	NM_17300	"Mus musc	RefSeq	8.096897	9.356417	-2.39416	1349
TC0100000	Gm9947	ENSMUST0	cdna:know	ENSEMBL	5.775096	4.516186	2.393149	1350
TC0400000	Musk	NM_00116	"Mus musc	RefSeq	5.113082	3.854621	2.392404	1351
TC0300000	Skil	NM_00127	"Mus musc	RefSeq	8.937152	7.679591	2.390912	1352
TC1100000	8-Sep	NM_03314	"Mus musc	RefSeq	9.262613	8.006674	2.388225	1353
TC1800001	B4galt6	NM_01973	"Mus musc	RefSeq	5.925739	4.671356	2.385651	1354
TC1300002	Parp8	NM_00108	"Mus musc	RefSeq	7.32057	6.06634	2.385398	1355
TC0500003	Trpv4	NM_02201	"Mus musc	RefSeq	5.596396	6.850122	-2.38457	1356
TC1100000	Xaf1	NM_00103	"Mus musc	RefSeq	6.877385	8.12749	-2.37859	1357
TC0600000	Tmem140	NM_19798	"Mus musc	RefSeq	6.932846	8.180504	-2.37456	1358
TC0400000	Rgp1	NM_17286	"Mus musc	RefSeq	6.746569	7.991957	-2.37082	1359
TC1500001	Tspyl5	NM_00108	"Mus musc	RefSeq	5.706477	4.461161	2.370705	1360
TC0300002	Ccdc109b	NM_02577	"Mus musc	RefSeq	6.811631	5.566874	2.369786	1361
TC0400000	Tmem64	NM_18140	"Mus musc	RefSeq	9.413402	8.169379	2.368581	1362

TC0700004	Igsf6	NM_03069	"Mus musc	RefSeq	3.788611	5.031582	-2.36685	1363
TC1700002	Cdc42ep3	NM_02651	"Mus musc	RefSeq	7.072666	8.314095	-2.36433	1364
TC190000C	Fas	NM_00114	"Mus musc	RefSeq	8.056128	9.297508	-2.36425	1365
TC0400004	Agrn	NM_02160	"Mus musc	RefSeq	7.080744	8.322124	-2.36425	1366
TC0800002	Pik3r2	NM_00884	"Mus musc	RefSeq	7.197357	5.956282	2.363746	1367
TC0800001	Nod2	NM_14585	"Mus musc	RefSeq	5.20995	6.450907	-2.36355	1368
TC0500003	Git2	NM_00107	"Mus musc	RefSeq	7.995013	9.235535	-2.36284	1369
TC0200005	Cables2	NM_14585	"Mus musc	RefSeq	6.853976	8.094033	-2.36208	1370
TC0X00000	Prickle3	NM_17509	"Mus musc	RefSeq	5.551685	6.79126	-2.36129	1371
TC130000C	Sfxn1	NM_02732	"Mus musc	RefSeq	7.609111	6.369682	2.361051	1372
TC1400001	Gm8356	ENSMUST0	cdna:putat	ENSEMBL	2.994242	4.232598	-2.3593	1373
TC4_GL456	Gm13298	NM_00108	"Mus musc	RefSeq	5.143829	3.90607	2.358319	1374
TC1600001	Fgd4	NM_13923	"Mus musc	RefSeq	4.991451	3.75577	2.354925	1375
TC1300002	4930486L2	NM_17809	"Mus musc	RefSeq	4.13889	5.374385	-2.35462	1376
TC160000C	Cebpd	NM_00767	"Mus musc	RefSeq	8.674793	7.4402	2.35315	1377
TC0X00000	Xpnpep2	NM_13321	"Mus musc	RefSeq	5.000725	3.767183	2.351436	1378
TC190000C	Ppapdc2	NM_02892	"Mus musc	RefSeq	7.201284	8.433776	-2.34973	1379
TC0500001	Hip1r	NM_14507	"Mus musc	RefSeq	6.274159	7.506544	-2.34955	1380
TC1100003	Atp1b2	NM_01341	"Mus musc	RefSeq	5.303436	6.535366	-2.34881	1381
TC070000C	Mamstr	NM_17241	"Mus musc	RefSeq	4.942075	6.17305	-2.34726	1382
TC0300001	Pde5a	NM_15342	"Mus musc	RefSeq	6.920806	5.689862	2.347205	1383
TC1100002	Zfp354c	NM_01392	"Mus musc	RefSeq	6.011364	4.780456	2.347147	1384
TC160000C	Mkl2	NM_00112	"Mus musc	RefSeq	7.348723	8.579053	-2.34621	1385
TC0600001	Tmem43	NM_02876	"Mus musc	RefSeq	9.08627	7.856486	2.345319	1386
TC4_JH584	Ccl27a	ENSMUST0	cdna:know	ENSEMBL	5.605183	4.376069	2.34423	1387
TC0100003	C130074G1	NM_17869	"Mus musc	RefSeq	6.136804	7.362029	-2.33792	1388
TC0700003	Tjp1	NM_00116	"Mus musc	RefSeq	9.085101	10.30935	-2.33633	1389
TC1800001	Smim3	NM_13413	"Mus musc	RefSeq	4.245658	3.022013	2.33536	1390
TC0200001	Ypel4	NM_00100	"Mus musc	RefSeq	5.783365	4.560949	2.333371	1391
TC090000C	Siae	NM_01173	"Mus musc	RefSeq	7.510366	6.28802	2.333258	1392
TC010000C	Efhc1	NM_02797	"Mus musc	RefSeq	5.577577	4.355259	2.333213	1393
TC0400001	Srm	NM_00927	"Mus musc	RefSeq	7.769358	6.548517	2.330826	1394
TC0200004	Bcl2l1	NM_00128	"BCL2-like :	RefSeq	6.964299	8.185114	-2.33078	1395
TC0600003	Klra3	NM_01064	"Mus musc	RefSeq	2.924376	4.145166	-2.33074	1396
TC170000C	Tnfrsf21	NM_17858	"Mus musc	RefSeq	6.212345	7.43164	-2.32833	1397
TC0800001	Nrp1	NM_00873	"Mus musc	RefSeq	8.748312	9.967377	-2.32796	1398
TC010000C	Prex2	NM_02952	"Mus musc	RefSeq	8.662829	9.879662	-2.32436	1399
TC0300002	S100a1	NM_01130	"Mus musc	RefSeq	6.770702	7.985109	-2.32045	1400
TC100000C	Ptprk	NM_00898	"Mus musc	RefSeq	6.251615	7.464157	-2.31746	1401
TC1000001	Samd5	NM_17727	"Mus musc	RefSeq	6.422439	7.634311	-2.31638	1402
TC0900002	Tmed3	NM_02536	"Mus musc	RefSeq	8.078104	6.866989	2.315165	1403
TC1600001	Rogdi	NM_13318	"Mus musc	RefSeq	7.751616	6.541962	2.312822	1404
TC1700001	Tmem178	NM_02651	"Mus musc	RefSeq	4.613744	5.821186	-2.30928	1405
TC0300001	Dram2	NM_00128	"DNA-dama	RefSeq	7.295412	8.502377	-2.30852	1406
TC0400001	Gm13242	NM_00110	"Mus musc	RefSeq	5.007406	6.214079	-2.30805	1407
TC180000C	Malt1	NM_17283	"Mus musc	RefSeq	5.374588	6.580519	-2.30686	1408
TC1300002	Itga2	NM_00839	"Mus musc	RefSeq	5.808413	4.603058	2.30594	1409

TC0400001	Nppa	NM_00872	"Mus musc	RefSeq	7.092792	8.297563	-2.30501	1410
TC0200002	Snai1	NM_01142	"Mus musc	RefSeq	7.970755	6.76746	2.30265	1411
TC0800000	Arhgef7	NM_00111	"Mus musc	RefSeq	6.885703	8.088003	-2.30106	1412
TC0200001	Slc24a5	NM_17503	"Mus musc	RefSeq	4.915307	3.713213	2.300734	1413
TC1100001	Tmc8	NM_00119	"Mus musc	RefSeq	6.077552	7.279358	-2.30027	1414
TC1400001	Lcp1	NM_00124	"Mus musc	RefSeq	4.470256	5.671695	-2.29969	1415
TC0700004	Osbpl5	NM_00119	"Mus musc	RefSeq	7.537524	6.336798	2.298553	1416
TC0400002	Tmem245	NM_17551	"Mus musc	RefSeq	6.685148	7.885631	-2.29817	1417
TC0100003	Hlx	NM_00825	"Mus musc	RefSeq	7.244944	8.445283	-2.29794	1418
TC1200002	Fbln5	NM_01181	"Mus musc	RefSeq	9.914247	8.714026	2.297749	1419
TC0400002	Plekhg5	NM_00100	"Mus musc	RefSeq	6.890876	8.090922	-2.29747	1420
TC0900002	Pgm3	NM_02835	"Mus musc	RefSeq	6.273497	5.073763	2.296973	1421
TC0500000	Emilin1	NM_13391	"Mus musc	RefSeq	7.115514	5.916496	2.295833	1422
TC1700001	Clip4	NM_17537	"Mus musc	RefSeq	5.877274	4.678371	2.29565	1423
TC0400004	LOC100861	XM_00368	"PREDICTEI	MGI/Jackso	4.599165	3.400623	2.295076	1424
TC0800001	Cx3cl1	NM_00914	"Mus musc	RefSeq	6.067828	7.266289	-2.29495	1425
TC0200002	Fam217b	NM_00108	"Mus musc	RefSeq	5.034005	6.231612	-2.29359	1426
TC1600000	Fam43a	NM_17763	"Mus musc	RefSeq	6.41475	7.611995	-2.29301	1427
TC0X00002	Frmd7	NM_00119	"Mus musc	RefSeq	4.258809	3.061643	2.292888	1428
TC0200004	Actc1	NM_00960	"Mus musc	RefSeq	7.866118	9.062849	-2.2922	1429
TC0500003	Lnx2	NM_08079	"Mus musc	RefSeq	7.856975	9.053331	-2.2916	1430
TC0100000	Gal3st2	NM_19936	"Mus musc	RefSeq	6.306142	5.109917	2.291393	1431
TC0500003	Clip2	NM_00999	"Mus musc	RefSeq	8.362016	7.165924	2.291182	1432
TC1600000	Lsamp	NM_17554	"Mus musc	RefSeq	5.307939	4.112145	2.290709	1433
TC0X00000	Xiap	NM_00968	"Mus musc	RefSeq	6.209655	7.405411	-2.29065	1434
TC1600001	Phldb2	NM_00125	"Mus musc	RefSeq	6.025744	7.221499	-2.29065	1435
TC1100003	Dusp3	NM_02820	"Mus musc	RefSeq	8.680418	9.876037	-2.29043	1436
TC1900000	Fads1	NM_14609	"Mus musc	RefSeq	8.697194	7.502481	2.288993	1437
TC0200002	Sstr4	NM_00921	"Mus musc	RefSeq	5.375456	6.570037	-2.28878	1438
TC1600001	Nfkbiz	NM_00115	"Mus musc	RefSeq	9.324924	8.131687	2.286652	1439
TC1800001	Zfp521	NM_14549	"Mus musc	RefSeq	7.719044	6.526894	2.28493	1440
TC0800001	Zfpm1	NM_00956	"Mus musc	RefSeq	6.422899	7.613419	-2.28235	1441
TC1000001	Dusp6	NM_02626	"Mus musc	RefSeq	8.043615	9.234014	-2.28216	1442
TC0800000	Ddx60	NM_00108	"Mus musc	RefSeq	4.800434	5.989964	-2.28078	1443
TC1000001	Csrp2	NM_00779	"Mus musc	RefSeq	8.581775	7.392893	2.27976	1444
TC1300002	Rgs7bp	NM_02987	"Mus musc	RefSeq	3.738624	4.92715	-2.2792	1445
TC1200002	Rps6kl1	NM_14624	"Mus musc	RefSeq	5.521488	6.709129	-2.2778	1446
TC1100002	Stc2	NM_01149	"Mus musc	RefSeq	5.668674	4.481357	2.277288	1447
TC0100003	Nenf	NM_02542	"Mus musc	RefSeq	7.648054	6.460771	2.277235	1448
TC0600001	Cand2	NM_02595	"Mus musc	RefSeq	6.130764	4.944063	2.276316	1449
TC0600000	Prdm5	NM_02754	"Mus musc	RefSeq	6.790976	5.604559	2.275868	1450
TC0100000	Aox3	NM_02361	"Mus musc	RefSeq	4.621847	3.43581	2.275269	1451
TC0900001	Elovl5	NM_13425	"Mus musc	RefSeq	8.945828	10.13184	-2.27522	1452
TC0500002	Sorcs2	NM_03088	"Mus musc	RefSeq	6.097905	4.912236	2.274689	1453
TC1_GL456	AC168977.	ENSMUST0	cdna:novel	ENSEMBL	6.573976	7.758988	-2.27365	1454
TC0400000	Slc44a1	NM_00115	"Mus musc	RefSeq	8.988366	7.804139	2.272416	1455
TC1300002	Nkd2	NM_02818	"Mus musc	RefSeq	6.356297	5.17249	2.271755	1456

TC0300000	S100a4	NM_01131	"Mus musc	RefSeq	4.890469	3.70714	2.271002	1457
TC1700001	Itfg3	NM_00120	"Mus musc	RefSeq	7.661242	6.478537	2.27002	1458
TC0600001	Sgce	NM_00113	"Mus musc	RefSeq	7.607545	6.425487	2.269002	1459
TC0600001	Bhlhe40	NM_01149	"Mus musc	RefSeq	9.782181	8.600747	2.268021	1460
TC0200005	Pltp	NM_01112	"Mus musc	RefSeq	8.968475	10.14934	-2.26713	1461
TC0900001	Cish	NM_00989	"Mus musc	RefSeq	5.710273	6.891112	-2.26709	1462
TC0600003	Kcna5	NM_14598	"Mus musc	RefSeq	8.24204	9.422665	-2.26675	1463
TC0200002	Plcg1	NM_02128	"Mus musc	RefSeq	7.792182	8.972701	-2.26658	1464
TC0300003	Fgf2	NM_00800	"Mus musc	RefSeq	7.024185	5.843978	2.266093	1465
TC1200001	Gm11042	ENSMUST0	cdna:know	ENSEMBL	8.59415	9.772411	-2.26304	1466
TC1300001	Arsb	NM_00971	"Mus musc	RefSeq	7.918929	6.741445	2.26182	1467
TC0600000	Mest	NM_00125	"Mus musc	RefSeq	6.519059	7.695949	-2.26089	1468
TC1300001	Ccdc125	NM_00116	"Mus musc	RefSeq	3.932861	5.109542	-2.26056	1469
TC0400003	Grrp1	NM_00109	"Mus musc	RefSeq	5.683291	6.859803	-2.2603	1470
TC0400001	Asap3	NM_00100	"Mus musc	RefSeq	6.600296	5.424323	2.259452	1471
TC0700000	Prkcg	NM_01110	"Mus musc	RefSeq	6.355897	7.530499	-2.25731	1472
TC1_GL456	AC133103.	ENSMUST0	cdna:know	ENSEMBL	7.857897	9.032456	-2.25724	1473
TC0200001	Dusp2	NM_01009	"Mus musc	RefSeq	5.206481	6.380996	-2.25717	1474
TC1200002	Ccdc88c	NM_02668	"Mus musc	RefSeq	6.370117	7.54403	-2.25623	1475
TC0X00001	Mum1l1	NM_17554	"Mus musc	RefSeq	5.115633	3.942209	2.255464	1476
TC1200000	Hpcal1	NM_01667	"Mus musc	RefSeq	7.789056	8.962287	-2.25516	1477
TC1700001	Tmem204	NM_00100	"Mus musc	RefSeq	8.644845	9.816621	-2.25289	1478
TC1400000	Gm3115	XM_00147	"PREDICTEI	RefSeq	3.722682	4.893548	-2.25147	1479
TC1100002	Meis1	NM_01078	"Mus musc	RefSeq	6.881946	5.711111	2.25142	1480
TC1600001	Slc15a2	NM_02130	"Mus musc	RefSeq	4.608465	5.779023	-2.25099	1481
TC0500002	Lcorl	NM_17814	"Mus musc	RefSeq	4.895682	6.065652	-2.25007	1482
TC1300000	Glrx	NM_05310	"Mus musc	RefSeq	6.218147	5.048378	2.249757	1483
TC1400001	Gm3476	XM_00147	"PREDICTEI	RefSeq	3.487108	4.65644	-2.24908	1484
TC0X00002	Tspan6	NM_01965	"Mus musc	RefSeq	6.890209	8.059238	-2.2486	1485
TC1100000	Lif	NM_00850	"Mus musc	RefSeq	5.847987	4.680534	2.246148	1486
TC1400002	Slc25a30	NM_02623	"Mus musc	RefSeq	6.452289	7.618468	-2.24417	1487
TC0200005	B4galt5	NM_01983	"Mus musc	RefSeq	7.802956	6.637499	2.243043	1488
TC0700000	Izumo1	NM_00101	"Mus musc	RefSeq	5.864618	7.029793	-2.2426	1489
TC1200002	Wdr60	NM_14603	"Mus musc	RefSeq	5.332755	4.16806	2.241858	1490
TC0600001	Il17ra	NM_00835	"Mus musc	RefSeq	6.14787	4.983347	2.241591	1491
TC0100003	Sertad4	NM_00117	"Mus musc	RefSeq	6.361762	5.198421	2.239755	1492
TC0200000	Pkn3	NM_15380	"Mus musc	RefSeq	5.981704	7.14335	-2.23713	1493
TC0600000	Trim24	NM_14507	"Mus musc	RefSeq	7.118287	8.279476	-2.23642	1494
TC0300000	Kcnn3	NM_08046	"Mus musc	RefSeq	5.934191	4.773115	2.236242	1495
TC1500001	Ly6c1	NM_00125	"Mus musc	RefSeq	9.555896	10.71656	-2.2356	1496
TC0100001	Fmod	NM_02135	"Mus musc	RefSeq	5.702973	4.542805	2.234835	1497
TC0100001	Tnnt2	NM_00113	"Mus musc	RefSeq	8.02472	9.184774	-2.23466	1498
TC0300000	Tnik	NM_00116	"Mus musc	RefSeq	5.282575	4.123033	2.233865	1499
TC0200000	Ncs1	NM_01968	"Mus musc	RefSeq	5.75269	4.593149	2.233863	1500
TC0400000	Hrct1	NM_02751	"Mus musc	RefSeq	5.35191	6.511236	-2.23353	1501
TC1100001	Slfn5	NM_18320	"Mus musc	RefSeq	7.73274	8.891819	-2.23315	1502
TC1700000	Pkd1	NM_01363	"Mus musc	RefSeq	9.217723	8.059082	2.23247	1503

TC0800001	Slc7a6	NM_17879	"Mus musc	RefSeq	6.8883	5.729997	2.231947	1504
TC1600001	Ripply3	NM_13322	"Mus musc	RefSeq	6.379127	7.53664	-2.23073	1505
TC010000C	Gm7609	NM_00108	"Mus musc	RefSeq	5.573126	6.729508	-2.22898	1506
TC150000C	Lifr	NM_00111	"Mus musc	RefSeq	7.353636	8.509974	-2.22891	1507
TC0300002	Npr1	NM_00872	"Mus musc	RefSeq	7.443117	8.599374	-2.22878	1508
TC1800001	Cdo1	NM_03303	"Mus musc	RefSeq	6.195483	5.039861	2.227804	1509
TC0300002	Adam15	NM_00961	"Mus musc	RefSeq	7.688684	8.844066	-2.22743	1510
TSUnmapp	Egr3	NM_00128	"early grow	RefSeq	7.869931	6.715191	2.226442	1511
TC0600002	Atoh8	NM_15377	"Mus musc	RefSeq	6.706395	7.860481	-2.22543	1512
TC1100002	N4bp3	NM_14597	"Mus musc	RefSeq	6.175387	7.329399	-2.22532	1513
TC0Y00000	Gm21742	ENSMUST0	cdna:novel	ENSEMBL	6.712704	5.560092	2.22316	1514
TC0100002	Fam124b	NM_17342	"Mus musc	RefSeq	4.62269	5.774182	-2.22144	1515
TC0900001	Plscr1	NM_01163	"Mus musc	RefSeq	5.747345	6.898801	-2.22138	1516
TC120000C	Syne2	NM_00100	"Mus musc	RefSeq	7.6206	8.7713	-2.22022	1517
TC0400002	Bnc2	NM_17287	"Mus musc	RefSeq	5.492606	4.343922	2.217116	1518
TC0500002	Gbp10	NM_00103	"Mus musc	RefSeq	8.09579	9.243785	-2.21606	1519
TC180000C	Isoc1	NM_02547	"Mus musc	RefSeq	8.010553	6.862993	2.215389	1520
TC0400002	Gm21104	XM_00368	"PREDICTEI	RefSeq	6.551867	5.404628	2.214896	1521
TC1100003	Usp43	NM_17375	"Mus musc	RefSeq	4.869444	6.016554	-2.2147	1522
TC010000C	Nck2	NM_01087	"Mus musc	RefSeq	6.213351	7.360272	-2.21441	1523
TC020000C	Mrrf	NM_02642	"Mus musc	RefSeq	6.685396	7.83213	-2.21412	1524
TC1400002	Gm10338	XM_00147	"PREDICTEI	RefSeq	4.277037	5.423387	-2.21353	1525
TC0600003	Gimap1	NM_17586	"Mus musc	RefSeq	6.084218	7.229131	-2.21133	1526
TC0400002	Mup1	NM_03118	"Mus musc	RefSeq	4.545643	3.401546	2.210078	1527
TC0X00002	Cxx1b	NM_00101	"Mus musc	RefSeq	8.662363	9.804616	-2.20726	1528
TC0600002	Creb3l2	NM_17866	"Mus musc	RefSeq	8.780525	7.638365	2.207112	1529
TC030000C	D3Ertd254(	NM_00110	"Mus musc	RefSeq	6.457323	5.315452	2.20667	1530
TC0200002	Eya2	NM_00127	"Mus musc	RefSeq	5.515134	4.373318	2.206586	1531
TC100000C	Ahi1	NM_02620	"Mus musc	RefSeq	5.644892	4.50446	2.20447	1532
TC0800002	Klhl2	NM_17863	"Mus musc	RefSeq	5.755192	6.895004	-2.20352	1533
TC1200002	Ttc7b	NM_00103	"Mus musc	RefSeq	7.496512	8.635817	-2.20275	1534
TC0700002	Zfp715	NM_02726	"Mus musc	RefSeq	6.282503	7.421207	-2.20183	1535
TC0300001	Naaladl2	XM_97522	"PREDICTEI	RefSeq	4.321964	3.18338	2.201648	1536
TC030000C	Tbl1xr1	NM_03073	"Mus musc	RefSeq	7.020445	5.881872	2.201631	1537
TC1400001	Gm6676	XM_89104	"PREDICTEI	RefSeq	3.659801	4.796979	-2.1995	1538
TC1400001	Gm16434	XM_00147	"PREDICTEI	RefSeq	3.659801	4.796979	-2.1995	1538
TC1500002	Rnd1	NM_17261	"Mus musc	RefSeq	5.204126	6.341184	-2.19932	1540
TC0400003	Phactr4	NM_17530	"Mus musc	RefSeq	7.074549	8.210757	-2.19803	1541
TC0300003	Pmp2	NM_00103	"Mus musc	RefSeq	4.142549	5.278655	-2.19787	1542
TC060000C	Smo	NM_17699	"Mus musc	RefSeq	8.365202	7.229233	2.197661	1543
TC0600003	Herc6	NM_02599	"Mus musc	RefSeq	6.000573	7.135513	-2.19609	1544
TC0900002	Pccb	NM_02583	"Mus musc	RefSeq	6.907513	5.772968	2.195493	1545
TC140000C	4930555G(	NM_17539	"Mus musc	RefSeq	3.286694	4.420897	-2.19497	1546
TC080000C	Hpgd	NM_00827	"Mus musc	RefSeq	5.579693	4.445615	2.194783	1547
TC0300001	Casq2	NM_00981	"Mus musc	RefSeq	4.842378	5.976335	-2.1946	1548
TC080000C	Acsl1	NM_00798	"Mus musc	RefSeq	6.1842	7.31776	-2.194	1549
TC180000C	Greb1l	NM_00108	"Mus musc	RefSeq	5.116733	3.983175	2.193992	1550

TC1100003	Meox1	NM_01079	"Mus musc	RefSeq	9.170796	8.037945	2.192917	1551
TC0600001	Tom1	ENSMUST0	cdna:know	ENSEMBL	8.361251	7.229287	2.191569	1552
TC1800001	Mcc	NM_00108	"Mus musc	RefSeq	8.205627	9.337268	-2.19108	1553
TC130000C	Cmah	NM_00111	"Mus musc	RefSeq	4.994335	3.862958	2.190677	1554
TC0600002	Plxna4	NM_17575	"Mus musc	RefSeq	6.077346	7.207878	-2.1894	1555
TC0300001	Dennd2c	NM_17785	"Mus musc	RefSeq	4.481266	5.611744	-2.18931	1556
TC110000C	Ramp3	NM_01951	"Mus musc	RefSeq	5.982512	7.112273	-2.18823	1557
TC0900002	Gm684	NM_00119	"Mus musc	RefSeq	5.613452	4.483953	2.187828	1558
TC060000C	Mturn	NM_00128	"maturin, r	RefSeq	7.804153	6.674714	2.187737	1559
TC0200001	Olfr1034	NM_00101	"Mus musc	RefSeq	3.796163	2.66723	2.186969	1560
TC1700001	Prkce	NM_01110	"Mus musc	RefSeq	5.9588	7.086988	-2.18584	1561
TC090000C	Myo1e	NM_18107	"Mus musc	RefSeq	7.484465	8.61265	-2.18584	1562
TC1100001	Myo18a	NM_01158	"Mus musc	RefSeq	6.439057	7.566993	-2.18546	1563
TC1500001	Ptk2	NM_00798	"Mus musc	RefSeq	7.547124	8.6748	-2.18507	1564
TC0500001	Acacb	NM_13390	"Mus musc	RefSeq	6.424433	7.550462	-2.18257	1565
TC1400001	Gm3460	XM_00147	"PREDICTEI	RefSeq	4.214041	5.339897	-2.18231	1566
TC0400001	Hspb7	NM_01386	"Mus musc	RefSeq	6.630716	7.756101	-2.1816	1567
TC0500003	Trip6	NM_01163	"Mus musc	RefSeq	6.900574	5.7771	2.17871	1568
TC1900001	Dagla	NM_19811	"Mus musc	RefSeq	6.945128	5.821954	2.178257	1569
TC100000C	Ggt5	NM_01182	"Mus musc	RefSeq	6.478213	5.355583	2.177436	1570
TC170000C	Pim1	BC055316	"Mus musc	GenBank	8.87588	7.753301	2.177359	1571
TC1400001	Usp54	NM_03018	"Mus musc	RefSeq	6.946322	5.824994	2.175471	1572
TC0200005	Eng	NM_00114	"Mus musc	RefSeq	10.48322	11.60357	-2.174	1573
TC0600002	Rbpsuh-rs3	ENSMUST0	cdna:know	ENSEMBL	9.261059	8.140891	2.173723	1574
TC1900001	Tjp2	NM_00119	"Mus musc	RefSeq	6.90738	8.027505	-2.17366	1575
TC050000C	Abcb4	NM_00883	"Mus musc	RefSeq	4.168078	5.287683	-2.17288	1576
TC0700001	Olfr558	NM_14709	"Mus musc	RefSeq	3.881388	4.998491	-2.16911	1577
TC1200001	Evl	NM_00116	"Mus musc	RefSeq	6.537953	7.654998	-2.16902	1578
TC0400002	Lpar1	NM_17298	"Mus musc	RefSeq	6.794655	5.67894	2.167024	1579
TC0400002	Gm13305	NM_00109	"Mus musc	RefSeq	7.318718	6.203215	2.166705	1581
TC4_JH584	LOC100861	ENSMUST0	cdna:know	ENSEMBL	7.318718	6.203215	2.166705	1581
TC4_JH584	LOC100861	ENSMUST0	cdna:know	ENSEMBL	7.318718	6.203215	2.166705	1581
TC0700004	Numbl	NM_01095	"Mus musc	RefSeq	7.659587	6.544135	2.166629	1583
TC190000C	C030046E1	NM_00108	"Mus musc	RefSeq	7.089883	8.204711	-2.16569	1584
TC190000C	Pygm	NM_01122	"Mus musc	RefSeq	7.052415	8.167199	-2.16563	1585
TC0100003	Hsd17b7	NM_01047	"Mus musc	RefSeq	6.349437	5.234741	2.165494	1586
TC0900001	S1pr2	NM_01033	"Mus musc	RefSeq	7.476228	6.362353	2.164262	1587
TC1100002	Tns3	NM_00108	"Mus musc	RefSeq	6.939075	5.82621	2.162747	1588
TC1100002	Slc1a4	NM_01886	"Mus musc	RefSeq	5.733804	4.62113	2.162461	1589
TC1500001	Arfgap3	NM_02544	"Mus musc	RefSeq	8.35957	7.247093	2.162166	1590
TC0X00001	Magee1	NM_05320	"Mus musc	RefSeq	5.858925	4.746587	2.161957	1591
TC0900001	Dusp7	NM_15345	"Mus musc	RefSeq	7.713251	8.824221	-2.15991	1592
TC0X0000C	Nhsl2	NM_00116	"Mus musc	RefSeq	5.45873	6.569554	-2.15969	1593
TC0200001	Cd59a	NM_00765	"Mus musc	RefSeq	7.339997	8.449287	-2.15739	1594
TC0800002	Exoc3l	NM_17778	"Mus musc	RefSeq	6.295562	7.404762	-2.15726	1595
TC0100001	Creg1	NM_01180	"Mus musc	RefSeq	8.263545	7.155209	2.155968	1596
TC0200004	Creb3l1	NM_01195	"Mus musc	RefSeq	8.669718	7.561583	2.155668	1597

TC0100003	Srgap2	NM_00108	"Mus musc	RefSeq	7.644451	8.752531	-2.15559	1598
TC0900001	Ctsh	NM_00780	"Mus musc	RefSeq	6.268734	5.160779	2.155399	1599
TC0100003	Darc	NM_01004	"Mus musc	RefSeq	4.810792	3.70327	2.154752	1600
TC0200003	Ptges	NM_02241	"Mus musc	RefSeq	5.969738	4.862373	2.154518	1601
TC1000001	Ppfia2	NM_00120	"Mus musc	RefSeq	4.434027	3.327388	2.153434	1602
TC0700001	Wnt11	NM_00128	"wingless-r	RefSeq	6.132041	5.02575	2.152914	1603
TC0700001	Inpp5a	NM_00112	"Mus musc	RefSeq	7.94751	9.052713	-2.15129	1604
TC0700000	Ccdc8	NM_00110	"Mus musc	RefSeq	5.347238	4.242367	2.150796	1605
TC1100001	Tmigd1	NM_02565	"Mus musc	RefSeq	3.625297	4.729214	-2.14938	1606
TC0500000	Evc2	NM_14592	"Mus musc	RefSeq	6.594956	5.491386	2.148858	1607
TC0700000	Gng8	NM_01032	"Mus musc	RefSeq	5.785114	4.68289	2.146854	1608
TC0X00000	Zfp449	NM_03013	"Mus musc	RefSeq	7.16599	6.064039	2.146448	1609
TC0400002	Tbc1d2	NM_19866	"Mus musc	RefSeq	5.474089	6.575697	-2.14594	1610
TC0X00003	Car5b	NM_18131	"Mus musc	RefSeq	5.348974	4.247455	2.145805	1611
TC1800001	Stard4	NM_13377	"Mus musc	RefSeq	5.1466	6.246384	-2.14323	1612
TC1000002	Shc2	NM_00102	"Mus musc	RefSeq	6.509821	5.41004	2.143222	1613
TC1400001	Gm6356	ENSMUST0	cdna:putat	ENSEMBL	3.356132	4.455886	-2.14318	1614
TC0300002	Tm4sf1	NM_00853	"Mus musc	RefSeq	8.268605	9.368345	-2.14316	1615
TC1300002	Fst	NM_00804	"Mus musc	RefSeq	5.451591	4.3533	2.141009	1616
TC1400001	Gm3727	ENSMUST0	cdna:putat	ENSEMBL	4.07743	5.175554	-2.14076	1617
TC0800002	Kifc3	NM_00114	"Mus musc	RefSeq	7.03868	8.135958	-2.13951	1618
TC1200000	Akap6	NM_19811	"Mus musc	RefSeq	4.035256	5.132463	-2.1394	1619
TC0200002	Cebpb	NM_00988	"Mus musc	RefSeq	7.977111	6.880769	2.138119	1620
TC0500003	Hip1	NM_14600	"Mus musc	RefSeq	7.657779	8.753394	-2.13704	1621
TC0900003	Abhd14a	NM_00111	"Mus musc	RefSeq	6.567952	5.472418	2.136922	1622
TC0700001	Tmem159	NM_14558	"Mus musc	RefSeq	8.22273	7.12741	2.136605	1623
TC0300002	Gstm4	NM_02676	"Mus musc	RefSeq	6.655886	5.561063	2.135869	1624
TC1500001	Mettl7a1	NM_02733	"Mus musc	RefSeq	7.093596	5.998996	2.135539	1625
TC1200001	Ncoa1	NM_01088	"Mus musc	RefSeq	8.477384	7.383048	2.135148	1626
TC0100003	Lbr	NM_13381	"Mus musc	RefSeq	8.384622	9.478333	-2.13422	1627
TC1400001	Gm3594	XM_00147	"PREDICTEI	RefSeq	3.26926	4.362074	-2.1329	1628
TC0100000	Col4a3	NM_00773	"Mus musc	RefSeq	6.334559	5.243029	2.130999	1629
TC1500001	Ghr	NM_00104	"Mus musc	RefSeq	7.047577	5.956425	2.130441	1630
TC0X00003	Capn6	NM_00760	"Mus musc	RefSeq	4.360125	3.269107	2.130243	1631
TC0500001	Slc24a6	NM_13322	"Mus musc	RefSeq	8.342138	7.251171	2.130168	1632
TC0X00002	Atp11c	NM_00103	"Mus musc	RefSeq	6.435974	7.525422	-2.12793	1633
TC1100001	Git1	NM_00100	"Mus musc	RefSeq	8.010554	9.097749	-2.12461	1634
TC0600002	Cyp26b1	NM_17547	"Mus musc	RefSeq	5.914766	7.00083	-2.12294	1635
TC0200000	Pter	NM_00896	"Mus musc	RefSeq	6.21571	5.129677	2.122895	1636
TC0600002	Tril	NM_02581	"Mus musc	RefSeq	7.902575	6.816602	2.122807	1637
TC0600002	4921507PC	NM_02756	"Mus musc	RefSeq	3.784652	4.870101	-2.12204	1638
TC0100003	Chpf	NM_00100	"Mus musc	RefSeq	7.074135	5.988832	2.121821	1639
TC1000000	Slc16a9	NM_02580	"Mus musc	RefSeq	5.975616	4.891322	2.120338	1640
TC0100002	Inhbb	NM_00838	"Mus musc	RefSeq	7.153786	6.070048	2.119521	1641
TC1000001	E2f7	NM_17860	"Mus musc	RefSeq	5.188452	6.272128	-2.11943	1642
TC0200000	Usp6nl	NM_00108	"Mus musc	RefSeq	6.380011	7.46343	-2.11905	1643
TC0600003	Clec12b	NM_02770	"Mus musc	RefSeq	3.855669	4.939029	-2.11897	1644

TC0600001	Efcc1	NM_00115	"Mus musc	RefSeq	6.35146	5.269958	2.116238	1645
TC0400001	Tal1	NM_01152	"Mus musc	RefSeq	5.439274	6.520126	-2.11529	1646
TC0300002	Golim4	NM_17519	"Mus musc	RefSeq	8.167553	7.086799	2.115141	1647
TC0200005	4930402H2	NM_02943	"Mus musc	RefSeq	7.102162	8.182419	-2.11441	1648
TC1100003	Tmem97	NM_13370	"Mus musc	RefSeq	6.854116	5.773943	2.11429	1649
TC0900000	Uaca	NM_02828	"Mus musc	RefSeq	7.713467	8.793513	-2.1141	1650
TC0100003	Nr5a2	NM_00115	"Mus musc	RefSeq	4.309586	5.389395	-2.11376	1651
TC0400002	Fam219a	NM_00115	"Mus musc	RefSeq	6.635143	7.714031	-2.11241	1652
TC0800001	Sntb2	NM_00922	"Mus musc	RefSeq	8.981823	7.903396	2.111732	1653
TC1100003	Tnfaip1	NM_00939	"Mus musc	RefSeq	8.275787	9.352426	-2.10912	1654
TC1700000	Tnfaip8l1	NM_02556	"Mus musc	RefSeq	4.672668	5.748775	-2.10834	1655
TC0100000	Crispld1	NM_03140	"Mus musc	RefSeq	4.925363	6.000238	-2.10654	1656
TC0300000	Prkab2	NM_18299	"Mus musc	RefSeq	6.75207	5.677403	2.106236	1657
TC0500000	Abcb1b	NM_01107	"Mus musc	RefSeq	6.278296	7.35147	-2.10406	1658
TC0100002	Kif1a	NM_00111	"Mus musc	RefSeq	6.119845	5.048279	2.101713	1659
TC1700000	Psmb8	NM_01072	"Mus musc	RefSeq	6.114605	7.185654	-2.10096	1660
TC0800001	Foxc2	NM_01351	"Mus musc	RefSeq	5.560949	4.489923	2.100927	1661
TC1900000	Hectd2	NM_17263	"Mus musc	RefSeq	4.704205	3.634469	2.099049	1662
TC0500002	Adamts3	NM_17787	"Mus musc	RefSeq	4.774342	3.70497	2.09852	1663
TC0100000	Rab23	NM_00899	"Mus musc	RefSeq	6.222197	5.155693	2.094352	1664
TC1000002	Mmp11	NM_00860	"Mus musc	RefSeq	7.089596	6.023277	2.094084	1665
TC1000000	Gja1	NM_01028	"Mus musc	RefSeq	5.241869	6.308059	-2.0939	1666
TC0600003	Sox5	NM_01144	"Mus musc	RefSeq	5.391149	4.32547	2.093155	1667
TC1100002	Rasd1	NM_00902	"Mus musc	RefSeq	6.301461	7.365833	-2.09126	1668
TC1500000	Phf20l1	NM_00108	"Mus musc	RefSeq	6.933031	7.997342	-2.09117	1669
TC0500002	Scarb2	NM_00764	"Mus musc	RefSeq	9.155447	8.091192	2.09109	1670
TC0400002	Gba2	NM_17269	"Mus musc	RefSeq	6.64354	7.707647	-2.09088	1671
TC0900000	Rpp25	NM_13398	"Mus musc	RefSeq	5.012283	3.948497	2.09041	1672
TC1400000	Stmn4	NM_01967	"Mus musc	RefSeq	5.775055	4.711383	2.090245	1673
TC0800001	Nlrc5	NM_00103	"Mus musc	RefSeq	5.165478	6.228042	-2.08864	1674
TC1000001	Ppm1h	NM_17691	"Mus musc	RefSeq	5.426846	6.489225	-2.08837	1675
TC0700003	Trim30d	NM_00116	"Mus musc	RefSeq	5.000353	6.061938	-2.08722	1676
TC0700000	Rhpn2	NM_02789	"Mus musc	RefSeq	5.411726	6.473295	-2.0872	1677
TC0800002	Bst2	NM_19809	"Mus musc	RefSeq	7.748395	8.809635	-2.08672	1678
TC1400000	Arhgef3	NM_00128	"Rho guani	RefSeq	7.0796	8.140525	-2.08627	1679
TC0500003	Sh2b3	NM_00850	"Mus musc	RefSeq	7.084046	8.143286	-2.08383	1680
TC0600000	Calu	NM_00759	"Mus musc	RefSeq	9.60749	8.5494	2.082173	1681
TC0700004	Cdkn1c	NM_00987	"Mus musc	RefSeq	7.620828	8.67862	-2.08174	1682
TC1400001	Gm21103	XM_00368	"PREDICTEI	RefSeq	4.588197	5.645607	-2.08119	1683
TC0300003	Lmo4	NM_00116	"Mus musc	RefSeq	7.063993	6.006856	2.080798	1684
TC0700001	Gab2	NM_00116	"Mus musc	RefSeq	9.366676	8.309628	2.08067	1685
TC0100003	Rgl1	NM_01684	"Mus musc	RefSeq	7.757121	8.814103	-2.08058	1686
TC1900000	Rab3il1	NM_14453	"Mus musc	RefSeq	6.612683	5.555992	2.080155	1687
TC0700003	Crtc3	NM_17386	"Mus musc	RefSeq	8.115427	7.05877	2.080106	1688
TC1500001	St3gal1	NM_00917	"Mus musc	RefSeq	7.06132	8.117292	-2.07912	1689
TC1400000	Ktn1	NM_00847	"Mus musc	RefSeq	6.790671	7.846446	-2.07884	1690
TC1800000	Asxl3	NM_00116	"Mus musc	RefSeq	4.506184	5.561554	-2.07825	1691

TC0900000	Hexa	NM_01042	"Mus musc	RefSeq	8.479597	7.424356	2.078065	1692
TC0700001	Swap70	NM_00930	"Mus musc	RefSeq	6.64426	7.699405	-2.07793	1693
TC1200000	Itsn2	NM_00119	"Mus musc	RefSeq	7.261337	8.316464	-2.0779	1694
TC0500002	Cnot6l	NM_00128	"CCR4-NOT	RefSeq	7.097769	8.152872	-2.07787	1695
TC1400000	Gm3047	XM_00147	"PREDICTE	RefSeq	4.177483	5.23249	-2.07773	1696
TC1700000	Tap1	NM_00116	"Mus musc	RefSeq	6.94652	8.001261	-2.07735	1697
TC1000002	Nr1h4	NM_00116	"Mus musc	RefSeq	4.60807	3.553369	2.077288	1698
TC1000003	Gli1	NM_01029	"Mus musc	RefSeq	6.136025	5.082287	2.075902	1699
TC0100002	Pid1	NM_00100	"Mus musc	RefSeq	5.960426	4.907495	2.074741	1700
TC0700004	Bnip3	NM_00976	"Mus musc	RefSeq	5.976846	7.029181	-2.07388	1701
TC0100003	BC034090	XM_14897	"PREDICTE	RefSeq	6.089226	5.037182	2.073465	1702
TC0200004	Acss1	NM_08057	"Mus musc	RefSeq	5.210782	6.262445	-2.07292	1703
TC0900002	Rab11a	NM_01738	"Mus musc	RefSeq	6.573387	7.624509	-2.07214	1704
TC1900000	Gstp2	NM_18179	"Mus musc	RefSeq	8.436723	7.38579	2.071869	1705
TC1300001	Dtnbp1	NM_02577	"Mus musc	RefSeq	7.774178	6.724443	2.07015	1706
TC1200000	Sypl	NM_01363	"Mus musc	RefSeq	5.894206	6.943797	-2.06994	1707
TC1000002	Gopc	NM_05318	"Mus musc	RefSeq	6.134672	7.184239	-2.06991	1708
TC1200000	Flrt2	NM_20151	"Mus musc	RefSeq	6.555173	5.506181	2.069084	1709
TC1700000	Cyp39a1	NM_00128	"cytochron	RefSeq	6.954629	5.90597	2.068606	1710
TC0500001	Gatsl2	NM_03071	"Mus musc	RefSeq	6.726394	5.679116	2.066627	1711
TC0300001	Extl2	NM_02138	"Mus musc	RefSeq	6.386264	5.339565	2.065798	1712
TC0400001	Stmn1	NM_01964	"Mus musc	RefSeq	5.224829	6.271167	-2.06528	1713
TC1000002	S1pr4	NM_01010	"Mus musc	RefSeq	4.239065	5.285379	-2.06525	1714
TC0200002	Tcf15	NM_00932	"Mus musc	RefSeq	8.587401	9.63304	-2.06428	1715
TC0100003	Ppp2r5a	NM_14488	"Mus musc	RefSeq	8.618479	9.663841	-2.06388	1716
TC1700002	Gm10495	XM_00308	"PREDICTE	MGI/Jackso	7.465958	6.420605	2.063871	1717
TC0200004	Meis2	NM_00115	"Mus musc	RefSeq	5.957646	4.913526	2.062108	1718
TC0700004	Cpxm2	NM_01886	"Mus musc	RefSeq	5.549704	4.506263	2.061138	1719
TC0100003	Shisa4	NM_17525	"Mus musc	RefSeq	5.949758	4.906454	2.060942	1720
TSUnmapp	Ddit3	NM_00129	"DNA-dama	RefSeq	8.493016	7.450043	2.060469	1721
TC0200001	Lgr4	NM_17267	"Mus musc	RefSeq	5.778538	4.736264	2.059471	1722
TC0900000	Mmp27	NM_00103	"Mus musc	RefSeq	4.29938	3.257379	2.059082	1723
TC0700003	Fah	NM_01017	"Mus musc	RefSeq	6.806621	5.764627	2.059072	1724
TC0X00001	Scml2	NM_13319	"Mus musc	RefSeq	4.757511	3.717693	2.055968	1725
TC0X00003	Armcx2	NM_00116	"Mus musc	RefSeq	6.744235	5.70444	2.055935	1726
TC0900002	Polr2m	NM_00116	"Mus musc	RefSeq	6.270968	7.309691	-2.05441	1727
TC0700004	Rnh1	NM_00117	"Mus musc	RefSeq	8.01531	6.977552	2.053035	1728
TC0900002	AW551984	NM_00119	"Mus musc	RefSeq	4.768387	3.731239	2.052167	1729
TC1700001	Tead3	NM_00109	"Mus musc	RefSeq	6.518764	5.482759	2.050542	1730
TC1200000	Cmpk2	NM_02055	"Mus musc	RefSeq	7.305454	8.341057	-2.04997	1731
TC0300002	Synpo2	NM_08045	"Mus musc	RefSeq	5.590651	4.555535	2.049278	1732
TC0200003	Arhgap21	NM_00108	"Mus musc	RefSeq	8.52399	7.488936	2.04919	1733
TC0900002	C2cd2l	NM_02790	"Mus musc	RefSeq	6.526864	7.5619	-2.04917	1734
TC1400001	Gm5458	NM_00102	"Mus musc	RefSeq	4.201929	5.236316	-2.04824	1735
TC1100000	Gm2a	NM_01029	"Mus musc	RefSeq	8.831959	7.797868	2.047823	1736
TC1400002	Psme2	NM_01119	"Mus musc	RefSeq	6.401226	7.434889	-2.04722	1737
TC0100001	Phlpp1	NM_13382	"Mus musc	RefSeq	7.341207	8.374756	-2.04705	1738

TC1000002	Pdxk	NM_17213	"Mus musc	RefSeq	7.074203	6.040719	2.046962	1739
TC0700000	Mtmr10	NM_17274	"Mus musc	RefSeq	7.252339	8.285545	-2.04657	1740
TC1100000	Wnt9a	NM_13929	"Mus musc	RefSeq	7.038352	6.005179	2.04652	1741
TC0500003	Ift81	NM_00987	"Mus musc	RefSeq	5.183239	4.150082	2.046498	1742
TC0100000	Adam23	NM_00117	"Mus musc	RefSeq	4.973405	6.006258	-2.04607	1743
TC0400002	B4galt1	NM_02230	"Mus musc	RefSeq	9.270369	8.237523	2.046057	1744
TC0X00000	Atp6ap2	NM_02743	"Mus musc	RefSeq	8.343122	7.31168	2.044066	1745
TC1000002	Acss3	NM_00114	"Mus musc	RefSeq	4.449963	3.418589	2.04397	1746
TC1600000	Scarf2	NM_15379	"Mus musc	RefSeq	7.400606	6.370385	2.042337	1747
TC0700004	Gga2	NM_02875	"Mus musc	RefSeq	6.230892	5.201538	2.04111	1748
TC0800003	Znrf1	NM_00116	"Mus musc	RefSeq	6.138362	7.167684	-2.04107	1749
TC0400000	Gm20938	XM_00148	"PREDICTEI	RefSeq	4.876326	3.847079	2.040959	1750
TC0400002	Gm12429	NM_00127	"Mus musc	RefSeq	4.544668	3.515421	2.040959	1750
TC0X00002	F8	NM_00797	"Mus musc	RefSeq	3.329671	4.358363	-2.04017	1752
TC0900000	Thy1	NM_00938	"Mus musc	RefSeq	7.039593	6.011624	2.039152	1753
TC1400000	Gm3127	XM_00147	"PREDICTEI	RefSeq	2.996508	4.024347	-2.03897	1754
TC0900002	St3gal4	NM_00917	"Mus musc	RefSeq	7.896112	6.86871	2.03835	1755
TC1100002	Rnf213	XM_00147	"PREDICTEI	RefSeq	8.331505	9.358506	-2.03778	1756
TC0900002	Nck1	NM_01087	"Mus musc	RefSeq	6.696242	7.722058	-2.03611	1757
TC0X00002	Zcchc5	NM_19946	"Mus musc	RefSeq	5.756805	4.731523	2.035357	1758
TC1300000	Gm2399	ENSMUST0	cdna:know	ENSEMBL	11.85571	10.8305	2.035265	1759
TC1800000	Sh3tc2	NM_17262	"Mus musc	RefSeq	5.511395	6.53659	-2.03523	1760
TC1400001	Gm3453	XM_00147	"PREDICTEI	RefSeq	3.158676	4.182899	-2.03386	1761
TC0900001	Myrip	NM_14455	"Mus musc	RefSeq	6.855976	5.832421	2.032922	1762
TC0500001	Tpst2	NM_00941	"Mus musc	RefSeq	6.97041	7.993832	-2.03274	1763
TC0300001	Bcar3	NM_01386	"Mus musc	RefSeq	5.811418	6.834578	-2.03237	1764
TC0700001	Prcp	NM_02824	"Mus musc	RefSeq	7.281807	8.303874	-2.03083	1765
TC0600000	Tmem150a	NM_14491	"Mus musc	RefSeq	7.62244	6.600388	2.030805	1766
TC0400003	Themis2	NM_00103	"Mus musc	RefSeq	5.010483	6.031977	-2.03002	1767
TC1100003	Acsf2	NM_15380	"Mus musc	RefSeq	6.468034	7.489325	-2.02973	1768
TC0200000	Abca2	NM_00737	"Mus musc	RefSeq	6.985242	5.96438	2.029131	1769
TC0200004	Prrg4	NM_17869	"Mus musc	RefSeq	4.980994	6.001856	-2.02913	1769
TC0100001	Cyb5r1	NM_02805	"Mus musc	RefSeq	6.519815	7.53974	-2.02781	1771
TC1100003	Krt222	NM_17294	"Mus musc	RefSeq	4.085369	5.104961	-2.02735	1772
TC1600000	Rtp4	NM_02338	"Mus musc	RefSeq	5.547175	6.566199	-2.02655	1773
TC0900002	Snx33	NM_17548	"Mus musc	RefSeq	7.51103	6.492116	2.026393	1774
TC1_GL456	AC125149.	ENSMUST0	cdna:know	ENSEMBL	6.950214	5.93185	2.025621	1775
TC0100000	Rdh10	NM_13383	"Mus musc	RefSeq	7.103802	6.085724	2.025219	1776
TC1400000	Gm3187	ENSMUST0	cdna:know	ENSEMBL	4.277374	5.294687	-2.02415	1777
TC0700002	Ehd2	NM_15306	"Mus musc	RefSeq	9.502195	10.51922	-2.02374	1778
TC0700003	Fes	NM_01019	"Mus musc	RefSeq	6.642705	7.659548	-2.02349	1779
TC0100003	Atp1b1	NM_00972	"Mus musc	RefSeq	5.477176	4.460432	2.023347	1780
TC0300000	Gpr160	NM_00113	"Mus musc	RefSeq	4.811287	5.827279	-2.02229	1781
TC0500000	Tbc1d1	NM_01963	"Mus musc	RefSeq	6.052938	7.068873	-2.02221	1782
TC0900001	Myo6	NM_00103	"Mus musc	RefSeq	6.933958	7.949608	-2.02181	1783
TC0700000	Cebpa	NM_00128	"CCAAT/en	RefSeq	6.696355	5.681594	2.020568	1784
TC0100000	1500015O1	NM_02428	"Mus musc	RefSeq	5.071549	4.057018	2.020246	1785

TC0800001	Ces2g	NM_19799	"Mus musc	RefSeq	6.112673	5.098272	2.020064	1786
TC1900001	Plgrkt	NM_02636	"Mus musc	RefSeq	6.581495	7.594861	-2.01862	1787
TC1300001	Atxn1	NM_00912	"Mus musc	RefSeq	6.895549	7.908103	-2.01748	1788
TC110000C	Spred2	NM_03352	"Mus musc	RefSeq	7.545492	8.557294	-2.01643	1789
TC1900001	Ostf1	NM_01737	"Mus musc	RefSeq	6.858213	7.868991	-2.015	1790
TC0800001	Adam5	NM_00127	"Mus musc	RefSeq	4.259878	3.249255	2.014781	1791
TC180000C	Ccny	NM_02648	"Mus musc	RefSeq	7.703239	8.713832	-2.01474	1792
TC130000C	Cd83	NM_00985	"Mus musc	RefSeq	6.549443	5.538932	2.014625	1793
TC190000C	Ifit1	NM_00833	"Mus musc	RefSeq	6.369125	7.378838	-2.01351	1794
TC0X00002	Gla	NM_01346	"Mus musc	RefSeq	6.41407	5.404486	2.01333	1795
TC0700004	Cox6a2	NM_00994	"Mus musc	RefSeq	6.442216	7.450894	-2.01207	1796
TC0200003	Uap1l1	NM_00103	"Mus musc	RefSeq	6.661127	5.652673	2.011754	1797
TC0400001	Dhrs3	NM_00117	"Mus musc	RefSeq	8.605393	9.613518	-2.0113	1798
TC1200001	Sec23a	NM_00914	"Mus musc	RefSeq	7.89993	6.891824	2.011269	1799
TC1200001	Hhipl1	ENSMUST0	cdna:know	ENSEMBL	6.053364	5.045535	2.010883	1800
TC140000C	Gm10413	NM_02928	"Mus musc	RefSeq	4.23376	5.240903	-2.00993	1801
TC0400003	Sepn1	NM_02910	"Mus musc	RefSeq	8.267408	7.260551	2.009528	1802
TC100000C	Sgk1	NM_00116	"Mus musc	RefSeq	8.111343	9.11799	-2.00924	1803
TC0400001	Lepre1	NM_01978	"Mus musc	RefSeq	6.774413	5.768509	2.008201	1804
TC010000C	Atic	NM_02619	"Mus musc	RefSeq	6.466695	5.460892	2.008061	1805
TC040000C	Tgfbr1	NM_00937	"Mus musc	RefSeq	7.757529	6.752258	2.007321	1806
TC0700001	Acadsb	NM_02582	"Mus musc	RefSeq	7.645353	6.640441	2.006821	1807
TC1900001	Aldh1a7	NM_01192	"Mus musc	RefSeq	4.856721	3.851982	2.00658	1808
TC0800003	Cbfa2t3	NM_17728	"Mus musc	RefSeq	6.975185	7.979395	-2.00585	1809
TC0X00003	Tspyl2	NM_02983	"Mus musc	RefSeq	7.550243	6.546653	2.004983	1810
TC0700003	Tsku	NM_00116	"Mus musc	RefSeq	5.893929	4.890955	2.004127	1811
TC1100002	Kremen1	NM_03239	"Mus musc	RefSeq	7.350108	6.347221	2.004006	1812
TC140000C	Flnb	NM_13408	"Mus musc	RefSeq	8.490359	9.492889	-2.00351	1813
TC190000C	Add3	NM_00116	"Mus musc	RefSeq	8.898536	7.896277	2.003134	1814
TC0500001	Cldn15	NM_02171	"Mus musc	RefSeq	5.563041	6.565296	-2.00313	1815
TC1000001	Syne1	NM_02202	"Mus musc	RefSeq	7.813306	8.81537	-2.00286	1816
TC1700001	Tnf	NM_01369	"Mus musc	RefSeq	5.550351	6.552236	-2.00262	1817
TC1_GL456	AC125149.	ENSMUST0	cdna:know	ENSEMBL	6.745165	5.74446	2.000978	1818

Gene Featu	Biotype
up	coding
up	coding
up	coding
up	coding
up	coding
up	coding
up	coding
up	coding
up	coding
up	coding
up	coding
up	coding
up	coding
up	coding
up	coding
up	coding
up	coding
up	coding
up	coding
up	coding
up	coding
up	coding
up	coding
up	coding
up	coding
up	coding
up	coding
up	coding
up	coding
up	coding
up	coding
up	coding
up	coding
up	coding
up	coding
up	coding
up	coding
up	coding
up	coding
up	coding
up	coding
up	coding
up	coding
up	coding
up	coding
up	coding

up	coding
up	coding
up	coding
up	coding
up	coding
up	coding
up	coding
up	coding
up	coding
up	coding
up	coding
up	coding
up	coding
up	coding
up	coding
up	coding
up	coding
up	coding
up	coding
up	coding
up	coding
up	coding
up	coding
up	coding
up	coding
up	coding
up	coding
up	coding
up	coding
up	coding
up	coding
up	coding
up	coding
up	coding
up	coding
up	coding
up	coding
up	coding
up	coding
up	coding
up	coding
up	coding
up	coding
up	coding
up	coding
up	coding
up	coding

up	coding
up	coding
up	coding
up	coding
up	coding
up	coding
up	coding
up	coding
up	coding
up	coding
up	coding
up	coding
up	coding
up	coding
up	coding
up	coding
up	coding
up	coding
up	coding
up	coding
up	coding
up	coding
up	coding
up	coding
up	coding
up	coding
up	coding
up	coding
up	coding
up	coding
up	coding
up	coding
up	coding
up	coding
up	coding
up	coding
down	coding
up	coding
up	coding
up	coding
up	coding
up	coding
up	coding
up	coding
up	coding
up	coding
up	coding

up	coding
up	coding
up	coding
up	coding
up	coding
up	coding
up	coding
up	coding
up	coding
up	coding
up	coding
up	coding
up	coding
up	coding
up	coding
up	coding
up	coding
up	coding
up	coding
up	coding
up	coding
up	coding
up	coding
up	coding
up	coding
up	coding
up	coding
up	coding
up	coding
up	coding
up	coding
up	coding
up	coding
up	coding
up	coding
up	coding
up	coding
up	coding
up	coding
up	coding
up	coding
down	coding
up	coding
up	coding
up	coding
up	coding
down	coding

up	coding
up	coding
up	coding
up	coding
up	coding
up	coding
up	coding
down	coding
up	coding
up	coding
down	coding
up	coding
up	coding
up	coding
up	coding
up	coding
up	coding
up	coding
up	coding
up	coding
up	coding
up	coding
up	coding
up	coding
up	coding
up	coding
up	coding
up	coding
up	coding
up	coding
up	coding
up	coding
up	coding
up	coding
up	coding
up	coding
up	coding
up	coding
up	coding
up	coding
up	coding
up	coding
up	coding
up	coding
up	coding
up	coding
up	coding

up	coding
up	coding
up	coding
up	coding
up	coding
up	coding
up	coding
down	coding
up	coding
down	coding
up	coding
up	coding
down	coding
up	coding
up	coding
up	coding
up	coding
down	coding
down	coding
up	coding
up	coding
down	coding
up	coding
up	coding
up	coding
up	coding
up	coding
down	coding
down	coding
up	coding
up	coding
up	coding
up	coding
up	coding
down	coding
up	coding
up	coding
up	coding
up	coding
up	coding
up	coding
up	coding
down	coding
up	coding
down	coding
up	coding
up	coding

up	coding
up	coding
down	coding
up	coding
up	coding
up	coding
down	coding
up	coding
down	coding
up	coding
down	coding
down	coding
up	coding
up	coding
up	coding
down	coding
down	coding
down	coding
up	coding
up	coding
up	coding
down	coding
up	coding
up	coding
up	coding
up	coding
down	coding
up	coding
up	coding
down	coding
down	coding
up	coding
up	coding
up	coding
up	coding
down	coding
down	coding
up	coding
up	coding
down	coding
up	coding
down	coding
down	coding
up	coding
up	coding
up	coding
up	coding

up	coding
up	coding
up	coding
down	coding
down	coding
down	coding
down	coding
down	coding
up	coding
up	coding
down	coding
down	coding
up	coding
up	coding
down	coding
down	coding
down	coding
down	coding
down	coding
down	coding
up	coding
up	coding
up	coding
down	coding
up	coding
down	coding
up	coding
up	coding
up	coding
down	coding
down	coding
up	coding
down	coding
down	coding
down	coding
down	coding
down	coding
down	coding
down	coding
down	coding
down	coding
up	coding
up	coding
down	coding
down	coding
down	coding
up	coding

down	coding
down	coding
down	coding
down	coding
down	coding
up	coding
up	coding
up	coding
down	coding
down	coding
down	coding
up	coding
down	coding
down	coding
down	coding
up	coding
up	coding
down	coding
up	coding
up	coding
down	coding
down	coding
down	coding
up	coding
up	coding
down	coding
down	coding
up	coding
down	coding
down	coding
down	coding
up	coding
down	coding
down	coding
up	coding
down	coding
up	coding
up	coding
down	coding
down	coding
down	coding
down	coding
up	coding
down	coding
up	coding
down	coding
up	coding

down	coding
up	coding
up	coding
down	coding
down	coding
down	coding
down	coding
down	coding
down	coding
down	coding
up	coding
down	coding
up	coding
down	coding
down	coding
up	coding
up	coding
up	coding
down	coding
up	coding
up	coding
up	coding
down	coding
down	coding
up	coding
down	coding
down	coding
up	coding
down	coding
up	coding
down	coding
up	coding
up	coding
up	coding
down	coding
down	coding
down	coding
down	coding
down	coding
down	coding
up	coding
down	coding
up	coding
down	coding
down	coding
down	coding
up	coding

down	coding
up	coding
down	coding
down	coding
down	coding
down	coding
down	coding
down	coding
down	coding
up	coding
down	coding
up	coding
down	coding
down	coding
down	coding
up	coding
up	coding
down	coding
down	coding
up	coding
down	coding
down	coding
down	coding
down	coding
down	coding
down	coding
down	coding
down	coding
down	coding
up	coding
down	coding
down	coding
up	coding
up	coding
down	coding
down	coding
up	coding
up	coding
down	coding
down	coding
up	coding
up	coding
down	coding
down	coding
up	coding
down	coding
up	coding

up	coding
down	coding
down	coding
up	coding
down	coding
down	coding
up	coding
up	coding
down	coding
down	coding
up	coding
up	coding
down	coding
up	coding
down	coding
down	coding
down	coding
up	coding
up	coding
down	coding
up	coding
up	coding
up	coding
down	coding
up	coding
up	coding
down	coding
up	coding
down	coding
up	coding
down	coding
down	coding
down	coding
down	coding
down	coding
up	coding
up	coding
up	coding
down	coding
up	coding
down	coding
down	coding
down	coding
down	coding
down	coding
down	coding
down	coding

down	coding
up	coding
down	coding
down	coding
down	coding
up	coding
down	coding
down	coding
down	coding
down	coding
down	coding
down	coding
up	coding
down	coding
up	coding
down	coding
down	coding
down	coding
up	coding
down	coding
up	coding
up	coding
down	coding
down	coding
down	coding
down	coding
down	coding
down	coding
up	coding
up	coding
up	coding
up	coding
up	coding
down	coding
down	coding
down	coding
down	coding
up	coding
up	coding
up	coding
down	coding
up	coding
down	coding
down	coding
down	coding
up	coding
down	coding

down	coding
up	coding
up	coding
down	coding
down	coding
up	coding
down	coding
down	coding
down	coding
up	coding
down	coding
down	coding
down	coding
down	coding
down	coding
down	coding
up	coding
up	coding
down	coding
up	coding
up	coding
down	coding
up	coding
up	coding
down	coding
up	coding
up	coding
up	coding
up	coding
down	coding
down	coding
up	coding
up	coding
down	coding
up	coding
up	coding
down	coding
down	coding
up	coding
down	coding
down	coding
down	coding
down	coding
up	coding
down	coding
down	coding
down	coding

up	coding
down	coding
up	coding
down	coding
down	coding
down	coding
up	coding
up	coding
down	coding
up	coding
up	coding
up	coding
up	coding
down	coding
down	coding
down	coding
down	coding
up	coding
up	coding
up	coding
down	coding
down	coding
down	coding
down	coding
up	coding
down	coding
down	coding
up	coding
down	coding
up	coding
down	coding
up	coding
up	coding
up	coding
down	coding
up	coding
up	coding
down	coding
up	coding
up	coding
down	coding
up	coding
up	coding
down	coding
down	coding
up	coding
down	coding

down	coding
down	coding
down	coding
down	coding
up	coding
down	coding
down	coding
up	coding
down	coding
down	coding
up	coding
down	coding
down	coding
up	coding
up	coding
up	coding
up	coding
up	coding
up	coding
up	coding
down	coding
up	coding
up	coding
down	coding
up	coding
down	coding
up	coding
up	coding
down	coding
down	coding
up	coding
up	coding
up	coding
up	coding
down	coding
down	coding
up	coding
down	coding
up	coding
down	coding
down	coding
down	coding
up	coding
down	coding
down	coding
down	coding
up	coding

down	coding
down	coding
up	coding
up	coding
up	coding
up	coding
down	coding
up	coding
down	coding
up	coding
down	coding
down	coding
up	coding
up	coding
down	coding
up	coding
down	coding
up	coding
down	coding
down	coding
up	coding
down	coding
up	coding
down	coding
down	coding
down	coding
up	coding
down	coding
down	coding
up	coding
up	coding
up	coding
up	coding
up	coding
down	coding
down	coding
up	coding
down	coding
down	coding
down	coding
down	coding
down	coding
down	coding
up	coding
down	coding
down	coding
up	coding

down	coding
down	coding
down	coding
down	coding
up	coding
up	coding
up	coding
down	coding
up	coding
down	coding
up	coding
up	coding
down	coding
up	coding
down	coding
up	coding
up	coding
down	coding
up	coding
up	coding
up	coding
down	coding
down	coding
down	coding
up	coding
up	coding
down	coding
down	coding
down	coding
down	coding
up	coding
up	coding
down	coding
up	coding
up	coding
up	coding
down	coding
down	coding
down	coding
down	coding
up	coding
up	coding
down	coding
down	coding
up	coding
up	coding
up	coding

up	coding
up	coding
up	coding
down	coding
down	coding
up	coding
down	coding
up	coding
up	coding
up	coding
down	coding
up	coding
up	coding
up	coding
down	coding
down	coding
down	coding
down	coding
up	coding
up	coding
up	coding
down	coding
down	coding
down	coding
down	coding
down	coding
down	coding
up	coding
down	coding
up	coding
down	coding
down	coding
up	coding
down	coding
up	coding
up	coding
down	coding
up	coding
down	coding
down	coding
down	coding
up	coding
down	coding
up	coding
down	coding
up	coding
down	coding

down	coding
up	coding
up	coding
down	coding
up	coding
up	coding
down	coding
down	coding
up	coding
down	coding
down	coding
up	coding
down	coding
up	coding
down	coding
up	coding
down	coding
up	coding
down	coding
down	coding
down	coding
down	coding
up	coding
down	coding
down	coding
down	coding
down	coding
down	coding
down	coding
down	coding
down	coding
down	coding
down	coding
up	coding
up	coding
up	coding
up	coding
down	coding
down	coding
down	coding
down	coding
up	coding
down	coding
down	coding
down	coding
down	coding
up	coding

down	coding
down	coding
down	coding
down	coding
down	coding
up	coding
up	coding
down	coding
down	coding
down	coding
down	coding
down	coding
down	coding
down	coding
down	coding
up	coding
down	coding
up	coding
down	coding
up	coding
down	coding
up	coding
up	coding
up	coding
down	coding
up	coding
up	coding
down	coding
down	coding
up	coding
up	coding
up	coding
up	coding
up	coding
down	coding
down	coding
up	coding
up	coding
down	coding
up	coding
down	coding
up	coding
down	coding
down	coding
down	coding
up	coding
up	coding

down	coding
down	coding
down	coding
up	coding
up	coding
down	coding
up	coding
down	coding
up	coding
up	coding
down	coding
down	coding
up	coding
down	coding
up	coding
up	coding
up	coding
up	coding
up	coding
up	coding
up	coding
up	coding
down	coding
down	coding
down	coding
down	coding
up	coding
down	coding
up	coding
down	coding
up	coding
down	coding
down	coding
up	coding
down	coding
up	coding
up	coding
up	coding
up	coding
up	coding
up	coding
up	coding
up	coding
up	coding
up	coding
down	coding
down	coding

up	coding
down	coding
up	coding
up	coding
down	coding
down	coding
up	coding
down	coding
down	coding
up	coding
down	coding
down	coding
up	coding
up	coding
down	coding
down	coding
up	coding
down	coding
down	coding
down	coding
down	coding
up	coding
up	coding
up	coding
up	coding
down	coding
up	coding
down	coding
up	coding
down	coding
up	coding
down	coding
down	coding
down	coding
down	coding
down	coding
up	coding
up	coding
down	coding
up	coding
down	coding
down	coding
down	coding
down	coding
up	coding
down	coding
down	coding

up	coding
down	coding
up	coding
up	coding
down	coding
up	coding
down	coding
down	coding
up	coding
down	coding
down	coding
up	coding
down	coding
down	coding
down	coding
down	coding
down	coding
up	coding
up	coding
down	coding
up	coding
up	coding
down	coding
up	coding
down	coding
up	coding
down	coding
down	coding
up	coding
up	coding
down	coding
up	coding
down	coding
down	coding
down	coding
up	coding
down	coding
down	coding
down	coding
up	coding
down	coding
down	coding
up	coding
up	coding
up	coding
up	coding
up	coding

down	coding
up	coding
down	coding
down	coding
up	coding
up	coding
up	coding
down	coding
up	coding
up	coding
down	coding
down	coding
down	coding
up	coding
up	coding
up	coding
up	coding
up	coding
down	coding
up	coding
down	coding
up	coding
down	coding
down	coding
down	coding
down	coding
up	coding
up	coding
down	coding
up	coding
up	coding
down	coding
down	coding
down	coding
down	coding
down	coding
down	coding
down	coding
down	coding
up	coding
up	coding
up	coding
down	coding
up	coding
up	coding
up	coding
down	coding

up	coding
up	coding
up	coding
up	coding
down	coding
down	coding
down	coding
down	coding
down	coding
up	coding
down	coding
up	coding
down	coding
down	coding
down	coding
up	coding
up	coding
down	coding
up	coding
up	coding
down	coding
up	coding
down	coding
down	coding
down	coding
up	coding
up	coding
up	coding
up	coding
up	coding
up	coding
up	coding
down	coding
down	coding
down	coding
down	coding
up	coding
up	coding
up	coding
down	coding
up	coding
up	coding
up	coding
up	coding
down	coding
down	coding
down	coding

up	coding
up	coding
up	coding
down	coding
up	coding
down	coding
down	coding
up	coding
down	coding
down	coding
up	coding
up	coding
down	coding
down	coding
down	coding
up	coding
down	coding
down	coding
up	coding
down	coding
up	coding
up	coding
up	coding
down	coding
up	coding
up	coding
down	coding
up	coding
up	coding
up	coding
up	coding
up	coding
down	coding
up	coding
down	coding
up	coding
up	coding
down	coding
down	coding
down	coding
up	coding
up	coding
up	coding
down	coding
down	coding
down	coding
up	coding

up	coding
down	coding
down	coding
up	coding
up	coding
down	coding
up	coding
up	coding
down	coding
up	coding
down	coding
down	coding
down	coding
up	coding
down	coding
up	coding
up	coding
up	coding
down	coding
down	coding
up	coding
down	coding
down	coding
up	coding
up	coding
down	coding
up	coding
down	coding
up	coding
down	coding
down	coding
up	coding
up	coding
up	coding
down	coding
up	coding
down	coding
down	coding
down	coding
up	coding
up	coding
down	coding
down	coding
down	coding
down	coding
up	coding
down	coding

down	coding
up	coding
down	coding
down	coding
up	coding
up	coding
up	coding
up	coding
down	coding
down	coding
up	coding
down	coding
up	coding
up	coding
up	coding
up	coding
up	coding
down	coding
down	coding
down	coding
up	coding
down	coding
up	coding
up	coding
up	coding
up	coding
down	coding
up	coding
up	coding
up	coding
down	coding
up	coding
down	coding
down	coding
up	coding
up	coding
up	coding
up	coding
up	coding
up	coding
down	coding
down	coding
down	coding
down	coding
up	coding
up	coding
up	coding

down	coding
down	coding
down	coding
down	coding
up	coding
down	coding
down	coding
down	coding
down	coding
up	coding
down	coding
up	coding
up	coding
down	coding
up	coding
up	coding
down	coding
down	coding
down	coding
down	coding
up	coding
up	coding
down	coding
up	coding
up	coding
down	coding
down	coding
up	coding
up	coding
up	coding
up	coding
up	coding
down	coding
down	coding
down	coding
down	coding
down	coding
down	coding
down	coding
down	coding
up	coding
up	coding
down	coding
down	coding
down	coding
down	coding
up	coding

down	coding
up	coding
down	coding
up	coding
down	coding
down	coding
up	coding
down	coding
down	coding
up	coding
down	coding
up	coding
up	coding
up	coding
up	coding
down	coding
down	coding
down	coding
up	coding
down	coding
down	coding
up	coding
up	coding
up	coding
down	coding
down	coding
down	coding
up	coding
down	coding
up	coding
up	coding
down	coding
down	coding
down	coding
up	coding
down	coding
down	coding
up	coding
up	coding
up	coding
up	coding
up	coding
down	coding
up	coding
down	coding
up	coding
up	coding

up	coding
up	coding
up	coding
up	coding
down	coding
down	coding
down	coding
down	coding
up	coding
down	coding
up	coding
down	coding
down	coding
down	coding
up	coding
down	coding
down	coding
down	coding
down	coding
up	coding
down	coding
down	coding
down	coding
up	coding
down	coding
down	coding
up	coding
down	coding
down	coding
up	coding
down	coding
up	coding
down	coding
up	coding
up	coding
up	coding
down	coding
down	coding
up	coding
down	coding
up	coding
down	coding
up	coding
up	coding
down	coding
down	coding
up	coding

up	coding
down	coding
down	coding
down	coding
down	coding
up	coding
down	coding
up	coding
down	coding
down	coding
up	coding
down	coding
down	coding
down	coding
up	coding
down	coding
up	coding
up	coding
down	coding
down	coding
down	coding
down	coding
down	coding
up	coding
down	coding
up	coding
up	coding
up	coding
up	coding
down	coding
down	coding
down	coding
up	coding
up	coding
down	coding
down	coding
down	coding
down	coding
down	coding
up	coding
down	coding
up	coding
down	coding
up	coding
down	coding
down	coding
up	coding

up	coding
up	coding
down	coding
up	coding
down	coding
down	coding
down	coding
up	coding
up	coding
up	coding
down	coding
down	coding
down	coding
down	coding
down	coding
down	coding
down	coding
up	coding
up	coding
up	coding
up	coding
up	coding
down	coding
up	coding
down	coding
down	coding
down	coding
down	coding
up	coding
up	coding
up	coding
up	coding
up	coding
down	coding
down	coding
up	coding
up	coding
up	coding
up	coding
up	coding
up	coding
down	coding
down	coding
down	coding
down	coding
up	coding
up	coding

down	coding
up	coding
up	coding
up	coding
up	coding
up	coding
down	coding
up	coding
down	coding
up	coding
up	coding
up	coding
down	coding
up	coding
down	coding
up	coding
down	coding
down	coding
up	coding
down	coding
down	coding
down	coding
up	coding
down	coding
up	coding
up	coding
up	coding
up	coding
up	coding
down	coding
down	coding
up	coding
up	coding
up	coding
up	coding
down	coding
down	coding
down	coding
up	coding
up	coding
down	coding
up	coding
up	coding
up	coding
down	coding
down	coding
down	coding

up	coding
down	coding
up	coding
down	coding
up	coding
down	coding
down	coding
down	coding
up	coding
down	coding
down	coding
down	coding
up	coding
down	coding
up	coding
down	coding
up	coding
up	coding
up	coding
up	coding
up	coding
down	coding
up	coding
down	coding
down	coding
up	coding
down	coding
up	coding
up	coding
down	coding
down	coding
down	coding
down	coding
down	coding
down	coding
down	coding
up	coding
down	coding
down	coding
up	coding
up	coding
down	coding
up	coding
up	coding
down	coding
down	coding
down	coding

up	coding
down	coding
down	coding
down	coding
down	coding
down	coding
up	coding
up	coding
up	coding
down	coding
up	coding
down	coding
down	coding
up	coding
up	coding
down	coding
down	coding
up	coding
up	coding
up	coding
up	coding
down	coding
down	coding
down	coding
down	coding
up	coding
up	coding
up	coding
up	coding
up	coding
up	coding
up	coding
up	coding
up	coding
up	coding
down	coding
up	coding
up	coding
up	coding
down	coding
up	coding
up	coding
down	coding
down	coding
up	coding
down	coding
down	coding

up	coding
down	coding
up	coding
up	coding
down	coding
up	coding
up	coding
up	coding
up	coding
up	coding
down	coding
up	coding
up	coding
down	coding
up	coding
down	coding
up	coding
down	coding
down	coding
up	coding
up	coding
down	coding
down	coding
up	coding
down	coding
down	coding
down	coding
up	coding
down	coding
down	coding
up	coding
down	coding
down	coding
down	coding
down	coding
up	coding
up	coding
up	coding
down	coding
down	coding
down	coding
up	coding
down	coding
down	coding
down	coding
up	coding
up	coding

up	coding
down	coding
down	coding
down	coding
down	coding
up	coding
down	coding
up	coding
down	coding
up	coding
down	coding
up	coding
down	coding
up	coding
up	coding
down	coding
up	coding
down	coding
up	coding
up	coding
up	coding
up	coding
up	coding
down	coding
up	coding
up	coding
up	coding
down	coding
up	coding
down	coding
down	coding
down	coding
up	coding
