# Supplementary material for: Differentiation-Associated MicroRNA Alterations in Mouse Heart-Derived Sca-1+CD31− and Sca-1+CD31+ Cells
Source: Stem Cells Int. 2016 May 19;2016:9586751. doi: 10.1155/2016/9586751 (PMC4889861; doi:10.1155/2016/9586751)
Supplement: Supplementary file 1 — Figure S1. Differentiation potential of Sca-1+CD31+cells by FACS into cardiomyocyte, smooth muscle, and endothelial cells in vitro. Table S1. The differently expressed miRNAs between Sca1+CD31−and Sca1+CD31+ cells. Table S2. KEGG pathway analyses of predicted miRNA targets. Table S3. Pathway of the differently expressed miRNAs (the top 20 pathway) and their corresponding target genes. Table S4. Cluster of the differently expressed miRNAs (the top 20 GO terms) and their corresponding target genes. Table S5. The differently expressed mRNAs between Sca1+CD31−and Sca1+CD31+ cells. Table S6. Pathway of the differently expressed mRNAs (the top 20 pathway) and their corresponding genes. Table S7. Cluster of the differently expressed mRNAs (the top 20 GO terms) and their corresponding genes. [file 9586751.f1.zip › Table S7.pdf]

GO ID	GO Name	ene Counts	e Amount in	richment Sc	p-value	FDR
GO:000715	cell adhesion	127	500	8.2896234	1.33E-77	5.12E-74
GO:000152	angiogenesi	70	189	12.087523	1.32E-55	2.54E-52
GO:000727	multicellular	142	954	4.8578159	4.78E-55	6.14E-52
GO:003015	cell different	101	636	5.1828106	8.78E-42	8.45E-39
GO:000828	negative reg	69	315	7.1489066	6.16E-38	4.74E-35
GO:004594	positive regu	104	745	4.5559416	7.48E-38	4.80E-35
GO:004306	positive regu	62	258	7.8428348	1.07E-36	5.91E-34
GO:000828	positive regu	73	382	6.2367822	7.15E-36	3.44E-33
GO:004306	negative reg	81	480	5.5073777	1.32E-35	5.64E-33
GO:003033	positive regu	45	129	11.38476	9.21E-35	3.55E-32
GO:000815	metabolic pr	109	991	3.5896651	2.82E-30	9.86E-28
GO:000739	nervous syst	60	307	6.3784324	3.18E-30	1.02E-27
GO:001631	phosphoryla	87	684	4.1511099	8.55E-29	2.53E-26
GO:000012	negative reg	77	561	4.4794939	9.49E-28	2.61E-25
GO:001647	cell migratio	38	125	9.921439	5.30E-27	1.36E-24
GO:000646	protein phos	79	611	4.2197524	1.06E-26	2.54E-24
GO:000750	heart develo	41	167	8.0125079	4.98E-25	1.13E-22
GO:000635	regulation of	145	1852	2.5552189	2.09E-24	4.48E-22
GO:000691	apoptotic pro	70	540	4.2306331	7.71E-24	1.56E-21
GO:000193	positive regu	24	49	15.985133	2.441E-23	4.70E-21

<table>
<tr><th>Gene Symbols</th><th>Rank</th></tr>
<tr><td>|Cdh23|Srpx2|Negr1|Cdh19|Pdpn|Prkca|Cdh11|Parvb|Thbs1|Svep1|Ret|Scarb2|Postn|Cx</td><td>1</td></tr>
<tr><td>|Tmem100|Mmp2|Meis1|Nos3|Nrp1|Robo4|Srpx2|Tnfaip2|Arhgap24|Prkd1|Ptk2|Prkd2|Ctg</td><td>2</td></tr>
<tr><td>|Sema3c|Lrp1|Sema6b|Wnt9a|Smo|Eya2|Dll1|Adcyap1r1|Spred2|Dact1|Ift81|Heyl|Efna5|F</td><td>3</td></tr>
<tr><td>|Dock7|Adcyap1r1|Pgf|Gata2|Slfn5|Sdc2|Slit3|Robo4|Hip1|Gli2|Musk|Cbfa2t3|Itgav|Sema</td><td>4</td></tr>
<tr><td>|Gli3|Hmox1|Timp2|Sox7|Cav1|Notch2|Tgfb2|Slfn3|Prkca|Smad3|Rgcc|Skap2|Wt1|Ptgs2|</td><td>5</td></tr>
<tr><td>|Plscr1|Nfix|Nr1h4|Mkl2|Pparg|Epas1|Lmo4|Hexb|Gli1|Tbx20|Lif|Cebpa|Pygo1|Foxs1|Mef</td><td>6</td></tr>
<tr><td>|Maged1|Casp1|Slit2|Alox12|G0s2|Sept4|Sfrp2|Anxa1|Map3k1|Wnt11|Bmp2|Bmp4|Uaca|</td><td>7</td></tr>
<tr><td>|Ptgs2|Kdr|Ddr2|Acer2|Adrb2|Il6ra|Bcl2l1|Vegfa|Ctgf|Igf1|Smo|Avpr1a|Rogdi|Ednrb|Hlx|Zf</td><td>8</td></tr>
<tr><td>|Sox9|Dpep1|Notch1|Bmp4|Pdgfrb|Cryab|Ctsh|Gimap5|Vimp|Timp1|Apoe|Foxc2|Smad3|C</td><td>9</td></tr>
<tr><td>|Ctsh|Pdgfra|Pld1|Egfr|Itgav|Ptp4a1|Snai1|Trip6|Ret|Irs1|Aqp1|Irs2|Zfp703|Has2|Smad3|C</td><td>10</td></tr>
<tr><td>|Ust|Isoc1|Pde5a|Bdh2|Gfod1|Enpp2|Acsf2|Arsb|Cd38|Alpl|Kifc3|Dusp3|Dusp6|Zdhhc18|</td><td>11</td></tr>
<tr><td>|Epha7|Atoh8|Igf1|Ndrg2|Efnb2|Ephb1|Mtss1|Ret|Nrp1|Srgap2|Hdac9|Mef2c|Camk1d|Efn</td><td>12</td></tr>
<tr><td>|Map3k11|Nlk|Ptpn3|Jak3|Prkd2|Rps6kl1|Flt1|Mst1r|Itpkb|Snrk|Ddr2|Pik3cb|Gk5|Pak1|Prk</td><td>13</td></tr>
<tr><td>|Nr1h4|Dach1|Tgif1|Dact1|Ripply3|Tbx20|Glis2|Bhlhe40|Cebpa|Efna1|Gli3|Cdkn1c|Eng|B</td><td>14</td></tr>
<tr><td>|Thbs1|Snai1|Fmnl3|Cd248|Nck2|Podxl|Kdr|Lrrc16a|Eng|Ctgf|Vegfa|Pdgfra|Lamb1|Nck1|</td><td>15</td></tr>
<tr><td>|Ephb1|Epha7|Jak3|Mapk11|Mertk|Prkd1|Tgfbr3|Pdgfra|Ddr2|Snrk|Sgk1|Ror1|Lyn|Prkab2</td><td>16</td></tr>
<tr><td>|Hdac9|Pkd1|Fbn1|Gli3|Tnnt2|Mkl2|Adap2|Pdlim3|Bmp4|Tgfbr1|Bmpr1a|Ptprj|Sema3c|Co</td><td>17</td></tr>
<tr><td>|Cdk8|Gata2|Nr4a1|Zfp354c|Mapk11|Nfkbiz|Heyl|Apbb2|Atf5|Ralgapa1|Eya4|Bhlhe40|Elk</td><td>18</td></tr>
<tr><td>|Tnfrsf21|Dram2|Xaf1|Tnfrsf12a|Dapk1|Tmem173|Epha7|Nr4a1|Tgfbr1|Gadd45b|Mef2c|G</td><td>19</td></tr>
<tr><td>|Egr3|Bmp4|Nrarp|Ccl11|Prkd1|Cav1|Fgf2|Pdgfb|Cav2|Ecm1|Kdr|Prkd2|Pgf|Cdh13|Cxcl1</td><td>20</td></tr>
</table>
